# An anonymized, de-identified registry study protocol to determine the effectiveness and safety of weight loss with enavogliflozin in patients with type 2 diabetes mellitus

**DOI:** 10.1371/journal.pone.0315603

**Published:** 2025-01-22

**Authors:** Hyunji Sang, Sunyoung Kim, Jiyoung Hwang, Selin Woo, Jaewon Kim, Dong Keon Yon, Sang Youl Rhee

**Affiliations:** 1 Department of Endocrinology and Metabolism, Kyung Hee University Medical Center, Kyung Hee University College of Medicine, Seoul, South Korea; 2 Center for Digital Health, Medical Science Research Institute, Kyung Hee University Medical Center, Kyung Hee University College of Medicine, Seoul, South Korea; 3 Department of Family Medicine, Kyung Hee University Medical Center, Kyung Hee University College of Medicine, Seoul, South Korea; 4 Department of Regulatory Science, Kyung Hee University, Seoul, South Korea; 5 Department of Precision Medicine, Kyung Hee University College of Medicine, Seoul, South Korea; 6 Department of Pediatrics, Kyung Hee University Medical Center, Kyung Hee University College of Medicine, Seoul, South Korea; University of Montenegro-Faculty of Medicine, MONTENEGRO

## Abstract

Sodium-glucose co-transporter 2 inhibitors, such as enavogliflozin, offer promising metabolic benefits for patients with type 2 diabetes (T2D), including glycemic control and improved cardiac function. Despite the clinical evidence, real-world evidence is needed to validate their safety and effectiveness. This study aims to evaluate the effects of weight loss and safety of enavogliflozin administration in patients with T2D in a real-world clinical setting over 24 weeks. This is a large-scale, prospective, multicenter, non-interventional observational study and will be conducted in 12 primary care centers nationwide between 2024 and 2026. Data will be collected at baseline, 12 weeks, and 24 weeks in a real-world clinical setting, including demographic details, clinical history, lifestyle habits, medication use, and various health indicators. Eligible participants are adults aged 19 to 80 with T2D and a body mass index (BMI) of ≥23 kg/m^2^ who are currently receiving treatment with Envlo (enavogliflozin) or Envlomet (enavogliflozin/metformin) tablets or planning to start treatment. The primary outcome is the change in BMI and body weight at 24 weeks from baseline. Secondary outcomes evaluated are changes in BMI and weight at 12 weeks, the proportion of participants achieving significant reductions in BMI and weight at 12 and 24 weeks, and body composition and glycemic improvements at 12 and 24 weeks. The study will analyze shifts in lipid profiles, liver and kidney functions, and body composition at 12 and 24 weeks as exploratory outcomes. For safety outcomes, the trial will prioritize the monitoring of adverse drug reactions and specific events of interest such as hypoglycemia, urinary tract infections, genital infections, polyuria, and polydipsia. This study design enables us to evaluate the effectiveness and safety of enavogliflozin for weight loss in a real-world setting while exploring its potential positive effects on cardiac function and metabolic risk factors in overweight or obese patients with T2D.

**Trial registration number:** ClinicalTrials.gov NCT06427083

## Introduction

The prevalence and mortality rates of diabetes mellitus continue to increase globally. In South Korea, the prevalence of diabetes among adults aged 30 and above was estimated to be 14.4% of the total population in 2016, approximately 5.01 million individuals [1]. In 2017, diabetes was the sixth leading cause of death in South Korea [2]. According to the United Kingdom Prospective Diabetes Study (UKPDS), a prospective cohort study of patients with type 2 diabetes (T2D) discovered that reducing glycated hemoglobin (HbA1c) by 1% in patients with T2D can lower the risk of microvascular complications by 37% and the risk of myocardial infarction by 14% [3,4]. Subsequent research confirmed that aggressive glycemic control in patients with T2D can prevent the onset of diabetic complications and delay the progression of existing complications [5].

Sodium-glucose co-transporter 2 (SGLT2) inhibitors cause a relative decrease in insulin secretion by increasing urinary glucose excretion and the glucagon/insulin ratio. This results in increased lipolysis and production of ketone bodies. The “super-fuel” hypothesis suggests that in diabetes, blood ketone bodies can serve as an effective energy source for the myocardium, potentially contributing to improved cardiac function [6,7]. These metabolic advantages suggest that this agent may offer benefits beyond treating diabetes alone, potentially addressing various metabolic risk factors, including obesity, hypertension, and dyslipidemia.

Enavogliflozin, developed by Daewoong Pharmaceutical Co., Ltd., is the first SGLT2 inhibitor approved in Korea for adjunctive use to improve glycemic control in patients with T2D. This was approved by the Ministry of Food and Drug Safety on November 30, 2022. The recommended dosage for enavogliflozin is 0.3 mg once daily for monotherapy or as an additional therapy alongside other antidiabetic agents, regardless of mealtime. Clinical studies have demonstrated the efficacy and safety of enavogliflozin. Enavogliflozin exhibits potent and prolonged urinary glucose excretion, effectively reducing HbA1c and fasting plasma glucose (FPG) levels [8,9]. These findings highlight the potential of enavogliflozin as an effective and safe treatment option for T2D, offering additional metabolic benefits beyond glycemic control.

However, real-world data on the efficacy and safety of medications such as enavogliflozin in clinical practice are scarce. Hence, studies assessing the therapeutic effects and safety profile of enavogliflozin in actual clinical settings are warranted. Such research should particularly emphasize real-world evidence to supplement the existing knowledge on its glycemic effects, weight reduction, and other metabolic parameters in Korean patients. This study is designed for academic purposes to provide real-world evidence from domestic users for various metabolic indicators, such as body weight and body fat, in addition to the hypoglycemic effects of Envlo tablets.

## Materials and methods

### Study design

This study is designed as a large-scale, prospective, multicenter, non-interventional observational study to assess the efficacy and safety of Envlo tablets/Envlomet sustained-release (SR) tablets in patients with T2D over 24 weeks.

### Study setting

This study will involve 12 primary care centers nationwide and will focus on the impact of medication on weight loss, among other health outcomes. Eligible patients include those diagnosed with T2D who are candidates for treatment with Envlo or Envlomet SR tablets based on the medical judgment of the participating healthcare providers. All participation in the study is predicated on obtaining voluntary informed consent from the patients, highlighting the study’s adherence to ethical research practices.

**Fig 1:**
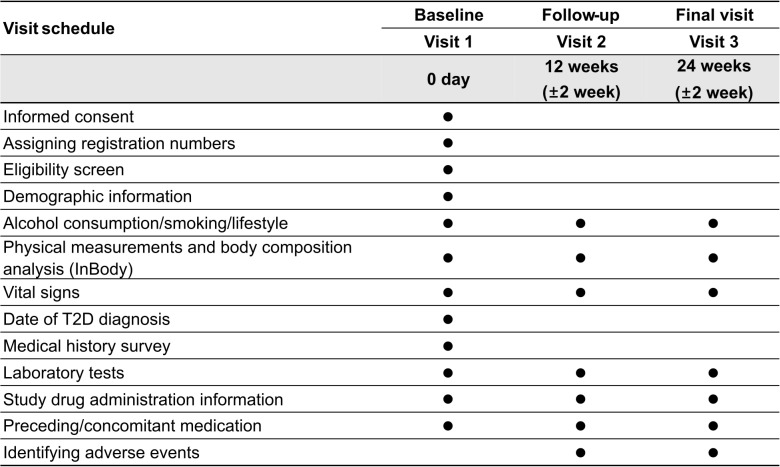
Clinical study data collection timeline. T2D, type 2 diabetes mellitus.

Participants will be enrolled from 23/05/2024 to 31/12/2026. Data will be collected up to 24 weeks after the administration of the treatment, capturing a wide array of information, such as demographic details, clinical history, lifestyle habits, medication use, and various health indicators (e.g., weight, body mass index [BMI], body composition, blood glucose levels, blood pressure, lipid profiles, and safety-related outcomes), directly from medical records in a real-world setting. The investigators will collect data for the study using information obtained during routine care. Depending on the clinical setting, prospective follow-ups will be conducted at 12 (±2 weeks) and 24 weeks (±2 weeks) after enrollment (visit 1, baseline, day 0) (Fig 1).

The protocol was developed in accordance with the Standard Protocol Items: Recommendations for Interventional Trials (SPIRIT) 2013 Statement (S1 File) [10].

### Eligibility criteria

Participants will be selected based on the following inclusion criteria: adults aged between 19 and 80 years, diagnosed with T2D, regardless of whether they are newly diagnosed or have been previously diagnosed and treated. They will be initially administered Envlo or Envlomet SR tablets, as determined by the researcher’s (physician’s) medical judgment. These include patients scheduled for enavogliflozin monotherapy, dual therapy with enavogliflozin and metformin, or triple therapy with enavogliflozin, metformin, and a dipeptidyl peptidase-4 (DPP-4) inhibitor. Additionally, participants will include individuals who are classified as pre-obese (overweight) or obese according to the 2022 Korean Endocrine Society’s clinical guidelines for obesity management [11]. These categories encompass pre-obese (BMI 23–24.9 kg/m^2^), obesity class I (BMI 25–29.9 kg/m^2^), obesity class II (BMI 30–34.9 kg/m^2^), and obesity class III (BMI ≥35 kg/m^2^). Eligible participants are those who intend to follow an appropriate exercise and diet regimen for blood sugar control during the study period. They must agree to use effective contraception or confirm they are not planning pregnancy during the study period. Moreover, participants must provide voluntary written consent to participate in the study after receiving and understanding detailed information about the observational research and the characteristics of the study medication.

Individuals will be excluded from participation in this clinical study if they meet any of the following criteria: those diagnosed with forms of diabetes other than T2D, including type 1 diabetes mellitus, diabetic ketoacidosis, gestational diabetes, and others; patients for whom treatment with an Envlo tablet or Envlomet SR tablet is contraindicated according to approved indications; patients who have undergone treatments for obesity or other treatments such as surgery or diet that have resulted in unstable weight within the 3 months before enrollment; individuals considered psychosomatically weak; pregnant or breastfeeding women; individuals currently participating in another clinical trial and receiving investigational medicinal products or medical devices; and any other individuals deemed by the researcher (attending physician) to be inappropriate for participation in the trial based on their professional judgment.

### Treatment

Envlo (enavogliflozin 0.3 mg) and Envlomet SR tablets (enavogliflozin 0.3 mg, metformin 1,000 mg) are manufactured by Daewoong Pharmaceutical Co. Additionally, both drugs are administered as adjuncts to diet and exercise therapy to improve glycemic control in patients with T2D. Both agents are administered as a single tablet once daily and should be stored at room temperature (1–30 °C) in a tightly sealed container.

The investigator (treating physician) will determine the administration of the study drug (Envlo or Envlomet tablets) and the choice of the therapeutic dose in a real-world practice setting, considering the licensing of the study drug and the participant’s medical condition.

### Outcomes

The primary outcome is assessed as the change in BMI and body weight at 24 weeks from baseline. Secondary outcomes include changes in BMI and weight from baseline to the 12-week point, along with the proportion of participants who achieve a reduction of at least 5% in BMI and weight at 12 and 24 weeks. Additionally, alterations in body composition metrics such as body fat mass, visceral fat mass, muscle mass, and waist-to-hip circumference are evaluated at 12 and 24 weeks from baseline. The study also measures changes in HbA1c and FPG levels at both the 12-week and 24-week time points relative to baseline. Furthermore, we examine the percentage of participants who achieve HbA1c levels below 7% and 6.5% at the 12 and 24-week time points, respectively. Finally, the proportion of participants achieving a therapeutic response, defined as either a decrease in HbA1c greater than 0.5% or an HbA1c <7%, is assessed at 12 and 24 weeks compared to baseline.

The exploratory outcomes include assessing changes from baseline to both 12- and 24-week marks across several parameters. First, the study examines the shifts in lipid profiles, including total cholesterol, low-density lipoprotein cholesterol, high-density lipoprotein cholesterol, and triglycerides. Additionally, changes in liver function markers, such as aspartate aminotransferase, alanine aminotransferase, and gamma-glutamyl transferase, are also evaluated. The study also focuses on kidney function, with an assessment of changes in indicators such as estimated glomerular filtration rate (eGFR), urine albumin-to-creatinine ratio, and urine glucose-to-creatinine ratio. Finally, the study examines changes in various body composition indicators such as body water, intracellular water, extracellular water, extracellular to intracellular water ratio, and abdominal fat ratio.

**Fig 2:**
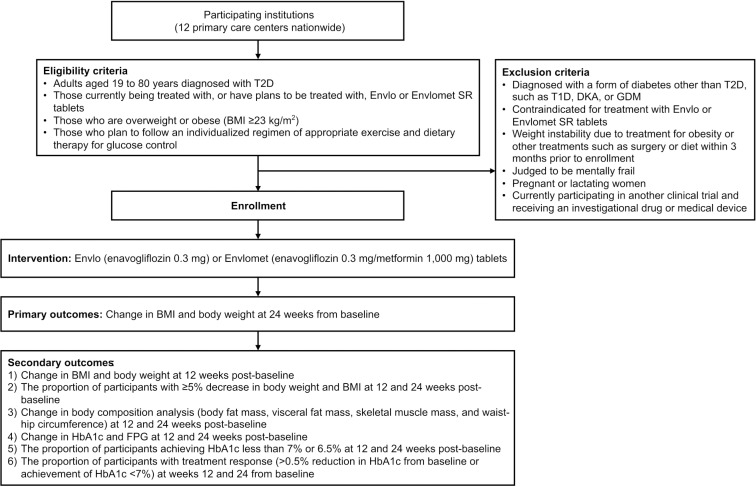
Study flowchart. T2D, type 2 diabetes mellitus; SR, sustained release; BMI, body mass index; T1D, type 1 diabetes mellitus; DKA, diabetic ketoacidosis; GDM, gestational diabetes; eGFR, estimated glomerular filtration rate; HbA1c, glycated hemoglobin; FPG, fasting plasma glucose.

Safety outcomes are centered on monitoring the incidence and number of adverse drug reactions (ADRs) and serious adverse drug reactions (SADRs) to assess a drug’s safety profile. Additionally, the study particularly pays attention to specific adverse events of interest, such as hypoglycemia, urinary tract infections, genital infections, polyuria, and polydipsia, to identify any potential risks associated with treatment. The evaluation includes laboratory tests, vital signs, and physical examinations. Fig 2 displays the flowchart for the study timeline.

### Sample size

This is a single-group observational study in which the mean and standard deviation of the changes in weight and BMI from baseline at 12 or 24 weeks were calculated using G*Power 3.1.9.7 [12], referencing the outcomes of previously conducted phase 2 and phase 3 clinical trials.

Based on previous studies, intervention with Envlo tablets or Envlomet SR tablets resulted in a mean weight change of 2.5 kg with a standard deviation of 10.0, yielding an effect size of 0.25. With a power of 0.95 and an α error of 0.05, the total required sample size is estimated to be approximately 210 participants. Anticipating a dropout rate of approximately 15%, this study aims to recruit approximately 240 participants.

If the dropout rate exceeds expectations, an interim analysis will be conducted to assess the clinical significance concerning the primary endpoints and safety assessments. If this analysis does not yield sufficiently conclusive results, consideration will be given to extending the recruitment period or enrolling additional participants, as needed, in consultation with the sponsor and steering committee.

### Data collection

In this study, data will be collected using a predesigned electronic case report form (eCRF), and all electronic data capture (EDC) systems will comply with the U.S. Code of Federal Regulations for processing and managing electronic data. The EDC system, a certified electronic data collection system, is accessible only to authorized individuals. All actions will be recorded by entering, modifying, saving, and deleting data in the eCRF using the EDC system. The collection schedules for all data collection items are presented in Fig 1.

#### 1) Obtaining written consent and assigning participant registration number.

Before collecting any data related to this study, the researchers will thoroughly explain the purpose and details of the study to potential participants using a participant information sheet. Participants will be asked to provide voluntary consent, and a consent form, including the participant’s name, signature, and date of signature, will be acquired. Although the dates of written consent and the initial visit may differ, consent must be obtained before participation in the clinical study commences. After obtaining consent from the participants, a unique registration number will automatically be assigned to each participant in the eCRF upon registration.

#### 2) Inclusion and exclusion criteria verification.

The inclusion and exclusion criteria are verified at the first visit, ensuring that each participant meets all the inclusion criteria and does not meet any of the exclusion criteria.

#### 3) Demographic Information.

At the initial visit, basic information about the participants is collected for verification. The information encompasses initials, sex, date of birth, and age, as well as details regarding pregnancy and breastfeeding. Pregnancy status is determined through interviews or, if available, based on pregnancy test results.

#### 4) T2D information.

At the first visit, the date of T2D diagnosis is collected from the participants to confirm the appropriateness of administering Envlo or Envlomet SR tablets.

#### 5) Medical history investigation.

Clinically significant medical conditions or abnormalities observed up to the first visit are defined and recorded. This includes the history of illnesses within 6 months before the initial visit and any ongoing conditions, along with their diagnoses and dates.

#### 6) Alcohol consumption, smoking, and lifestyle information.

At the initial visit, basic information regarding the participants’ alcohol consumption, smoking habits, and lifestyle is collected for verification. Subsequently, changes in these areas are recorded during each visit.

Alcohol consumption is categorized as “current drinker,” “past drinker,” or “never drinker.” “Current drinker” is defined as someone who has consumed 12 or more units of alcohol in their lifetime and has been drunk at least once in the past 12 months. A “past drinker” refers to someone who consumed 12 or more units of alcohol in their lifetime but has not been drunk in the past 12 months. A “never drinker” is defined as someone who has consumed less than 12 units of alcohol in their lifetime [13]. For current drinkers, the number of drinks consumed per week and the amount of alcohol consumed in a single sitting are recorded.

We categorize smoking history as “current smoking,” “past smoking,” or “non-smoking,” where “current smoking” is defined as having smoked five or more packs (100 cigarettes) in a lifetime and within the last 30 days. “Past smoking” is defined as having smoked five or more packs (100 cigarettes) in a lifetime but not within the past 30 days. Additionally, “non-smoking” is defined as having smoked less than five packs (100 cigarettes) in a lifetime [14].

In the study, lifestyle information includes eating habits such as irregular eating, overeating, and excess carbohydrates/sugar, fat, and salt intake. Exercise habits include the type of exercise (walking, cardio, or strength training), number of times per week, and intensity (less than 30 min, less than an hour, or longer).

#### 7) Physical measurements.

Body weight is measured at each visit, meanwhile, height is measured only once at the first visit.

#### 8) Vital signs.

At every visit, vital signs including blood pressure (systolic/diastolic) and pulse rate are also recorded. Blood pressure and pulse rate measurements are obtained after at least 5 min of rest in a quiet environment while sitting in a chair with back support. Participants are advised to avoid smoking, alcohol, and caffeine consumption for at least 30 min before the measurement.

#### 9) Body composition analysis.

Body composition analysis is performed employing the InBody770 and will be performed at every visit, depending on availability. The metrics include skeletal muscle mass, body fat mass, body fat percentage, muscle mass, waist-to-hip ratio, total body water, intracellular water, extracellular water, extracellular-to-intracellular water ratio, and visceral fat percentage.

#### 10) Concomitant and preceding medication.

For preceding medications, only treatments for diabetes administered within 4 weeks before the initial visit are collected. Regarding concomitant medications, the analysis includes information on all medicines taken consistently for more than three months during the study period after the first visit, including those for managing T2D. The collected information includes the name of the medication (brand name), dosage and administration details (single dosage, unit, frequency of administration, and route of administration), duration of treatment (start date, end date, and whether it was ongoing), purpose of administration, and reasons for dose changes or discontinuation, if applicable.

#### 11) Laboratory tests.

Laboratory tests are obtained based on medical records if performed in a real-world setting according to standard clinical practices. If HbA1c results collected within 4 weeks before the first visit and other test results collected within 3 months are available, these can be substituted for the laboratory tests at the initial visit.

The homeostatic model assessment of β-cell function (HOMA-beta) is calculated by applying the formula [360 × Fasting insulin (μU/mL) × FPG (mg/dL) − 63], and the homeostatic model assessment of insulin resistance (HOMA-IR) is calculated by applying the formula [Fasting insulin (μU/mL) × FPG (mg/dL)/405] [15].

#### 12) Adverse events (AEs).

AEs are recorded throughout the study period from the time the study drug is administered until the study concludes. This ongoing assessment helps evaluate the safety of the investigational product (IP) and ensures the safety of the participants. The collected information includes data on the AEs, including the occurrence, details, names, dates of onset and resolution, severity, whether the AE is unexpected, and progress. Additionally, information is gathered on the causality with the IP, actions taken related to the IP, actions taken independent of the IP, and the investigator’s assessment.

An AE is defined as any unfavorable and unintended sign (including an abnormal laboratory test finding), symptom, or disease occurring in a participant after obtaining informed consent that does not necessarily have a causal relationship with the IP. An ADR refers to any untoward or unintended response to an IP of any dose, where a causal relationship with the IP cannot be excluded. An SAE or SADR refers to an AE or ADR that results in any of the following outcomes: (1) death, (2) hospitalization or prolongation of existing hospitalization, (3) congenital anomaly or birth defect, (4) life-threatening condition, (5) significant disability or incapacity, or (6) any other medically significant condition. However, the following cases are not considered SAEs: planned hospitalization for the treatment of a pre-existing condition that has not worsened, hospitalization scheduled before the study subject signed informed consent, elective hospitalization, or emergency room visits without subsequent hospitalization. An unexpected AE is defined as an AE not reflected in the drug’s precautions. An unexpected ADR is an ADR that differs in nature, severity, specificity, or outcome from the drug’s approved marketing information.

The severity of AEs will be classified according to the following criteria: (1) Mild: Subjective or objective symptoms are present but do not interfere with daily activities; (2) Moderate: Symptoms interfere with daily activities to the extent that treatment is required; and (3) Severe: Symptoms prevent normal daily activities and require medical intervention, which may include hospitalization. The outcome of each AE will be recorded using the following categories: (1) recovered/resolved, (2) recovering/resolving, (3) not recovered/not resolved, (4) recovered/resolved with sequelae, (5) death due to ADR/AE (fatal), and (6) unknown.

The causal relationship between the investigational product (IP) and the onset of AEs will be determined by the investigator based on clinical judgment. Causality will be assessed using the following categories: certain, probable, possible, unlikely, not related, conditional/unclassified, and unassessable/unclassifiable. If evaluated as certain, probable, possible, conditional/unclassified, or unassessable/unclassifiable, the event will be considered to be related. If evaluated as unlikely or not related, the event will be considered unrelated. Details of the assessment criteria for causal relationships are provided in the S1 Table.

In addition, the actions taken regarding IP in response to an AE will be documented, including whether the drug is withdrawn, the dose is reduced, the dose is increased, or the dose remains unchanged. The following categories will be used: (1) drug withdrawn, (2) dose reduced, (3) dose increased, (4) dose not changed, (5) unknown, and (6) not applicable. Further, the actions taken to address the AE itself will be recorded. These actions may include: (1) concomitant medication therapy, (2) non-medication therapy, (3) concomitant medication therapy/non-medication therapy, or (4) no concomitant medication therapy/non-medication therapy.

AEs that occur at the third visit or are ongoing will be followed up until resolution or until the investigator (attending physician) deems further follow-up unnecessary. For participants who withdraw from the study within 24 weeks, efforts will be made to collect as much information as possible on AEs that occurred up to 30 days after withdrawal.

### Data management

The quality of the collected data will be verified through periodic audits. In case of discrepancies between the eCRF and source documents, inappropriate entries, or logical inconsistencies, the sponsor or data management personnel will collaborate with the principal investigator to review the validity of the concerned items. Corrections will be made through documentation to ensure the accuracy of the records. Once the sponsor confirms that the eCRF and database are error-free, the database will be locked to prevent accidental or unauthorized changes in the data.

The sponsor has the right to request the verification or modification of the collected data during the data processing phase. Upon receiving such requests, researchers must recheck or amend the data accordingly to ensure that their responses align with the request. This guarantees that the data entered into the eCRF through electronic signatures are accurate, complete, decipherable, and timely. The eCRFs created via the EDC system will be copied to electronic storage media and distributed to each research institution upon the conclusion of the study.

The principal investigator is responsible for storing and managing all data and records collected at each institution, including those of participants who might withdraw their consent or drop out midway. All documents collected during the study period will be stored in a secure location accessible only to the principal investigator, co-investigators, and designated research personnel and equipped with security measures to prevent unauthorized access. According to Article 15 of the regulations on bioethics and safety, the principal investigator must retain these documents for 3 years from the date of study completion, with the possibility of extending this period if necessary, by the sponsor. These documents may be subject to inspection by the sponsors or relevant regulatory authorities. Without written permission from the sponsor, the researchers may not destroy any documents related to this study. Upon the expiration of the retention period, in consultation with the sponsor, paper documents will be shredded immediately, and electronic records will be destroyed so that they cannot be restored or regenerated.

### Statistical methods

Continuous variables will be described using descriptive statistics (number of participants, mean, standard deviation, median, minimum, and maximum), and categorical variables will be presented as frequencies and percentages. Descriptive statistics for each variable within the primary, secondary, exploratory, and safety outcomes will be provided at baseline as well as at 12 and 24 weeks. To assess the differences across the three time points (baseline, 12 weeks, and 24 weeks), we will use a repeated-measures analysis of variance (ANOVA). The Bonferroni correction will be applied to control for multiple comparisons to ensure a more accurate assessment of changes over time. Paired *t*-tests or Wilcoxon signed-rank tests will be performed depending on the data distribution for differences at each time point compared with the baseline. All tests will be conducted using a two-sided approach, with a significance level of 5% [16–18].

Randomization minimizes the influence of potential confounders such as dietary habits and concomitant medications by ensuring that clinical variables other than the assigned treatment are not statistically significant. Additionally, statistical adjustment with multivariate and stratified analyses will be performed to validate the significance of any potential confounders, such as prior treatment or the duration of T2D, that may have affected the primary endpoints.

During the study period, statistical analyses will be conducted on the data collected on AEs and ADRs, including SAEs/SADRs, and unexpected AEs/ADRs. The analysis will present the number of participants experiencing AEs, the number of events, incidence rates, and 95% confidence intervals. Additionally, a summary of the AEs will be provided, detailing their severity, causality with the IP, actions taken regarding the IP, actions taken unrelated to the IP, and the outcome of the AEs based on the predefined classification system.

### Data monitoring

The sponsor conducts monitoring to protect the rights and welfare of the research participants; verifies the accuracy, completeness, and verifiability of the study data reported by the principal investigator through comparison with source documents; and ensures that the study is conducted according to the approved protocol and relevant regulations. The study will be monitored through regular visits and telephone communications with monitoring personnel designated by the sponsor at the study institutions. During the visits, the monitoring personnel will primarily review the source documents, management records of the study software, and the storage status of essential study documents. They will also assess the progress procedures and records of the study, discuss any issues such as violations with the principal investigator and study personnel, and ensure that appropriate modifications and actions are taken.

### Patient and public involvement

Patients did not participate in the study design and will not be involved in the recruitment or conduct of the study.

### Ethics and dissemination

This protocol was approved by the Public Institutional Review Board (IRB) designated by Ministry of Health and Welfare (IRB no. P01-202405-01-033, approved on May 23, 2024).

The researcher obtains informed consent from the participants based on the ethical principles of the Declaration of Helsinki and the standards of the Bioethics and Safety Act. The researcher thoroughly explains the research to the participants (or legal representatives) and obtains written consent before beginning any research-related procedures. Consent is received privately (e.g., in an office or consultation room).

The data will be stored in a locked laboratory. Participants’ medical record numbers and institutional registration numbers will be maintained in a separate file under the responsibility of the principal investigator. These identifiers will be encrypted to ensure that the clinical study data remains de-identified. According to the Enforcement Rule of Bioethics and Safety Act, records related to clinical research will be retained for 3 years after the completion of the study, and documents containing personal information will be destroyed after the retention period, as per the Personal Information Protection Act. Files containing personal information will be password-protected and any publication of clinical study results will exclude information that could potentially identify patients.

Researchers will verify the health status of each participant before enrolling them in the clinical trial to ensure their suitability for participation. Treatment and care for participants’ conditions must proceed independently of the trial, with the necessary medical treatment and care provided under clinical judgment during and after the trial period. This trial does not present any risks beyond those associated with routine care in a clinical setting; thus, additional compensation is not needed for study-related risks. Participants and researchers are adequately protected by medical law, the principal investigator’s professional indemnity insurance, and related institutional policies. The existing legal liabilities of medications govern the compensation for pharmaceuticals.

The sponsor owns all the data and results obtained through the study, and with the sponsor’s approval, the study results will be presented as academic papers at national and international conferences. If the study protocol requires modifications, we plan to obtain IRB approval for amendments at each clinical trial stage.

## Discussion

The strength of this study lies in its large-scale, prospective, multicenter, non-interventional observational design, which allows the evaluation of the effects of enavogliflozin on weight loss and its safety in a real-world clinical setting. This approach not only provides a comprehensive understanding of the impact of enavogliflozin beyond glycemic control but also offers insights into the potential benefits of the drug on improved cardiac function and metabolic risk factors in patients with T2D who are overweight or obese. The real-world setting of this study is crucial as it reflects the actual use of the drug in routine clinical practice, thereby providing valuable data on its effectiveness and safety profile among a broad and diverse patient population.

However, the non-interventional observational design of this study has certain limitations. Without a control group, it would be challenging to attribute the observed changes directly to enavogliflozin without considering other confounding factors that could influence the outcomes, such as concurrent medications, dietary habits, physical activity levels, or other lifestyle changes during the study period. Additionally, reliance on self-reported data from participants may introduce bias or inaccuracy in reporting AEs, compliance, or changes in lifestyle factors. Third, because the sponsor commissioned this study, we are constrained by a limited budget, making it challenging to include a direct comparison with other medications, such as semaglutide. However, we are considering future analyses using the data collected in this study to compare the efficacy and safety of enavogliflozin with those of other medications. Finally, the focus of the study on a specific patient population (those with T2D and overweight/obese individuals) may limit the generalizability of the findings to all patients with T2D.

## Conclusions

In conclusion, this study underscores the potential of enavogliflozin as a safe and effective treatment option for weight management in patients with T2D. By promoting weight loss and glycemic control, enavogliflozin offers a dual therapeutic benefit, addressing both metabolic and cardiovascular risk factors. Clinically, the results of this protocol, when validated, may provide valuable real-world evidence supporting the use of enavogliflozin in the routine care of overweight and obese patients with T2D, potentially influencing treatment guidelines and improving patient outcomes in everyday practice.

## Supporting information

S1 File SPIRIT 2013 checklist. (DOCX)

S1 Table The assessment criteria for the causal relationship between the administration of the investigational product and the onset of the adverse event. (DOCX)

S1 Protocol Study protocol (original document). (DOCX)

S2 Protocol Study protocol (English translation). (DOCX)
