# Supplementary material for: An anonymized, de-identified registry study protocol to determine the effectiveness and safety of weight loss with enavogliflozin in patients with type 2 diabetes mellitus
Source: PLoS One. 2025 Jan 22;20(1):e0315603. doi: 10.1371/journal.pone.0315603 (PMC11753631; doi:10.1371/journal.pone.0315603)
Supplement: S1 Table — (DOCX) [file pone.0315603.s002.docx]

**S1 Table. The assessment criteria for the causal relationship between the administration of the investigational product and the onset of the adverse event.**

| **Classification** | **Details** |
| --- | --- |
| Certain | A plausible temporal relationship with the IP, not explained by other drugs, chemicals, or underlying conditions. A clinically reasonable response to the withdrawal of the IP, and a pharmacologically or phenomenologically definitive response to re-administration, if necessary. |
| Probable | A reasonable temporal relationship with the IP, unlikely to be explained by other drugs, chemicals, or underlying conditions. A clinically reasonable response to the withdrawal of the IP (without re-administration information). |
| Possible | A reasonable temporal relationship with the IP, but could also be explained by other drugs, chemicals, or underlying conditions. Information about withdrawal of the IP is lacking or unclear. |
| Unlikely | A temporary case with an improbable causal relationship with the administration/use of the IP, and can be reasonably explained by other drugs, chemicals, or underlying conditions. |
| Not Related | No administration of the IP, or the temporal relationship of the adverse event is not plausible, or the adverse event can be more likely explained by other factors. |
| Conditional/Unclassified | More data is needed for appropriate evaluation, or additional data is under review. |
| Unassessable/Unclassifiable | Information is insufficient or conflicting, making a judgment impossible, and cannot be supplemented or verified. |

IP, Investigational product.
