# Supplementary material for: An anonymized, de-identified registry study protocol to determine the effectiveness and safety of weight loss with enavogliflozin in patients with type 2 diabetes mellitus
Source: PLoS One. 2025 Jan 22;20(1):e0315603. doi: 10.1371/journal.pone.0315603 (PMC11753631; doi:10.1371/journal.pone.0315603)
Supplement: S1 Protocol — (DOCX) [file pone.0315603.s003.docx]

**관찰연구계획서**

**2형 당뇨병 환자에서 엔블로정 또는 엔블로멧서방정 투여에 따른 체중 감소 효과 및 안전성을 확인하기 위한 익명화 비식별화 레지스트리 연구**

**An anonymized, de-identified registry study to determine the effectiveness and safety of weight loss with Enavogliflozin in patients with type 2 diabetes mellitus**

| **연구대상의약품:** | **엔블로정 0.3 밀리그램**  **엔블로멧서방정0.3/1000 밀리그램** |
| --- | --- |
| **연구계획서 번호:** | **DW_ODNENV_DB_02** |
| **연구계획서 버전:** | **V1.0** |
| **개발자:** | **㈜오디엔** |
| **의뢰자:** | **㈜대웅제약** |
| **연구계획서 작성일:** | **2024-05-16** |

**연구계획서 제·개정 이력**

| **No.** | **Version No.** | **Version Date** | **변경 내용** |
| --- | --- | --- | --- |
| 1 | 1.0 | 2024-05-16 | 해당사항 없음(제정) |

**의뢰자**

| 회사명 | ㈜대웅제약 | | |
| --- | --- | --- | --- |
| 의뢰책임자 | 이창재, 박성수 | | |
| 주소 | 서울특별시 강남구 봉은사로 114길 12 | 우편번호 | 06170 |
| 연락처 | 02-550-8800 | | |

**연구계획서 요약**

| **연구계획서 번호** | DW_ODNENV_DB_02 |
| --- | --- |
| **관찰연구 제목** | 2형 당뇨병 환자에서 엔블로정 또는 엔블로멧서방정 투여에 따른 체중 감소 효과 및 안전성을 확인하기 위한 익명화 비식별화 레지스트리 연구 |
| **연구계획서 버전** | V1.0 |
| **작성일** | 2024-05-16 |
| **단계 및 디자인** | 전향적, 다기관 관찰연구 |
| **관찰연구 실시기관** | 관찰연구를 실시하는 전국의 1차 의료기관 12개소 |
| **관찰연구 목적** | 실제 진료환경에서 2형 당뇨병 환자를 대상으로 24주간 엔블로정 또는 엔블로멧서방정 투여에 따른 체중 감소 효과 및 안전성의 확인 |
| **의뢰자** | ㈜대웅제약 |
| **대상질환** | 2형 당뇨병 |
| **선정·제외기준** | **선정기준**  아래의 선정기준을 모두 만족하는 자를 선정한다.   1. 만 19세 이상, 80세 이하의 성인 2. 2형 당뇨병 환자로 허가사항에 근거하여 연구자(담당의사)의 의학적 판단에 따라 엔블로정 또는 엔블로멧서방정을 최초로 투여할 예정인 자   - 이나보글리플로진 단독치료  - 이나보글리플로진 2제 병용 치료(메트포르민 병용)  - 이나보글리플로진 3제 병용 치료(메트포르민 + DPP4억제제 병용)   1. 2022 대한내분비학회 비만진료지침*에 따른 비만 전 단계 이상의 환자   * 대한내분비학회 비만진료지침  - 비만 전 단계 BMI 23~24.9 kg/m^2^  - 1단계 비만 BMI 25~29.9 kg/m^2^  - 2단계 비만 BMI 30~34.9 kg/m^2^  - 3단계 비만 BMI 35이상 kg/m^2^  4) 관찰연구 참여기간 동안 혈당조절을 위해 개별적으로 적절한 운동과 식사요법을 병행할 예정인 자  5) 관찰연구 참여기간 동안 적절한 피임법^*^에 따라 피임에 동의하거나, 임신할 계획이 없는 가임여성 및 남성  *호르몬성 피임제, 자궁 내 장치 또는 자궁 내 시스템의 이식, 정관수술, 난관 결찰, 이중 차단피임법(자궁 경부캡 또는 피임용 격막과 남성용 콘돔의 동시 사용 등) 등  6) 연구대상자가 본 관찰연구 및 연구대상의약품의 특성에 대하여 자세한 설명을 듣고 이해한 후, 자의로 관찰연구 참여와 연구기간 동안 대상자 주의사항을 준수하기로 서면 동의한 자  **제외기준**  아래의 제외기준 중 어느 하나라도 해당되는 자는 본 관찰연구에서 제외한다.     1. 2형 당뇨병 이외의 당뇨병(1형 당뇨병, 당뇨병성 케톤산증, 임신성 당뇨병 등)인 자 2. 엔블로정/엔블로멧서방정의 허가사항에 따라 투여 금기에 해당하는 자  - 엔블로정 또는 엔블로멧서방정의 구성성분에 과민반응 및 그 병력이 있는 환자 - 사구체여과율(eGFR, estimated Glomerular Filtration Rate) 30 mL/min/1.73m^2^ 미만인 환자 - 사구체여과율(eGFR, estimated Glomerular Filtration Rate) 60 mL/min/1.73m^2^ 미만의신장애 환자, 말기 신질환 또는 투석 중인 환자 - 중등증 및 중증의 간장애 환자(AST 또는 ALT > 정상 상한치의 3배, Total Bilirubin > 정상 상한치의 2배, 간염 또는 간부전) - 뉴욕심장학회(New York heart association, NYHA)의 분류에 의한 class III 혹은 IV인 자  1. 등록 시점 3개월 이내부터 비만치료제 또는 체중 감량 약물을 사용한 치료 또는 기타 치료(수술, 식이요법 등)으로 인해 체중이 불안정한 환자 2. 심신미약자 3. 임부 및 수유부 4. 다른 임상시험에 참여하여 임상시험용의약품 또는 임상시험용의료기기를 투여(적용)중인 자 5. 기타 연구자(담당의사)의 판단에 따라 본 관찰연구에 참여하는 것이 적합하지 않은 것으로 판단된 자 |
| **목표 연구대상자 수** | 약 240명 (통계학적 유의성 확보를 위한 최소 인원수에 중도 탈락자 약 15%를 고려) |
| **관찰 기간** | - 예상 전체 연구기간: IRB 승인일로부터 약 24개월   (단, 연구대상자 등록률에 따라 변경될 수 있음)   - 대상자별 관찰 기간: 약 24주(6개월) |
| **연구대상의약품** | 엔블로정 0.3 밀리그램, 엔블로멧서방정 0.3/1,000 밀리그램 |
| **관찰연구 설계 및 방법** | 본 관찰연구는 1차 의료기관의 실제 진료환경에서 엔블로정 또는 엔블로멧서방정을 투여 예정인 2형 당뇨병 환자를 대상으로 24주간 엔블로정/엔블로멧서방정 투여에 따른 체중 감소 효과 및 안전성을 확인하기 위한 전향적, 다기관 관찰연구로 설계되었다.  본 관찰연구는 실제 진료환경에서 허가사항(효능∙효과, 용법∙용량, 사용상의 주의사항 등)에 근거하여 연구자(담당의사)의 의학적 판단에 따라 엔블로정/엔블로멧서방정을 투여할 예정인 2형 당뇨병 환자를 모집단으로 계획하였다.  기존에 당뇨병 치료를 위한 약물요법 진행 여부와 관계없이 엔블로정/엔블로멧서방정 투여가 가능하다고 판단되는 모든 대상환자가 본 연구에 등록 가능하나, 엔블로정/엔블로멧서방정 투여가 결정되더라도 연구자의 의학적 판단에 따라 대상자의 자발적 연구 참여 동의가 없이는 관찰이 불가능하다.  본 관찰연구는 엔블로정/엔블로멧서방정 투여 여부와 무관하게 실제 진료환경에서 수행되는 인구학적 정보, 신체계측, 활력징후 등의 정보를 엔블로정/엔블로멧서방정 투여 후 최대 24주까지 수집한다. 자료는 실제 진료환경에서 기록되는 의무기록을 근거로 수집하며, 본 관찰연구에서 의무적으로 규정된 방문 및 검사 또는 처치는 없다.  그러나 진료환경에 따라 연구대상자 등록일(방문 1, 베이스라인, 0일)로부터 12주(±2주), 24주(±2주) 시점에 전향적으로 자료를 수집하는 추적조사가 진행 될 예정이며, 수집 항목은 연령, 성별, 과거병력 및 현재병력(고혈압, 이상지질혈증, 당뇨병, 심뇌혈관질환, 암 등), 생활습관 (흡연, 음주, 운동량), 선행 및 병용 약제(당뇨병, 고혈압, 이상지질혈증 치료제 등) 등의 기본 임상적 정보, 그리고 체중, 체성분 분석기를 통한 체성분 분석지표(체질량지수, 체지방량, 근육량 등), 공복혈당, 당화혈색소, 혈압 (수축기/이완기), 총콜레스테롤, LDL 콜레스테롤, HDL 콜레스테롤, 중성지방 등의 임상 지표, 안전성 평가 항목으로 활력징후, 검사실 검사, 이상 사례 등 이다. 이때 연구자는 일상 진료 과정에서 수집된 자료를 근거하여 연구에 필요한 자료를 수집한다.  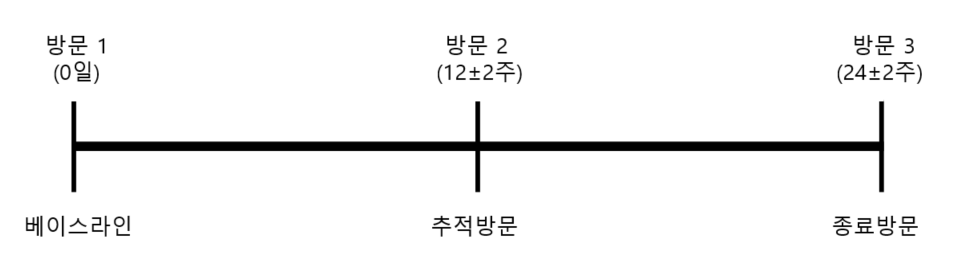  <관찰연구 흐름도> |
| **유효성 평가변수** | **1차 유효성 평가변수**   1. 베이스라인 대비 24주 시점의 체질량지수(BMI) 및 체중 변화   **2차 유효성 평가변수**   1. 베이스라인 대비 12주 시점의 체질량지수(BMI) 및 체중 변화 2. 베이스라인 대비 12주, 24주 시점의 5%이상 체질량지수(BMI) 및 체중 감소 대상자 비율 3. 베이스라인 대비 12주, 24주 시점의 체성분분석계 지표(체지방량, 내장지방량, 근육량, 허리/엉덩이 둘레 등) 변화량 4. 베이스라인 대비 12, 24주 시점의 HbA1c 변화율 및 변화량 5. 베이스라인 대비 12, 24주 시점의 FPG 변화율 및 변화량 6. 베이스라인 대비 12, 24주 시점의 혈압(수축기, 이완기) 변화량 7. 베이스라인 대비 12, 24주 시점의 HbA1c < 7 % 달성 대상자 비율 8. 베이스라인 대비 12, 24주 시점의 HbA1c < 6.5 % 달성 대상자 비율 9. 베이스라인 대비 12, 24주 시점의 치료적 반응[베이스라인 대비 각 평가 시점에서 HbA1c 변화량(베이스라인의 HbA1c–각 평가 시점의 HbA1c)> 0.5 % 또는 HbA1c < 7 %] 달성 대상자 비율   **탐색적 평가변수**   1. 베이스라인 대비 12, 24주 시점의 지질농도(Total cholesterol, LDL-C, HDL-C, Triglyceride)의 변화량 2. 베이스라인 대비 12, 24주 시점의 간기능 관련 지표(AST, ALT, γ-GTP)의 변화량 3. 베이스라인 대비 12, 24주 시점의 신기능 관련 지표[e-GFR, UACR(Urine Albumin to Creatinine Ratio), UGCR(Urine Glucose to Creatinine Ratio)] 변화량 4. 베이스라인 대비 12, 24주 시점의 체성분분석계 기타 지표(체수분, 세포내수분, 세포외수분, 세포외수분비, 복부지방률 등) 변화량 |
| **안전성 평가변수** | 1. 약물이상반응 (Adverse Drug Reaction, ADR)의 발현율 및 발현건수 2. 중대한 약물이상반응 (Serious ADR, SADR)의 발현율 및 발현건수 3. 주의 깊게 확인 할 이상사례^*^   * 저혈당증, 요로감염, 생식기 감염, 빈뇨, 다뇨 발생여부   1. 실험실적 검사, 활력징후, 신체검사 결과 |
| **통계분석 방법** | **일반적 원칙**  연속형 변수는 기술통계량(대상자의 수, 평균, 표준편차, 중앙값, 최소값, 최대값)을 제시하고, 범주형 변수는 빈도와 백분율을 제시한다. 특별한 명시가 없는 경우 모든 검정은 유의수준 5% 하에서 양측검정을 원칙으로 하며, 모든 p-value에 대해서는 소수점 최대 4자리까지 제시하고, 이외의 경우 소수점 두번째 자리까지 나누어 떨어지지 않을 경우, 소수점 세번째 자리에서 반올림하여 소수점 2자리까지 제시한다.  **유효성 평가변수**  1차 및 2차 유효성 평가변수에 대하여 베이스라인, 12주, 24주 시점에 기술통계량을 제시하고, 베이스라인 대비 24주 시점 혹은 12주 시점의 차이에 대하여 변화량에 대한 Paired t-test 또는 Wilcoxon signed rank test를 시행하여 분석한다.  **탐색적 평가변수**  탐색적 평가변수에 대하여 베이스라인, 12주, 24주 시점에 기술통계량을 제시하고, 베이스라인 대비 24주 시점 혹은 12주 시점의 차이에 대하여 변화량에 대한 Paired t-test 또는 Wilcoxon signed rank test를 시행하여 분석한다.  **안전성 평가변수**  연구기간 동안 수집된 이상사례∙약물이상반응, 중대한 이상사례∙약물이상반응, 예상하지 못한 이상사례∙약물이상반응, 예상하지 못한 중대한 이상사례∙약물이상반응에 대하여 이상사례 발현 대상자 수, 발현 건수, 발현율 및 양측 95 % 신뢰구간 등을 제시하며, 발현된 이상사례에 대하여 이상사례의 중증도, 연구대상의약품과의 인과관계, 연구대상의약품에 대한 조치, 연구대상의약품 외 조치, 이상사례 경과를 요약하여 제시한다.  활력징후에 대하여 베이스라인, 12주, 24주 시점에 기술 통계량을 제시하고 베이스라인 대비 24주 시점의 차이에 대하여 변화량에 대한 Paired t-test 또는 Wilcoxon signed rank test를 시행하여 분석한다.  검사실 검사에 대하여 베이스라인, 12주, 24주 시점에 기술 통계량을 제시하고 베이스라인 대비 24주 시점의 차이에 대하여 변화량에 대한 Paired t-test 또는 Wilcoxon signed rank test를 시행하여 분석한다.  또한, 정상(Normal or NCS), 임상적으로 의미 있는 비정상(CS) 변화를 빈도 및 백분율로 요약하여 제시한다. 또한, 임상적으로 의미 있는 비정상(CS) 항목에 대한 대상자의 상세정보를 목록으로 제시한다. |

**관찰연구 자료수집 일정표**

| **수집 일정**  **수집 항목** | **베이스라인** | **추적방문** | **종료방문** |
| --- | --- | --- | --- |
|  | 방문1 | 방문2 | 방문3 |
|  | 0일 | 12주(±2주) | 24주(±2주) |
| 서면동의 취득^1)^ | ○ |  |  |
| 등록번호 부여 | ○ |  |  |
| 선정/제외기준 확인 | ○ |  |  |
| 인구학적 정보^2)^ | ○ |  |  |
| 음주/흡연/생활습관^3)^ | ○ | ○ | ○ |
| 신체계측 및 체성분분석계(Inbody) 검사^4)^ | ○ | ○ | ○ |
| 활력징후^5)^ | ○ | ○ | ○ |
| 2형 당뇨병 정보^6)^ | ○ |  |  |
| 병력 조사^7)^ | ○ |  |  |
| 실험실 검사^8)^ | ○ | ○ | ○ |
| 연구대상의약품 투여 정보 | ○ | ○ | ○ |
| 선행/병용약물^9)^ | ○ | ○ | ○ |
| 이상사례 확인^10)^ |  | ○ | ○ |

*본 관찰연구를 위한 추가적인 방문 및 실시되는 검사실 검사는 없다. 일상적인 진료환경에서 방문1(베이스라인, 0일) 이후 24주까지 매 12주 마다(방문2: 12주 방문, 방문 3: 24주 방문) 평가하는 것이 권장되며, 방문 시 자료를 수집한다.

1. 관찰연구 절차를 수행하기 전에 서면동의서를 취득하여야 한다. 서면동의일과 방문1(베이스라인, 0일)은 상이할 수 있으나, 관찰연구 참여 전 반드시 동의 취득이 선행되어야 한다.
2. 인구학적 정보[이니셜, 성별, 연령(생년월), 임신 여부 및 수유 여부]를 수집한다.
3. 음주/흡연/생활습관은 매 방문 시 연구자(담당의사)의 문진으로 수집한다. 음주력은 주당 음주 횟수, 1회당 음주량에 대한 정보를 포함한다. 흡연력은 현재 흡연, 금연, 비흡연 중 한 가지를 선택한다. 생활습관은 식습관, 운동습관을 포함하며, 식습관 항목에는 불규칙한 식사, 과식, 탄수화물/당 과다섭취, 지방 과다섭취, 염분 과다섭취 항목 중 복수 선택 가능하며, 운동습관 항목에는 주당 운동횟수, 운동종류(걷기, 유산소운동, 근력운동), 운동강도(30분 이내, 1시간 이내, 장시간)를 포함한다.
4. 신체계측(신장) 및 체성분 분석계 측정 정보를 수집한다. 신장은 방문1(베이스라인, 0일) 시점에만 수집하며, 소수점 첫째 자리까지 수집한다. 체성분분석계(골격근량, 체지방량, 체지방률, 허리/엉덩이 둘레 등) 검사는 InBody770을 이용하여, 매 방문시 수집한다.
5. 최소 5분 이상의 휴식을 취한 후 활력징후(수축기 혈압, 이완기 혈압, 맥박) 정보를 수집한다. 매 방문마다 측정 검사 결과가 있는 경우, 결과를 수집한다.
6. 2형 당뇨병 정보(진단일)를 수집한다.
7. 병력 조사는 방문1(베이스라인, 0일)을 기준으로 6개월 이내의 과거병력과 등록 시점 현재 진행중인 현재병력을 수집한다.
8. 실험실 검사는 실제 진료환경에서 통상적인 진료 방법에 따라 진행한 경우 수집하며, 방문1(베이스라인, 0일) 기준으로 4주 이내 Hb1Ac 결과 및 그 외 3개월 이내 수행한 검사 결과가 있는 경우, 해당 검사 결과로 방문1(베이스라인, 0일) 검사실 검사를 대체할 수 있다. 검사실 검사 항목의 예시는 아래와 같다.

| 주요 검사실  검사 | HbA1c, FPG, Creatinine, eGFR, TC (Total Cholesterol), HDL, LDL, TG (Triglyceride), UACR, UGCR |
| --- | --- |
| 기타 검사실  검사  (혈액 검사) | RBC, Hemoglobin, Hematocrit, Platelet, WBC, Differential WBC count (Basophil, Eosinophil, Neutrophil, Lymphocyte, Monocyte), Na, K, Cl, P, Ca, Mg, BUN, LDH, Uric Acid, ALT, AST, ALP, γ-GTP, CPK, Total Bilirubin, Albumin, Total Protein, Glucose^*^, Insulin^*^ |
| 기타 검사실  검사  (요 검사) | pH, Specific Gravity, Bilirubin, Ketone, Nitrite, Protein (Albumin), RBC (Occult Blood), Urobilinogen, WBC (Leukocyte), Glucose, Creatinine |

* 계산식에 따라 HOMA-beta는 [360×공복 인슐린 농도(μU/mL)×공복 혈당(mg/dL)-63], HOMA-IR은 [공복 인슐린 농도(μU/mL)×공복 혈당(mg/dL)/405]을 적용하여 계산한다.

1. - 선행약물: 방문1(베이스라인, 0일) 기준 4주 이내에 2형 당뇨병 치료 목적의 당뇨병치료제에 한하여 약물명(성분명 또는 상품명), 투여 기간(투여 시작일, 투여 종료일, 지속 여부), 1회 투여 용량/단위, 1일 투여 횟수, 투여 경로 정보를 수집한다.

- 병용약물: 방문1(베이스라인, 0일) 이후 연구 기간 동안 3개월 이상 꾸준히 복용하는 2형 당뇨병 치료 목적 약물을 포함한 모든 병용약물(성분명 또는 상품명), 투여 사유, 투여 기간(투여 시작일, 투여 종료일, 지속 여부), 1회 투여 용량/단위, 1일 투여 횟수, 투여 경로, 투여 용량 변경 또는 투여 중단 여부 및 사유) 정보를 수집한다.

1. 이상사례는 연구대상의약품 투여 시점부터 종료 시점까지 발생한 이상사례에 대하여 수집한다. 방문3(종료방문, 24주) 시점에 발생하였거나, 해당 시점에 진행 중인 이상사례는 해당 이상사례가 소실될 때까지 또는 더 이상의 추적관찰이 의미 없는 것으로 연구자(담당의사)가 판단할 수 있는 시점까지 추적관찰하고, 마지막 연구대상자의 방문3(종료방문, 24주) 시점을 본 관찰연구를 완료한 것으로 간주한다. 또한, 24주 이내 중도 탈락한 대상자의 경우, 중도탈락 방문일로부터 30일까지 발현한 이상사례에 대하여 수집한다.

**목차**

[1. 서론 16](#_Toc157152867)

[1.1 대상질환에 대한 기술 16](#_Toc157152868)

[1.2 연구대상의약품에 대한 기술 및 이론적 근거 17](#_Toc157152869)

[2. 관찰연구의 목적 19](#_Toc157152870)

[2.1 1차 목적 19](#_Toc157152871)

[2.2 2차 목적 19](#_Toc157152872)

[2.3 탐색적 목적(탐색적 평가) 19](#_Toc157152873)

[2.4 안전성 평가 20](#_Toc157152874)

[3. 연구대상자의 선정 21](#_Toc157152875)

[3.1 연구 대상자 수 21](#_Toc157152876)

[3.2 산출근거 21](#_Toc157152877)

[3.3 선정기준 22](#_Toc157152878)

[3.4 제외기준 22](#_Toc157152879)

[3.5 관찰연구 완료 및 중지/탈락 23](#_Toc157152880)

[3.6 관찰연구의 중단 24](#_Toc157152881)

[4. 관찰연구 방법 25](#_Toc157152882)

[4.1 전반적인 관찰연구 디자인 25](#_Toc157152883)

[4.2 관찰연구 기간 26](#_Toc157152884)

[4.3 관찰연구의 진행 및 일정 26](#_Toc157152885)

[방문 일정 26](#_Toc157152886)

[4.4 수집정보 및 관찰항목 27](#_Toc157152887)

[관찰 항목 및 검사 방법 27](#_Toc157152888)

[5. 연구대상의약품 정보 31](#_Toc157152889)

[5.1 예상되는 이상사례(부작용) 및 사용상의 주의사항 32](#_Toc157152890)

[6. 이상사례 33](#_Toc157152891)

[6.1 이상사례의 정의 33](#_Toc157152892)

[6.2 이상사례의 평가 34](#_Toc157152893)

[6.3 이상사례의 보고 35](#_Toc157152894)

[6.4 중대한 이상사례의 보고 36](#_Toc157152895)

[6.5 임신 37](#_Toc157152896)

[7. 자료 관리 38](#_Toc157152897)

[7.1 근거 문서 38](#_Toc157152898)

[7.2 자료 입력 38](#_Toc157152899)

[7.3 자료 확인 39](#_Toc157152900)

[7.4 자료 보관 39](#_Toc157152901)

[7.5 자료 열람 39](#_Toc157152902)

[8. 평가기준 및 평가방법과 통계분석 방법 40](#_Toc157152903)

[8.1 평가변수 40](#_Toc157152904)

[8.2 통계분석 방법 41](#_Toc157152905)

[유효성 평가 대상자군 41](#_Toc157152906)

[안전성 평가 대상자군 41](#_Toc157152907)

[1차 유효성 평가변수 41](#_Toc157152908)

[2차 유효성 평가변수 41](#_Toc157152909)

[탐색적 평가변수 42](#_Toc157152910)

[이상사례 42](#_Toc157152911)

[활력징후 42](#_Toc157152912)

[실험실 검사 42](#_Toc157152913)

[하위집단 분석 42](#_Toc157152914)

[9. 윤리적 고려사항 및 행정적 절차 43](#_Toc157152915)

[9.1 연구자의 책임 43](#_Toc157152916)

[9.2 공용기관생명윤리위원회/ 임상시험심사위원회(IRB) 43](#_Toc157152917)

[9.3 윤리적 고려사항 43](#_Toc157152918)

[9.4 품질 보증 및 점검 43](#_Toc157152919)

[9.5 연구대상자 동의 44](#_Toc157152920)

[9.6 연구계획서 승인 및 변경 44](#_Toc157152921)

[9.7 임상연구 실시기관 모니터링 45](#_Toc157152922)

[9.8 기밀 유지 및 연구대상자의 비밀 보장 45](#_Toc157152923)

[9.9 연구대상자 안전보호에 관한 대책 45](#_Toc157152924)

[9.10 임상연구 결과의 이용 및 발표 46](#_Toc157152925)

[10. 연구 책임자 47](#_Toc157152926)

[10.1 연구 총괄 책임자 정보 47](#_Toc157152927)

[10.2 연구 총괄 책임자의 역할 및 책임 47](#_Toc157152928)

[11. 별첨 48](#_Toc157152929)

[12. 참고문헌 49](#_Toc157152930)

**약어 목록**

| ADR | Adverse Drug Reaction | 약물이상반응 |
| --- | --- | --- |
| AE | Adverse Event | 이상사례 |
| ALP | Alkaline Phosphatase | 알칼리인산분해효소 |
| ALT | Alanine Aminotransferase | 알라닌아미노전달효소 |
| AST | Aspartate Aminotransferase | 아스파르테이트아미노전달효소 |
| BMI | Body Mass Index | 체질량지수 |
| BUN | Blood Urea Nitrogen | 혈중요소질소 |
| CFR | Code of Federal Regulations | 미국연방정부규정 |
| CI | Chloride | 염소 |
| CPK | Creatine Phosphokinase | 크레아티닌포스포키나제 |
| CS | Clinically Significant | 임상적으로 의미 있는 비정상 |
| DPP-4 | dipeptidyl peptidase 4 | 다이펩타이드분해효소 |
| eCRF | electronic Case Report Form | 전자증례기록서 |
| EDC | Electronic Data Capture | 전자 데이터 수집 |
| eGFR | Estimated Glomerular Filtration Rate | 추정 사구체여과율 |
| FPG | Fasting Plasma Glucose | 공복혈당 |
| GCP | Good Clinical Practice | 의약품 임상시험 관리기준 |
| GLP-1 | Glucagon-like Peptide-1 | 글루카곤양 펩티드-1 |
| HbA1c | Hemoglobin A_1c_ | 헤모글로빈 A1c |
| HDL-C | High Density Lipoprotein Cholesterol | 고밀도 지질단백질 |
| ICH | International Council for Harmonisation | 국제의약품규제조화위원회 |
| IEC | Independent Ethics Committee | 독립된 윤리위원회 |
| IRB | Institutional Review Board | 기관생명윤리위원회 |
| LDL-C | Low Density Lipoprotein Cholesterol | 저밀도 지질단백질 |
| K | Potassium | 칼륨 |
| Mg | Magnesium | 마그네슘 |
| Na | Sodium | 나트륨 |
| NCS | Not Clinically Significant | 임상적으로 의미 없는 비정상 |
| NYHA | New York heart association | 뉴욕심장학회 |
| P | Phosphorus | 인 |
| PMS | Post Marketing Surveillance | 시판 후 조사 |
| RBC | Red Blood Cell | 적혈구 |
| SADR | Serious Adverse Drug Reaction | 중대한 약물이상반응 |
| SAE | Serious Adverse Event | 중대한 이상사례 |
| SE | Standard Error | 표준 오차 |
| SGLT-2 | Sodium-Glucose Cotransporter 2 | 나트륨-포도당 공동수송체 2 |
| SOP | Standard Operating Procedures | 표준작업지침서 |
| TC | Total Cholesterol | 총콜레스테롤 |
| TG | Triglyceride | 트리글리세리드 |
| UACR | Urine Albumin to Creatinine Ratio | 소변 알부민-크레아티닌 비율 |
| UGCR | Urine Glucose to Creatinine Ratio | 소변 포도당-크레아티닌 비율 |
| UGE | Urinary Glucose Excretion | 소변소 포도당 배출 |
| UKPDS | United Kingdom Prospective Diabetes Study | 영국 전향적 당뇨병 연구 |
| WBC | White Blood Cell | 백혈구 |
| γ-GTP | γ-Glutamyl Transpeptidase | 감마글루타밀트랜스펩티다제 |

# 서론

## 대상질환에 대한 기술

전 세계적으로 당뇨병 환자의 유병률과 사망률이 계속 증가하고 있다. 우리나라 30세 이상 성인의 당뇨병 유병률은 전체 인구 중2016년 기준으로 14.4 %로 약 501만 명으로 추정되며^1)^, 당뇨병은 우리나라 10대 사망원인 순위에서 2017년 6위를 차지하고 있다^2), 3)^.

2형 당뇨병 환자를 대상으로 시행한 전향적 코호트 연구 United Kingdom Prospective Diabetes Study (UKPDS)에 의하면 2형 당뇨병 환자에서 당화혈색소(HbA1c)를 1 % 줄이면 미세혈관합병증 위험을 37 %, 심근경색 위험을 14 % 줄일 수 있다^4)^. 이후 여러 연구를 통해 2형 당뇨병 환자에서 적극적인 혈당조절은 당뇨병성 합병증 발생을 예방할 수 있으며, 이미 발생된 합병증의 진행속도를 늦출 수 있음이 방법으로 확인되었다^3), 5)^.

2형 당뇨병 진단과 동시에 모든 환자에게 생활습관개선을 위한 교육을 실시하고 실천하도록 권고한다. 하지만, 대부분의 2형 당뇨병 환자는 생활습관개선만으로 목표 수준의 혈당에 도달하기 어렵기 때문에 적극적인 생활습관개선과 더불어 적절한 당뇨병 치료약제를 사용이 필요하다^6)^.

2형 당뇨병 환자 치료를 위한 경구혈당강하제는 작용기전에 따라 신장 근위세뇨관에서 당 재흡수를 억제하는 Sodium-Glucose Cotransporter 2 (SGLT-2) 억제제, 간에서 포도당합성을 억제하는 비구아나이드계의 메트포르민 및 인크레틴 효과를 증강시키는 Dipeptidyl Peptidase 4 (DPP-4) 억제제 등이 있다^6)^. 2021년 대한당뇨병학회 당뇨병 진료지침에 의하면, 특히 죽상경화심혈관질환을 동반한 경우 병용요법 시 심혈관이익이 입증된 SGLT-2 억제제 혹은 GLP-1 (Glucagon-like Peptide-1) 수용체작용제를 포함한 치료를 우선 고려하며, 알부민뇨가 있거나 추정사구체여과율이 감소한 경우에도 심혈관 및 신장이익이 입증된 SGLT-2 억제제를 포함한 치료를 우선 고려할 것을 권고한다^7)^.

이러한 진료 지침에 따라 최근 SGLT-2억제제의 사용이 증가하고 있다. SGLT-2 억제제는 요중 포도당 배설의 증가를 통해 인슐린 분비의 상대적 감소를 초래하고, 글루카곤/인슐린 비를 증가시킨다. 이에 따라 지방 분해를 촉진하고 케톤체의 생성이 증가하게 된다. 당뇨병환자에서 혈중 케톤체가 심근의 효과적인 에너지원으로 사용되어 심장기능 향상에 기여한다는 “Super-fuel” 가설이 제시된 바가 있다^8), 9)^. 이러한 대사적 이득은 이 약제가 단순히 당뇨병 치료뿐 아니라 비만, 고혈압, 이상지질혈증 등 다양한 대사적 위험인자에 폭넓은 유익을 제공할 수 있음을 시사한다.

특히 SGLT-2 억제제 사용으로 소변으로 배출되는 포도당은 매일 60~80 g으로 추산되는데, 이는 매일 30분 달리기하는 정도의 칼로리인 230~310 kcal의 에너지 소모와 비견된다^10)^. 따라서 체중에 변화가 없는 당뇨병 환자에게 SGLT-2 억제제를 사용하면 체중 감량과 관련된 대사적 이득을 기대할 수 있다. SGLT-2 억제제 사용 초기의 체중 감소는 대부분 간의 글리코겐 감소와 탈수 효과로 인한다. 하지만 장기적으로 지방산 산화의 증가, 지방 세포에서의 유리지방산 배출 증가에 따른 내장지방 및 피하지방량 감소가 관여하는 것으로 알려져 있다^11)^. 기존 문헌에 의하면 SGLT-2 억제제 사용 첫 6개월간 2~3 kg 정도의 체중 감소가 일어나는 것으로 알려져 있다^11)^.

## 연구대상의약품에 대한 기술 및 이론적 근거

엔블로정은 ㈜대웅제약에서 국내 최초로 개발한 SGLT-2 억제제로 2022년11월 30일 식품의약품안전처에서 2형 당뇨병의 환자의 혈당조절을 향상시키기 위한 식사요법 및 운동요법의 보조적 목적으로 허가를 받아 시판되었다. 허가 용법∙용량 및 권장 용량은 단독요법 및 다른 혈당 강하제와의 추가 병용요법을 위해 1일 1회 0.3 mg이며, 식사와 관계없이 투약할 수 있다.

임상 연구 결과 엔블로정은 단회투여(0.2~5 mg) 및 15 일 반복투여(0.1~2 mg) 시 안전하고 우수한 내약성을 확인했고, 강력하고 오래 지속되는 UGE (Urinary Glucose Excretion)를 확인했다. 또한, 모든 용량(0.1 mg, 0.3 mg, 0.5 mg)에서 당화혈색소 및 공복 혈장포도당 강하에 효과적이었으며, 12주 동안 1일1회 투여 시 유리한 안전성 프로파일을 보였다. 추가적으로, 단독요법 또는 Metformin, Gemigliptin과의 병용요법에 대한 유효성 및 안전성 프로파일을 확인했다^12)^. 이외 다수의 선행연구에서 SGLT-2 억제제는 저혈당을 거의 일으키지 않으면서 혈당을 감소시키는 작용 외에도, 체중 또는 혈압 감소에 대한 긍정적 효과가 보고되었다^13)-15)^.

심혈관 질환은 당뇨병 환자의 주요 사망 원인 중 하나로, 이는 당뇨병의 유병 기간에 따라 증가한다. 고혈압, 이상지질혈증, 대사증후군, 만성 신장 질환과 같은 동반 질환 유무에 영향을 받으며, 이러한 심혈관 질환은 당뇨병 환자의 삶의 질을 저하시키고, 일상생활의 제약과 부작용으로 인해 지속적인 치료와 관리를 필요로 한다^16)^. 따라서, 당뇨병 환자에서 심혈관 질환의 예방과 치료는 중요한 요소 중 하나로 간주된다. 미국식품의약국(Food and Drug Administration, FDA)은 2008년부터 모든 당뇨병 치료 약제의 승인을 위해 심혈관 질환 발생에 대한 안전성 자료를 요구했다^17), 18)^. 이에 따라 최근 당뇨병의 치료적 목표는 심혈관 질환의 위험도 경감을 상대적으로 중시하여, 당뇨병 치료 약제의 선택에 있어 심혈관 안정성 및 유효성의 증거가 있는 약제의 사용을 우선 추천하는 방향으로 치료 가이드라인이 변화하고 있다^19)^.

하지만 실제 진료 환경에서 효과 또는 안전성을 평가하는 자료는 부족한 실정이며, 일반적인 시판 후 조사(Post Marketing Surveillance, PMS)는 장기적인 안전성의 확인을 주요 목표로 하기 때문에 임상시험용 의약품의 효과를 평가하는데 한계가 있다. 따라서 실제 진료환경에서 엔블로정의 치료 효과와 안전성을 관찰, 비교 또는 평가하기 위한 연구가 필요한 상황이다. 특히 본 연구는 엔블로정의 혈당강하 효과 외에도 체중, 체지방 등 다양한 대사적 지표에 대한 국내 사용자 중심의 실사용근거(Real World Evidence, RWE) 확보를 위해 학술 목적으로 고안되었다.

# 관찰연구의 목적

본 관찰연구는 실제 진료환경에서 엔블로정 혹은 엔블로멧서방정 투여 예정인 2형 당뇨병 환자를 대상으로 24주간 엔블로정/엔블로멧서방정 투여에 따른 체중 감소 효과와 안전성의 확인을 목적으로 한다.

## 1차 목적

베이스라인 대비 24주 시점의 체질량지수 및 체중의 변화에 대해 평가한다.

## 2차 목적

엔블로정 또는 엔블로멧서방정 투여의 아래 효과를 평가한다.

1. 베이스라인 대비 12주 시점의 체질량지수 및 체중 변화
2. 베이스라인 대비 12, 24주 시점의 5%이상 체질량지수 및 체중 감소 대상자 비율
3. 베이스라인 대비 12, 24주 시점의 체성분분석계 지표(체지방량, 내장지방량, 근육량, 허리/엉덩이 둘레 등) 변화량
4. 베이스라인 대비 12, 24주 시점의 HbA1c 변화량
5. 베이스라인 대비 12, 24주 시점의 FPG 변화량
6. 베이스라인 대비 12, 24주 시점의 혈압(수축기, 이완기) 변화량
7. 베이스라인 대비 12, 24주 시점의 HbA1c <7% 달성 대상자 비율
8. 베이스라인 대비 12, 24주 시점의 HbA1c <6.5% 달성 대상자 비율
9. 베이스라인 대비 12, 24주 시점의 치료적 반응[베이스라인 대비 각 평가 시점에서 HbA1c 변화량(베이스라인의 HbA1c–각 평가 시점의 HbA1c)> 0.5 % 또는 HbA1c < 7 %] 달성 대상자 비율

## 탐색적 목적(탐색적 평가)

엔블로정 또는 엔블로멧서방정 투여의 아래 효과를 탐색적으로 확인한다.

1. 베이스라인 이후 12, 24주 시점의 지질농도(Total cholesterol, LDL-C, HDL-C, Triglyceride)의 변화량
2. 베이스라인 이후 12, 24주 시점의 간기능 관련 지표(AST, ALT, γ-GTP)의 변화량
3. 베이스라인 이후 12, 24주 시점의 신기능 관련 지표[e-CFR, UACR(Urine Albumin to Creatinine Ratio), UGCR(Urine Glucose to Creatinine Ratio)] 변화량
4. 베이스라인 이후 12, 24주 시점의 체성분분석계 기타 지표(체수분, 세포내수분, 세포외수분, 세포외수분비, 복부지방률 등) 변화량

## 안전성 평가

엔블로정 또는 엔블로멧서방정 투여의 아래 안전성을 확인한다.

1. 약물이상반응(Adverse Dug Reaction, ADR)의 발현율 및 발현건수
2. 중대한 약물이상반응(Serious ADR, SADR)의 발현율 및 발현건수
3. 주의 깊게 확인할 이상사례^*^

^*^ 저혈당증, 요로감염, 생식기 감염, 빈뇨, 다뇨 발생여부

1. 실험실적 검사, 활력징후, 신체검사 결과

# 연구대상자의 선정

## 연구 대상자 수

최소 240명 (통계적 유의성 확보를 위한 최소 인원수, 중도탈락 약 15% 고려)

## 산출근거

본 연구는 2형 당뇨병 환자를 대상으로 24주간 엔블로정/엔블로멧서방정의 투여에 따른 체중 감량 효과를 포함한 유효성 및 안전성을 관찰하기 위한 대규모, 전향적, 다기관, 비중재적 데이터베이스 관찰연구이다.

본 연구는 단일군으로 진행되는 관찰연구로, 기 진행된 2상 및 3상 임상시험^18)^ 연구의 체중 및 체질량지수의 베이스라인 대비 12주 또는24주 시점의 변화량에 대한 평균과 표준편차를 참고하여 G*Power 3.1.9.7을 이용해 산출하였다 (그림 2).

[Assumptions]

- Effect size: 0.25 (Mean of difference 2.5, SD of difference 10.0)
- Power: 0.95
- α error: 0.05
- Drop-out rate: 30%


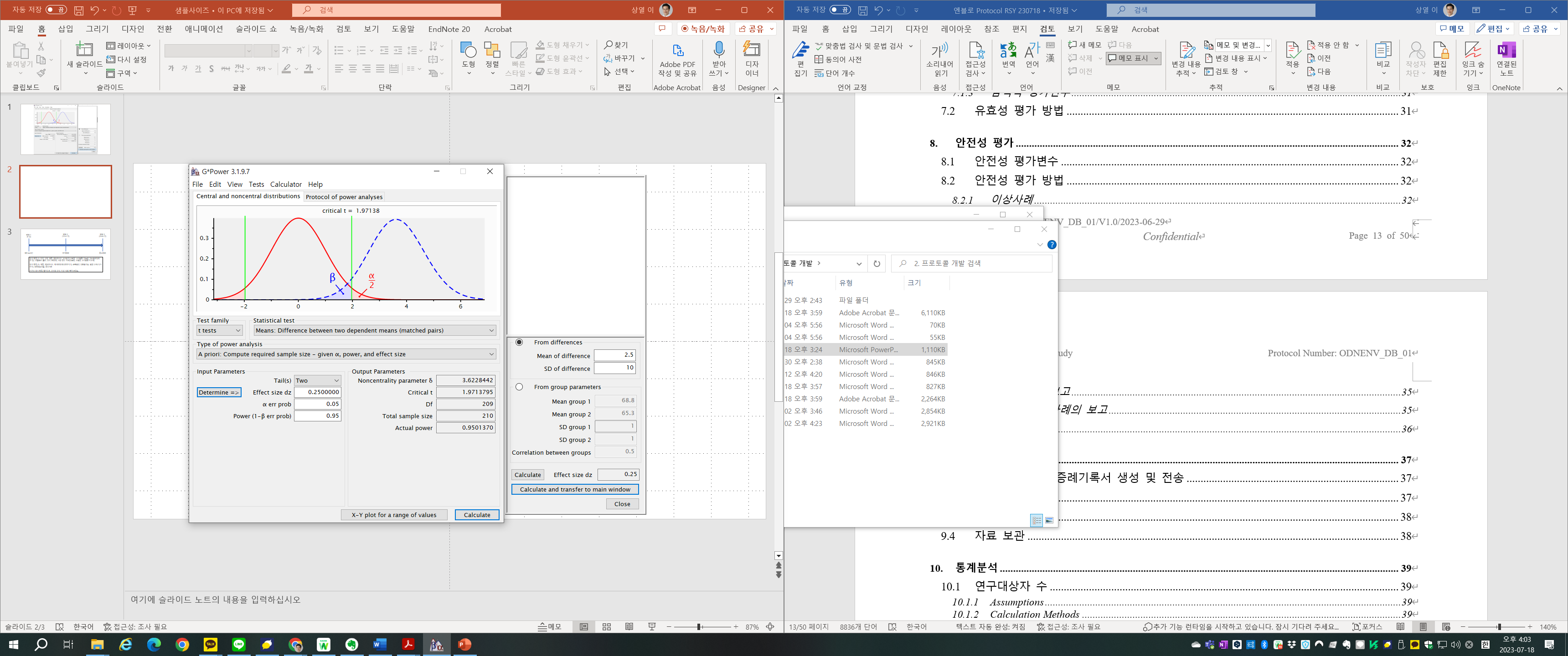


그림 2. 샘플사이즈 계산 (G*Power)

이전 연구를 참조하여 엔블로정 또는 엔블로멧 서방정의 중재로 인한 평균 체중의 변화를 2.5 kg, 표준편차를 10.0으로 할 때 effect size는 0.25가 되며, Power: 0.95, α error: 0.05로 설정할 때 필요한 총 샘플 사이즈는 약 210명으로 추산되어, Drop-out rate를 약 15%로 상정하여 약 240명의 연구대상자를 모집하고자 한다.

## 선정기준

아래의 선정기준을 모두 만족하는 자를 선정한다.

1. 만 19세 이상, 80세 이하의 성인 남∙녀
2. 2형 당뇨병 환자로 허가사항에 근거하여 연구자(담당의사)의 의학적 판단에 따라 엔블로정 혹은 엔블로멧서방정을 최초로 투여할 예정인 자

- 이나보글리플로진 단독치료

- 이나보글리플로진 2제 병용 치료(메트포르민 병용)

- 이나보글리플로진 3제 병용 치료(메트포르민 + DPP4억제제 병용)

1. 2022 대한내분비학회 비만진료지침*에 따른 비만 전 단계 이상의 환자

* 대한내분비학회 비만진료지침

- 비만 전 단계 BMI 23~24.9 kg/m^2^

- 1단계 비만 BMI 25~29.9 kg/m^2^

- 2단계 비만 BMI 30~34.9 kg/m^2^

- 3단계 비만 BMI 35이상 kg/m^2^

4) 관찰연구 참여기간 동안 혈당조절을 위해 개별적으로 적절한 운동과 식사요법을 병행할 예정인 자

5) 관찰연구 참여기간 동안 적절한 피임법*에 따라 피임에 동의하거나, 임신할 계획이 없는 가임여성 및 남성

*호르몬성 피임제, 자궁 내 장치 또는 자궁 내 시스템의 이식, 정관수술, 난관 결찰, 이중 차단피임법(자궁 경부캡 또는 피임용 격막과 남성용 콘돔의 동시 사용 등) 등

6) 연구대상자가 본 관찰연구 및 연구대상의약품의 특성에 대하여 자세한 설명을 듣고 이해한 후, 자의로 관찰연구 참여와 연구기간 동안 대상자 주의사항을 준수하기로 서면 동의한 자

## 제외기준

아래의 제외기준 중 어느 하나라도 해당되는 자는 본 관찰연구에서 제외한다.

1) 2형 당뇨병 이외의 당뇨병(1형 당뇨병, 당뇨병성 케톤산증, 임신성 당뇨병 등)인 자

2) 엔블로정/엔블로멧서방정의 허가사항에 따라 투여 금기에 해당하는 자

- 엔블로정 또는 엔블로멧서방정의 구성성분에 과민반응 및 그 병력이 있는 환자

- 사구체여과율(eGFR, estimated Glomerular Filtration Rate) 30 mL/min/1.73m^2^ 미만인 환자

- 사구체여과율(eGFR, estimated Glomerular Filtration Rate) 60 mL/min/1.73m2 미만의, 신장애 환자, 말기 신질환 또는 투석 중인 환자

- 중등증 및 중증의 간장애 환자(AST 또는 ALT > 정상 상한치의 3배, Total Bilirubin > 정상 상한치의 2배, 간염 또는 간부전)

- 뉴욕심장학회(New York heart association, NYHA)의 분류에 의한 class III 혹은 IV인 자

3) 등록 시점 3개월 이내부터 비만치료제 또는 체중 감량 약물을 사용한 치료 또는 기타 치료(수술, 식이요법 등)으로 인해 체중이 불안정한 환자

4) 심신 미약자

5) 임부 및 수유부

6) 다른 임상시험에 참여하여 임상시험용의약품 또는 임상시험용의료기기를 투여(적용)중인 자

7) 기타 연구자(담당의사)의 판단에 따라 본 관찰연구에 참여하는 것이 적합하지 않은 것으로 판단된 자

## 관찰연구 완료 및 중지/탈락

1. **관찰연구 완료 및 연구 종료**

연구대상자의 최초방문(Baseline) 및 연구대상의약품 투여 후 24주 시점의 종료방문 절차가 완료되면 해당 연구대상자는 관찰연구를 완료한 것으로 간주한다. 본 관찰연구에서는 마지막 연구대상자의 관찰연구 종료를 전체 관찰연구의 종료로 정의한다.

1. **관찰연구 중지/탈락**

연구기간 동안 연구대상자는 본인의 의지로 언제든지 임상연구를 중단할 수 있으며, 안전, 행위 또는 행정상의 이유로 연구자 또는 의뢰자의 재량에 의해 언제든지 중지/ 탈락할 수 있다. 만일 이상사례(Adverse Event, AE)로 인하여 중지/탈락한 경우, 해당 연구대상자의 이상사례 경과를 추적 관찰하도록 최대한 노력해야 한다. 임상연구에서 연구대상자가 중지/탈락 될 수 있는 경우는 다음과 같으며, 연구자는 연구대상자의 중지/탈락하는 경우, 해당 시점까지 수집된 연구자료를 eCRF에 작성한다.

연구대상자의 관찰연구 참여를 중단하고 중도탈락 시킬 수 있는 경우는 다음과 같다.

1. 관찰연구 진행 도중 연구대상자의 선정/제외기준 위반을 발견한 경우(Not meet the inclusion/exclusion criteria)
2. 이상사례(Adverse Event)

: 연구자 판단 결과 이상사례로 관찰연구를 수행하기 어려운 경우

1. 연구대상자 또는 연구대상자 대리인의 동의철회[Withdrawal by Subject (or his/her legal representative)]
2. 연구대상의약품 비순응(Non-Compliance with Investigational Product)

: 연구대상자가 연구대상의약품과 관련된 연구자의 지시에 동의하지 않거나 따르지 않은 경우

1. 추적불능(Lost to follow-up)

: 연구대상자의 추적이 불가능한 경우

1. 연구자 판단(Physician Decision)

연구대상자의 중지/탈락이 발생하는 경우, 연구자는 의뢰자에게 통보하고 관찰연구 종료 평가를 완료하도록 최선을 다해야 한다. 중도탈락의 사유를 전자증례기록서(electronic Case Report Form, eCRF) 및 연구대상자의 근거문서에 기록한다. 탈락한 연구대상자는 재참여가 불가능하다.

## 관찰연구의 중단

연구책임자, 연구자(IRB 승인 및 연구책임자가 위임한 연구담당자)는 관찰연구 과정에서 관찰된 결과에 비추어 연구를 지속하는 것이 바람직하지 않다고 판단될 경우, 연구 지원기관(의뢰자)와 협의하여 관찰연구를 조기 중단 또는 일시 중지 할 수 있으며, 의뢰자는 안전성 혹은 관리상의 이유로 관찰연구를 조기중단 또는 일시 중지 시킬 수 있다. 연구책임자는 이 사실을 IRB에 즉시 보고하고, 조기중단 또는 일시 중지에 대한 상세한 사유를 제출 해야 한다.

의뢰자에 의한 연구중단 사유는 다음과 같다.

1. 본 연구대상의약품의 품목 승인이 취소되거나 판매를 중지하는 경우
2. 의뢰자의 판단 상 본 연구의 진행이 의학적 또는 윤리적으로 정당화되지 않는다고 판단한 경우.
3. 연구대상자 모집이 지나치게 지체되어 연구의 진행이 어렵다고 판단되는 경우 등

어떠한 이유로든 연구가 조기 중단 또는 일시 중지된 경우 연구자는 연구대상자에게 이 사실을 알리고 적절한 조치와 추적관찰이 이루어질 수 있도록 한다. 연구자는 중단된 시점까지 진행된 연구대상자에 대한 eCRF, 연구 진행현황 및 결과를 정리하여 의뢰자에게 전달한다.

# 관찰연구 방법

## 전반적인 관찰연구 디자인

본 관찰연구는 1차 의료기관의 실제 진료환경에서 엔블로정 또는 엔블로멧서방정 투여 예정인 2형 당뇨병 환자를 대상으로 24주간 엔블로정/엔블로멧서방정 투여에 따른 체중 감소 효과 및 안전성을 확인하기 위한 전향적, 다기관 관찰연구로 설계하였다.

본 관찰연구는 실제 진료환경에서 허가사항(효능∙효과, 용법∙용량, 사용상의 주의사항 등)에 근거하여 연구자(담당의사)의 의학적 판단에 따라 엔블로정/엔블로멧서방정을 투여할 예정인 2형 당뇨병 환자를 모집단으로 계획하였다.

기존에 당뇨병 치료를 위한 약물요법 진행 여부와 관계없이 엔블로정/엔블로멧서방정 투여가 가능하다고 판단되는 모든 대상환자가 본 연구에 등록 가능하나, 엔블로정/엔블로멧서방정 투여가 결정되더라도 연구자의 의학적 판단에 따라 대상자의 자발적 연구 참여 동의가 없이는 관찰이 불가능하다.

본 관찰연구는 엔블로정/엔블로멧서방정 투여 여부와 무관하게 실제 진료환경에서 수행되는 인구학적 정보, 신체계측, 활력징후 등의 정보를 엔블로정/엔블로멧서방정 투여 후 최대 24주까지 수집한다. 자료는 실제 진료환경에서 기록되는 의무기록을 근거로 수집하며, 본 관찰연구에서 의무적으로 규정된 방문 및 검사 또는 처치는 없다.

그러나 진료환경에 따라 연구대상자 등록일(방문 1, 베이스라인, 0일)로부터 12주(±2주), 24주(±2주) 시점에 전향적으로 자료를 수집하는 추적조사가 진행 될 예정이며, 수집 항목은 연령, 성별, 과거병력 및 현재병력(고혈압, 이상지질혈증, 당뇨병, 심뇌혈관질환, 암 등), 생활습관(흡연, 금주, 운동량), 선행 및 병용 약제(당뇨병, 고혈압, 이상지질혈증 치료제 등) 등의 기본 임상적 정보, 그리고 체중, 체성분 분석기를 통한 체성분 분석지표(체질량지수, 체지방량, 근육량 증), 공복혈당, 당화혈색소, 혈압(수축기/이완기), 총콜레스테롤, LDL콜레스테롤, HDL콜레스테롤, 중성지방 등의 임상 지표, 안전성 평가 항목으로 활력징후, 검사실 검사, 이상사례 등이다. 이때 연구자는 일상 진료 과정에서 수집된 자료를 근거하여 연구에 필요한 자료를 수집한다.


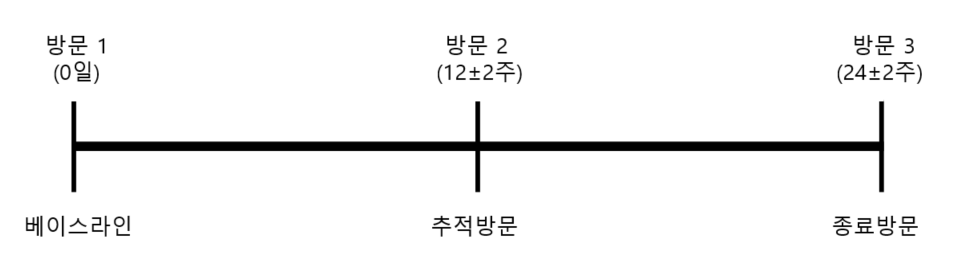


그림 1. 관찰연구 흐름도

## 관찰연구 기간

본 연구 기간은 IRB승인일로부터 약 24개월간 진행될 예정이다. 각 연구대상자 별 자료수집 기간은 최대 24주(6개월)로, 해당 기간 동안 연구계획서에 명시된 정보를 수집한다. 단, 연구대상자 선정의 어려움 등으로 인해 연구 진행에 영향을 미칠 수 있는 상황이 발생한 경우에는 기간의 변동이 있을 수 있다.

## 관찰연구의 진행 및 일정

### 방문 일정

본 연구에서 의무사항으로 규정된 방문, 검사, 실험실적 검사 또는 처치는 없다. 통상적인 진료방문 시 진료 기록, 연구자 평가, 실험실 검사치 등을 이용하여 전향적으로 수집할 것이다. 각 연구자들은 베이스라인과 추적관찰 기간 동안 연구대상자들에 대해 사전에 규정된 정보를 수집할 것이다.

1. **방문1(베이스라인, 0일)**

최초방문(베이스라인)에 아래 각 항목에 대한 정보를 수집한다.

- 연구대상자 동의 취득 및 연구대상자 등록 번호 부여
- 선정/제외기준 확인
- 인구학적 정보 (이니셜, 성별, 연령(생년월), 임신 여부 및 수유 여부)
- 음주/흡연/생활습관 정보
- 신체계측 (신장, 체중)
- 체성분 분석계 검사 (체질량지수, 골격근량, 체지방량, 체지방률 등 포함)
- 활력징후 (수축기/이완기 혈압, 맥박)
- 2형 당뇨병 정보 (진단일)
- 병력 정보 (대상 질환을 제외한 등록 전 6개월 이내의 과거병력 및 현재 병력)
- 선행 (등록 전 4주 이내 당뇨병치료제)약물/ 병용약물(등록 시점 복용중인 당뇨병치료제를 포함한 모든 병용 투여 약물)
- 실험실 검사 (HbA1c, FPG, Total Cholesterol, LDL-C, HDL-C, Triglyceride, ALT, AST, Creatinine, e GFR, UACR, UGCR 등)
- 엔블로정 또는 엔블로멧서방정 투여 정보

1. **추적방문**

추적방문은 최초방문(베이스라인)으로부터 12주 간격으로 방문2(12주±2주), 방문3(24주±2주)을 실시할 수 있다. 추적 방문 시 아래 각 항목에 대한 정보를 수집한다.

- 음주/흡연/생활습관 정보
- 신체계측 (체중)
- 활력징후 (수축기/이완기 혈압, 맥박)
- 체성분 분석계 검사 (체질량지수, 골격근량, 체지방량, 체지방률 등 포함)
- 병용약물
- 실험실 적 검사 (HbA1c, FPG, Total Cholesterol, LDL-C, HDL-C, Triglyceride, ALT, AST, Creatinine, e GFR, UACR, UGCR 등)
- 엔블로정 또는 엔블로멧서방정 투여 정보
- 이상사례

## 수집정보 및 관찰항목

### 관찰 항목 및 검사 방법

1. **서면동의 취득 및 연구 대상자 등록 번호 부여**

연구자는 본 연구와 관련된 자료를 수집하기 전 연구대상자에게 대상자 설명문을 통해 본 연구의 목적과 내용에 대하여 상세히 설명하고, 연구대상자의 자발적인 동의를 얻고 연구대상자의 성명과 서명, 서명일이 포함된 동의서를 득한다.

서면동의일과 방문1(베이스라인, 0일)은 상이할 수 있으나, 임상연구 참여 전 반드시 동의 취득이 선행되어야 한다.

대상자의 등록번호는 연구대상자 동의 취득 후 e CRF에 대상자 등록 시 자동으로 부여되며, 대상자 번호는 ‘AXX-ZZZ’ 형식의 총 6자리로 다음과 같이 구성된다.

- ‘A’: 분류기호
- ‘XX’: 연구기관 번호 (001, 002 ~)
- ‘ZZZ’: 각 기관별 동의한 연구대상자 번호 (001, 002 ~)

1. **선정/제외기준 확인**

선정/제외기준 확인은 방문1(베이스라인, 0일) 시점에 선정기준을 모두 만족하는지 여부, 제외기준 중 어느 하나라도 해당되는지 여부를 확인한다.

1. **인구학적 정보**

방문1(베이스라인, 0일) 시점에 대상자의 기초정보를 확인하기 위하여 다음의 항목을 수집한다. 임신 여부는 문진 또는 임신검사 결과가 있는 경우 수집한다.

- 이니셜
- 성별
- 생년월, 연령(만 나이)
- 임신 및 수유 여부

1. **2형 당뇨병 정보**

방문1(베이스라인, 0일) 시점에 엔블로정 또는 엔블로멧서방정의 투여 목적을 확인하기 위하여 2형 당뇨병의 진단일을 수집한다.

1. **병력 조사**

병력의 경우, 방문1(베이스라인, 0일) 전까지 관찰된 대상질환을 제외한 임상적으로 유의한 의학적 상태 또는 비정상으로 정의하며, 방문1(베이스라인, 0일) 전 6개월 이내의 병력 및 현재 진행중인 병력에 대하여 각 병력의 진단명 및 진단일을 수집한다.

1. **음주/흡연/생활습관 정보**

방문1(베이스라인, 0일) 시점에 대상자의 기초정보를 확인하기 위하여 다음의 항목을 수집하고, 이후 모든 방문 시점에 변화된 사항이 있는 경우 추가적으로 수집한다.

1. 음주력
   - 현재 음주^*^: 평생 12잔(Units) 이상 음주하였고, 12개월 이내에 1회 이상 음주
   - 과거 음주: 평생 12잔(Units) 이상 음주하였으나, 12개월 이내 음주하지 않음
   - 비음주: 평생 12잔(Units) 미만으로 음주

^*^ 현재 음주자의 경우, 주당 음주 횟수, 1회당 음주량을 확인한다.

1. 흡연력
   - 현재 흡연: 평생 담배 5갑(100개피) 이상 피웠고, 30일 이내 흡연
   - 과거 흡연: 평생 담배 5갑(100개피) 이상 피웠고, 30일 이내 흡연하지 않음
   - 비흡연: 평생 담배 5갑(100개피) 미만으로 흡연
2. 생활습관(식습관): 불규칙한 식사, 과식, 탄수화물/당 과다섭취 여부, 지방 과다섭취 여부, 염분 과다섭취 여부
3. 생활습관(운동습관): 운동종류(걷기, 유산소운동, 근력운동), 주당 운동 횟수, 운동 강도(30분 이내, 1시간 이내, 장시간)
4. **신체계측**

모든 방문시점에 체중을 수집하며, 신장은 방문1(베이스라인, 0일) 시점 1회 수집한다.

1. **활력징후**

모든 방문시점에 활력징후로 아래의 항목을 수집한다.

- 혈압(수축기/이완기)
- 맥박

혈압 및 맥박은 측정 전 최소 5분 동안 휴식을 취한 후 조용한 환경에서 등받이가 있는 의자에 앉아 측정하는 것을 권장하며, 가능한 측정 전 30분 이내에는 흡연, 알코올, 카페인 섭취를 하지 않도록 한다. 이상반응 정의에 부합되는 임상적으로 유의한 비정상 및 결과는 이상사례로 수집한다.

1. **체성분분석계 검사**

체성분분석계 검사는 InBody770을 이용하여 측정하며, 측정이 가능한 기관에 한하여 방문1(베이스라인, 0일), 방문2(추적방문, 12주) 및 방문 3(종료방문, 24주) 시점에 아래의 항목이 포함될 수 있도록 최대한 수집한다.

- 체질량지수
- 골격근량
- 체지방량
- 체지방률
- 허리/엉덩이 둘레
- 기타 지표(체수분, 세포내수분, 세포외수분, 세포외수분비, 복부지방률 등)

1. **선행/병용약물**

선행약물의 경우 방문1(베이스라인, 0일) 기준 4주 이내에 투여한 당뇨병치료제에 한하여 수집하며, 병용약물은 방문1(베이스라인, 0일) 시점에 3개월 이상 꾸준히 복용한 약물에 대한 투여 정보를 수집한다.

- 약물명(상품명)
- 용법·용량 (1회 투여 용량, 단위, 투여 빈도, 투여 경로)
- 투여기간(투여시작일, 투여 종료일, 지속여부)
- 투여 목적
- 용량 변경 또는 투여 중단된 경우 용량 변경 또는 투여 중단 사유

1. **실험실 검사**

실험실 검사는 실제 진료환경에서 통상적인 진료 방법에 따라 검사를 수행한 경우 의무기록을 토대로 수집하며, 방문1(베이스라인, 0일) 기준으로 4주 이내 수집된 HbA1c 결과 및 그 외 3개월 이내 수집된 검사 결과가 있는 경우, 해당 검사 결과로 방문1(베이스라인, 0일) 실험실 검사를 대체할 수 있다.

HOMA-beta는 [360×공복 인슐린 농도(μU/mL)×공복 혈당(mg/dL)-63], HOMA-IR은 [공복 인슐린 농도(μU/mL)×공복 혈당(mg/dL)/405]을 적용하여 계산식에 따라 계산한다.

수집될 수 있는 실험실 검사 항목 예시는 아래와 같다.

| 주요 검사실 검사 | HbA1c, FPG, Creatinine, eGFR, TC (Total Cholesterol), HDL, LDL, TG (Triglyceride), UACR, UGCR |
| --- | --- |
| 기타 검사실 검사  (혈액 검사) | RBC, Hemoglobin, Hematocrit, Platelet, WBC, Differential WBC count (Basophil, Eosinophil, Neutrophil, Lymphocyte, Monocyte), Na, K, Cl, P, Ca, Mg, BUN, LDH, Uric Acid, ALT, AST, ALP, γ-GTP, CPK, Total Bilirubin, Albumin, Total Protein, Glucose^*^, Insulin^*^ |
| 기타 검사실 검사  (요 검사) | pH, Specific Gravity, Bilirubin, Ketone, Nitrite, Protein (Albumin), RBC (Occult Blood), Urobilinogen, WBC (Leukocyte), Glucose, Creatinine |

1. **연구대상 의약품 투여 정보**

본 연구의 연구대상 의약품의 투여 정보를 확인하기 위하여 모든 방문 시점에 다음의 항목을 수집한다.

- 약물명 (엔블로정 또는 엔블로멧서방정)
- 용법·용량 (1회 투여 용량, 단위, 투여 빈도)
- 투여기간(투여시작일, 투여 종료일, 지속여부)
- 용량 변경 또는 투여 중단 유무
- 용량 변경 또는 투여 중단 사유

1. **이상사례**

이상사례는 연구대상의약품의 안전성 평가와 연구대상자의 안전을 보장하기 위하여 연구대상의약품 투여 이후부터 연구 종료 시점까지 발생한 모든 이상사례를 수집한다.

- 이상사례 발현 여부
- 이상사례 정보
- 이상사례 명
- 이상사례 발현일 및 소실일
- 중대한 이상사례 여부
- 예상하지 못한 이상사례 여부
- 중증도
- 이상사례 경과
- 연구대상의약품과의 인과관계
- 연구대상의약품 관련 조치
- 연구대상의약품 외 조치
- 연구자

방문3(종료방문, 24주) 시점에 발생하였거나, 해당 시점에 현재 진행중인 이상사례는 해당 이상사례가 소실될 때까지 또는 더 이상의 추적관찰이 의미 없는 것으로 연구자(담당의사)가 판단할 수 있는 시점까지 추적관찰하고, 24주 이내 중도 탈락한 대상자의 경우 중도탈락 시점으로부터 30일까지 발현한 이상사례에 대하여 최대한 수집한다.

# 연구대상의약품 정보

본 관찰연구에서 연구대상의약품의 투여는 실제 진료환경에서 각 연구대상자의 상황에 따라 연구자(담당의사)가 결정한다.

따라서 제품설명서 및 허가 범위내의 기본 정보에 대해서만 연구 계획서에 기술한다.

연구자는 각 연구대상자의 치료 용량에 대한 선택은 연구대상의약품의 허가사항 및 연구대상자의 의학적 상태를 고려하여 적절히 결정하며, 연구대상의약품에 대한 상세한 내용은 제품 설명서를 참조한다.

|  | **엔블로정 0.3 밀리그램** | **엔블로멧서방정 0.3/1,000 밀리그램** |
| --- | --- | --- |
| 제조원 | ㈜대웅제약 | ㈜대웅제약 |
| 주성분 및 함량 | Enavogliflozin 0.3 mg | Enavogliflozin 0.3mg, Metformin 1,000 mg |
| 성상 | 연한 주황색의 양면이 볼록한 삼각형 필름코팅정제 | 흰색과 연한 분홍색의 타원형 서방형 이층정제 |
| 효능효과 | 2형 당뇨병 환자의 혈당조절을 향상시키기 위해 식사요법 및 운동요법의 보조제로 투여  - 단독요법  - 병용요법 | 이나보글리플로진과 메트포르민의 병용투여가 적합한 2형 당뇨병 환자의 혈당조절을 향상시키기 위해 식사요법 및 운동요법의 보조제로서 투여 |
| 용법용량 | 1일 1회 1정 | 1일 1회 1정 |
| 저장방법 | 기밀용기, 실온(1~30℃) 보관 | 기밀용기, 실온(1~30℃) 보관 |

## 예상되는 이상사례(부작용) 및 사용상의 주의사항

1. 다음 환자에는 투여하지 말 것

- 이 약의 주성분 또는 이 약의 구성 성분에 대해 과민반응 및 그 병력이 있는 환자
- 1형 당뇨병 또는 당뇨병성 케톤산증 환자
- 사구체 여과율(eGFR)이 30 mL/min/1.73m^2^ 미만인 환자, 말기 신질환(End Stage Renal Disease) 또는 투석 중인 환자
- 중등증 및 중증의 간장애 환자

# 이상사례

## 이상사례의 정의

1. **용어의 정의**
2. **이상사례(Adverse Event, AE)**

이상사례란 연구대상자 동의를 취득한 연구대상자에게 발생한 모든 유해하고 의도하지 않은 징후(Sign, 실험실 검사 결과의 이상 등을 포함한다), 증상(Symptom) 또는 질병을 말하며, 해당 연구대상 의약품과 반드시 인과관계를 가져야 하는 것은 아니다.

1. **약물이상반응(Adverse Drug Reaction, ADR)**

연구대상의약품의 임의 용량에서 발생한 모든 유해하고 의도하지 않은 반응으로서 연구대상 의약품과의 인과관계를 부정할 수 없는 경우를 말한다.

1. **중대한 이상사례/약물이상반응 [Serious Adverse Event (SAE)/ Serious Adverse Drug Reaction (SADR)]**

연구대상 의약품의 임의용량에서 발생한 이상사례 또는 약물이상반응 중에서 다음의 어느 하나에 해당하는 경우를 말한다.

1. 사망
2. 입원 또는 입원 기간의 연장
3. 선천적 기형 초래
4. 생명의 위협
5. 중대한 불구나 기능저하
6. 기타 의학적으로 중요한 상황

단, 다음의 입원은 중대한 이상사례로 간주되지 않는다.

- 베이스라인으로부터 악화되지 않는 병존질환에 대한 예정된 치료를 위한 입원
- 연구대상자 동의서 서명 이전 혹은 검사 등을 위해 사전에 계획된 입원
- 선택적 입원(예. 미용 목적의 성형수술을 위한 입원)
- 응급실 방문(단, 응급실을 통해 내원 후 입원이 확정되어 병동으로 이동한 경우, 또는 응급실을 통해 내원 후 입원이 확정되었으나 병동에 빈 병상이 없어 응급실에서 머무르는 경우는 입원으로 간주한다.)

1. 예상하지 못한 이상사례(Unexpected AE)

사용상의 주의사항에 반영되지 않은 이상사례를 말한다.

1. **예상하지 못한 약물이상반응 (Unexpected Adverse Drug Reaction)**

연구대상 의약품의 품목허가 사항과 비교하여 그 양상(Nature)이나 위해 정도(Severity(, 특이성(Specificity) 또는 그 결과(Outcome)에 차이가 있는 약물이상반응을 말한다.

## 이상사례의 평가

1. **이상사례 중증도**

이상사례의 중증도는 다음의 기준으로 평가한다.

1. 경증(Mild): 자각적 또는 타각적 증상은 있으나 일상생활에는 지장이 없음
2. 중등증(Moderate): 일상생활에 지장을 느낄 정도의 증상. 이상사례 때문에 치료를 필요로 하는 정도
3. 중증(Severe): 일상생활을 제대로 할 수 없는 정도의 증상. 강한 이상사례 때문에 의학적인 처치가 필요한 경우, 입원을 요할 수도 있음
4. **이상사례 경과**
5. 회복됨(Recovered/Resolved)
6. 회복중(Recovering/Resolving)
7. 회복되지 않았음(Not Recovered/Not Resolved/Ongoing)
8. 회복했지만 후유증이 있음(Recovered/Resolved with Sequelae)
9. 약물이상반응/이상사례에 의한 사망(Fatal)
10. 알 수 없음(Unknown)
11. **인과관계**

연구자는 연구대상 의약품 투여와 이상사례 발생의 연관 정도는 임상적 판단에 근거하여 연구자가 판단한다. 연구대상의약품과의 인과관계는 통계학적으로 검정할 수 없으므로 개인적 상황, 의학적(생리학적, 병리학적, 약리학적) 가능성, 문헌정보 등을 이용하여 판단하며, 시간적 관계를 고려하는 것도 도움이 된다. 또한 연구대상의약품 투여 중지 및 재투여에 의한 소실·재발현 여부, 병용약물 등을 고려한다.

이 때, 다음의 기준을 판단의 지표로 사용할 수 있으며, 확실함(Certain), 상당히 확실함(Probable), 가능함(Possible), 평가 곤란(Conditional/Unclassified), 평가 불가(Unassessable/Unclassifiable)로 평가된 경우 인과관계가 있는(Related) 것으로 판단하며, 가능성 적음(Unlikely), 관련성 없음(Not Related)으로 평가된 경우 인과관계가 없는(Not related) 것으로 판단한다.

1. 확실함(Certain): 연구대상의약품 투여·사용과의 전후 관계가 타당하고 다른 의약품이나 화학물질 또는 수반하는 질환으로 설명되지 아니하며, 연구대상의약품 등의 투여 중단 시 임상적으로 타당한 반응을 보이고, 필요에 따른 연구대상 의약품 등의 재 투여 시 약물학적 또는 현상학적으로 결정적인 경우
2. 상당히 확실함(Probable): 연구대상의약품 투여·사용과의 시간적 관계가 합당하고 다른 의약품이나 화학물질 또는 수반하는 질환에 따른 것으로 보이지 아니하며, 연구대상의약품 등의 투여 중단 시 임상적으로 합당한 반응을 보이는 경우(재 사용 정보 없음)
3. 가능함(Possible): 연구대상의약품 투여·사용과의 시간적 관계가 합당하나 다른 의약품이나 화학물질 또는 수반하는 질환에 따른 것으로도 설명되며, 연구대상의약품 등의 사용 중단에 관한 정보가 부족하거나 불명확한 경우
4. 가능성 적음(Unlikely): 연구대상의약품 투여·사용과 인과관계가 있을 것 같지 않은 일시적 사례이고, 다른 의약품이나 화학물질 또는 잠재적 질환에 따른 것으로도 타당한 설명이 가능한 경우
5. 관련성 없음(Not Related): 연구대상의약품 투여·사용을 받지 않은 경우 또는 이상사례 발현의 시간적 순서가 타당하지 않거나, 이상사례가 연구대상의약품 사용 외 다른 요인에 의하여 개연성 있게 설명되는 경우
6. 평가곤란(Conditional/Unclassified): 적정한 평가를 위해 더 많은 자료가 필요하거나 추가 자료를 검토중인 경우
7. 평가불가(Unassessable/Unclassifiable): 정보가 불충분하거나 상충되어 판단할 수 없고 이를 보완하거나 확인할 수 없는 경우
8. **연구대상 의약품에 대한 조치**
9. 투여 중지(Drug withdrawn)
10. 투여량 감소(Dose reduced)
11. 투여량 증가(Dose Increased)
12. 투여량 변경하지 않음(Dose not changed)
13. 모름(Unknown)
14. 해당사항 없음(Not applicable)
15. **이상사례에 대한 처치**
16. 약물 치료(Concomitant medication)
17. 비약물 치료(Therapy)
18. 약물 치료 및 비약물 치료(Concomitant medication/therapy)
19. 약물 치료 및 비약물 치료를 시행하지 않음(Not concomitant medication/therapy)

## 이상사례의 보고

1. 연구자는 연구대상자 또는 연구대상자의 대리인에게 연구대상의약품 투여 후 나타날 수 있는 모든 이상사례에 대하여 교육을 실시하고, 사용 후 나타나는 모든 증상에 대하여 보고할 수 있도록 한다.
2. 연구자는 연구대상의약품 투여 후 전신적 또는 임상병리학적으로 나타나는 제반 증상에 대하여 종류, 발생기간(시작일, 종료일), 중증도, 처치, 치료 약물, 경과, 연구대상의약품과의 인과관계 등에 대한 기록 및 보관은 임상연구 관리기준에 준하도록 전자증례기록서에 기입한다.
3. 이상사례 발생 시 즉시 연구자로부터 필요한 검사 및 치료를 받을 수 있도록 관리한다. 중대한 약물이상반응 발생시에는 8.4항에 따라 신속하고 적절한 조치를 취한다.
4. 추가적인 안전성 정보를 주기적으로 해당 약물이상반응이 종결(해당 약물이상반응의 소실 또는 추적조사의 불가 등)될 때까지 보고하도록 한다.

## 중대한 이상사례의 보고

1. 중대한 이상사례 보고 방법

임상연구 기간 동안 발생하는 모든 중대한 이상사례는 연구대상의약품과의 인과관계와 상관없이 연구자가 인지한 때로부터 24시간 이내에 의뢰자(㈜대웅제약)에게 아래 연락처(E-mail)로 보고되어야 한다.

- E-mail: pvsafety@daewoong.co.kr

최초 보고에는 다음의 사항이 포함되어야 한다.

- 연구계획서 번호
- 연구대상자 정보
- 이상사례명 (증상, 징후, 혹은 진단명)
- 이상사례의 시작일 및 종료일
- 이상사례의 중증도
- 연구대상의약품과의 인과관계
- 보고자 정보

연구자는 중대한 이상사례에 대한 추가 정보를 획득하는 경우, 추적 보고를 실시하여야 한다. 연구자는 해당 중대한 이상사례가 종결(소실 또는 추적조사 불가 등)될 때까지 주기적으로 보고하여야 한다.

1. 중대한 이상사례 발생 시 조치사항

연구책임자 및 연구담당자는 연구대상자의 안전에 만전을 기하여야 하며, 중대한 이상사례 발생 시에는 신속하고, 적절한 조치를 취하여 이상사례를 최소화하여야 한다. 임상연구 기간 동안 중대한 이상사례 발생 시 각 담당자의 의무는 다음과 같다.

- 연구책임자: 연구 중 모든 중대한 이상사례가 발생한 경우 인지 시점으로부터 24시간 이내에 신속하게 의뢰자 그리고/또는 임상시험수탁기관에게 연락하여야 한다. 모든 초회 중대한 이상사례 보고는 “중대한 이상사례 보고서”를 작성하여야 한다. 또한 연구책임자는IRB에서 규정한 기간 내에 보고해야 한다. 사망 사례를 보고하는 경우 연구책임자는 의뢰자와 심사위원회에 부검소견서(부검을 실시한 경우만 해당한다)와 최종 의무기록 등의 추가적인 정보를 제출하여야 한다.
- 연구담당자: 연구담당자는 연구 중 중대한 이상사례가 발생하는 경우 즉시 연구책임자 및 의뢰자 그리고/또는 임상시험수탁기관에 보고하고, 추 후 상세한 내용이 포함된 추가 보고를 하여야 한다.
- IRB: IRB는 중대한 약물이상반응이나 연구대상자의 안전성이나 연구의 실시에 부정적인 영향을 미칠 수 있는 새로운 정보에 관한 사항이 발생한 경우 연구책임자에게 필요한 조치를 하도록 한다.

## 임신

본 연구대상의약품은 임부에 대한 투여가 제한되어 있거나 주의를 권고하고 있으므로 투여 금기가 원칙이나, 불가피하게 임신이 확인되면 다음의 기준을 따른다.

1. 연구기간 중 임신 자체는 이상사례로 간주되지 않는다. 또한 합병증이 없는 선택적 유산(치료적 유산은 해당하지 않음) 또는 건강한 신생아의 정상 출산을 위한 입원 또한, 이상사례로 간주되지 않는다.
2. 연구 기간 동안 연구대상자 또는 남성 연구대상자의 성 파트너(또는 배우자)가 임신을 하게 되면, 임신 사실을 알게 된 후 24시간 이내 “임신보고서”양식을 작성하여 의뢰자 그리고/또는 임상시험수탁기관에게 E-mail로 보고해야 한다. 연구자는 모든 임신에 관해 출산 또는 임신의 종결 후 7일 까지 추적보고 해야 한다.
3. 산모의 심각한 합병증, 자연유산, 자궁 외 임신, 사산, 신생아 사망, 선천성 기형 등이 발생한 경우에는 중대한 이상사례로 간주하고, 연구자는 이에 따라 의뢰자에게 보고하여야 한다.

# 자료 관리

## 근거 문서

근거문서는 본 연구의 자료수집 활동과 관찰의 결과물로 정의하고, 다음에 해당하는 것을 근거자료 등 임상연구 관련 자료로 규정한다.

1. 근거문서(Source document) 자료 및 기록: 연구대상자 설명서 및 동의서, 진찰기록 등 의무기록, 실험실 검사 기록, 임상연구에 관여하는 약제부 및 검사부 등에 보관되어 있는 기록 등
2. 임상연구를 재현 또는 평가하는 데 필요한 관련 임상 소견, 관찰, 그 밖의 행위 등이 기록된 원본 또는 원본의 공식 사본에 담겨있는 모든 정보.

본 연구에 존재하는 모든 근거 문서는 연구기관의 연구자에 의해 기록 및 보관되며, 근거문서에는 권한이 있는 자만 접근 및 열람이 가능한다.

## 자료 입력

의뢰자 또는 업무를 위임받은 자는 표준작업지침서(Standard Operating Procedures, SOP)에 따라 자료 관리를 수행한다. 모니터 요원은 eCRF와 근거문서의 일치 및 기재 내용의 적절성을 확인하고 eCRF를 회수 또는 추가 수정할 수 없도록 조치를 취한다. 또한, 자료 관리 담당자는 eCRF에 기재된 내용의 적절성과 그 기재 내용이 데이터베이스에 정확하게 입력되어 있는 지의 여부를 점검하고, 데이터베이스 상에서 논리적 모순의 유무를 확인한다.

본 연구에서 수집하고자 하는 자료는 사전에 고안된 e-CRF를 사용하여 수집할 것이며, 사용하는 모든 전자 데이터 수집(Electronic Data Capture, EDC) 체계는 미국연방정부규정(Code of Federal Regulations, CFR)(21 CFR Part 11) 및 임상연구 전자자료의 처리 및 관리를 위한 가이드라인을 따른다. EDC 시스템은 공인된 전자 자료 수집체계로 권한이 부여된 경우에만 접속할 수 있으며, EDC 시스템을 통해 e-CRF를 입력, 수정, 저장, 삭제하는 모든 추적 사항이 기록될 것이다. 의뢰자는 수집된 자료를 처리하는 과정에서 자료의 확인 또는 수정을 위한 요청을 할 수 있다. 연구자는 해당 확인을 요청 받은 자료에 대해 재확인하거나 수정함으로써 요청에 응답할 책임이 있으며, 전자서명을 통해 입력한 e-CRF의 자료가 정확하고, 완전하며, 해독이 가능하고, 시기 적절하다는 것을 보증한다. EDC 시스템을 통해 작성된 e-CRF는 연구 종료 후, 사본을 전자 저장 매체에 담아 각 연구기관에 전달하며, 다른 기본문서들과 동일한 기준으로 보관될 것이다.

## 자료 확인

eCRF와 근거문서의 불일치, 기재 내용의 비 적절성 및 논리적 모순 등이 확인되는 경우 의뢰자 또는 자료 관리 담당자는 연구책임자와 함께 해당 항목의 타당성을 검토하고 필요한 경우 문서를 통해 기록을 정정하도록 한다.

의뢰자는 eCRF 및 데이터베이스에 오류가 없는 것을 확인한 후에 실수 또는 무단으로 자료가 변경되지 못하도록 데이터베이스를 잠금(Database lock)한다. 잠금 이후에는 임의로 임상연구 자료를 수정할 수 없다.

## 자료 보관

연구책임자는 연구 참여에 대한 동의 철회 및 중도탈락 대상자를 포함하여 각 기관에 수집되는 모든 자료 및 기록(전자문서를 포함)을 보관하고 관리해야 하는 책임이 있다.

연구 기간 동안 수집된 모든 문서는 연구책임자 및 공동연구자, 위임을 받은 연구담당자만 접근이 가능한 시건장치가 있는 장에 보관하도록 한다.

생명윤리 및 안전에 관한 법률 시행규칙 제15조에 따라 연구책임자는 임상연구 종료일 기준으로 3년 동안 보관해야 한다(단, 의뢰자가 필요하다고 판단하는 경우 보관기간을 연장할 수 있다).

이러한 문서는 의뢰자나 관련 규제 당국의 실태조사 시 조사의 대상이 되며, 연구자는 의뢰자의 서면 허가 없이 임상연구와 관련된 어떤 문서도 파기해서는 안 된다. 연구자는 이런 문서들이 사고나 조기 파손되지 않도록 예방책을 강구해야 한다.

자료의 보관기간이 만료되면 의뢰자와의 협의에 따라 종이문서는 즉시 파쇄하고 전자문서는 복원 또는 재생되지 않도록 파기될 것이다.

## 자료 열람

본 연구에 관련된 의뢰자, 모니터요원 및 점검자는 본 연구의 모니터링과 점검 및 진행사항 관리를 위한 목적으로 연구대상자의 기록을 열람할 수 있다. 연구자는 본 연구의 계약이 체결됨으로써 의뢰자 그리고/또는 임상시험 수탁기관의 모니터요원 및 점검자가 연구대상자의 차트와 증례기록서 기록을 검증하기 위하여 해당 문서를 열람하여 검토할 수도 있음을 숙지하여야 한다.

이러한 정보들은 기밀로 보관되어야 하며, 기밀 보관을 위한 시설과 관리 기준을 갖추고 있어야 한다. 연구자는 의뢰자 그리고/또는 임상시험수탁기관에게 필요한 지원을 보장하여야 한다. 연구자는 의뢰자, 규제기관, IRB의 권한이 있는 대리인이 연구 관련 절차와 데이터 확인을 위해 연구대상자의 원본 의무 기록에 직접 접근하여 검토할 수 있도록 승인해야 한다.

# 평가기준 및 평가방법과 통계분석 방법

## 평가변수

1. **유효성 평가 변수**
2. **1차 유효성 평가변수**

- 베이스라인 대비 24주 시점의 체질량지수 및 체중 변화

1. **2차 유효성 평가변수**
2. 베이스라인 대비 12주 시점의 체질량지수 및 체중 변화
3. 베이스라인 대비 12주, 24주 시점의 5%이상 체질량지수 및 체중 감소 대상자 비율
4. 베이스라인 대비 12주, 24주 시점의 체성분분석계 지표(체지방량, 내장지방량, 근육량, 허리/엉덩이 둘레 등) 변화량
5. 베이스라인 대비 12, 24주 시점의 HbA1c 변화량
6. 베이스라인 대비 12, 24주 시점의 FPG 변화량
7. 베이스라인 대비 12, 24주 시점의 HbA1c < 7 % 달성 대상자 비율
8. 베이스라인 대비 12, 24주 시점의 HbA1c < 6.5 % 달성 대상자 비율
9. 베이스라인 대비 12, 24주 시점의 치료적 반응[베이스라인 대비 각 평가 시점에서 HbA1c 변화량(베이스라인의 HbA1c–각 평가 시점의 HbA1c)> 0.5 % 또는 HbA1c < 7 %] 달성 대상자 비율
10. **탐색적 평가변수**
11. 베이스라인 대비 12, 24주 시점의 지질농도(Total cholesterol, LDL-C, HDL-C, Triglyceride)의 변화량
12. 베이스라인 대비 12, 24주 시점의 간기능 관련 지표(AST, ALT, γ-GTP)의 변화량
13. 베이스라인 대비 12, 24주 시점의 신기능 관련 지표(e-CFR, UACR(Urine Albumin to Creatinine Ratio), UGCR(Urine Glucose to Creatinine Ratio)) 변화량
14. 베이스라인 대비 12, 24주 시점의 체성분분석계 기타 지표(체수분, 세포내수분, 세포외수분, 세포외수분비, 복부지방률 등) 변화량
15. **안전성 평가변수**
16. 약물이상반응(Adverse Drug Reaction, ADR)의 발현율 및 발현건수
17. 중대한 약물이상반응(Serious ADR, SADR)의 발현율 및 발현건수
18. 주의 깊게 확인 할 이상사례^*^

* 저혈당증, 요로감염, 생식기 감염, 빈뇨, 다뇨 발생여부

1. 실험실적 검사, 활력징후, 신체검사 결과

## 통계분석 방법

1. **분석군의 정의**

### 유효성 평가 대상자군

선정/제외기준을 만족하여 본 연구에 등록된 대상자 중에서 최소 1회 이상 연구대상의약품을 투여하고, 베이스라인 이후 체중 값이 수집된 연구대상자를 대상으로 한다.

### 안전성 평가 대상자군

선정/제외기준을 만족하여 본 연구에 등록된 대상자 중에서 허가사항에 따라 연구대상의약품을 최소 1회 이상 투여하고, 안전성 평가가 이루어진 모든 연구대상자를 대상으로 한다.

1. **분석 방법**
2. **일반적 원칙**

연속형 변수는 기술통계량(대상자의 수, 평균, 표준편차, 중앙값, 최소값, 최대값)을 제시하고, 범주형 변수는 빈도와 백분율을 제시한다. 특별한 명시가 없는 경우 모든 검정은 유의수준 5% 하에서 양측검정을 원칙으로 하며, 모든 p-value에 대해서는 소수점 최대 4자리까지 제시하고, 이외의 경우 소수점 두번째 자리까지 나누어 떨어지지 않을 경우, 소수점 세번째 자리에서 반올림하여 소수점 2자리까지 제시한다.

1. **인구학적 자료와 베이스라인 특성**

연구대상자의 인구통계학적(연령, 성별 등) 자료와 베이스라인 정보에 대하여 연속형 자료는 평균, 표준편차, 중앙값, 최소값 및 최대값을 제시하고, 범주형 자료의 경우 빈도와 백분율을 제시한다.

1. **유효성 평가변수**

### 1차 유효성 평가변수

체질량지수 및 체중에 대하여 베이스라인, 24주 시점에 기술통계량을 제시하고, 베이스라인 대비 24주 시점의 차이에 대하여 변화량에 대한 Paired t-test 또는 Wilcoxon signed rank test를 시행하여 분석한다.

### 2차 유효성 평가변수

2차 유효성 평가변수 각 항목에 대하여 베이스라인, 12주, 24주 시점에 기술통계량을 제시하고, 베이스라인 대비 24주 시점 혹은 12주 시점의 차이에 대하여 변화량에 대한 Paired t-test 또는 Wilcoxon signed rank test를 시행하여 분석한다.

### 탐색적 평가변수

탐색적 평가변수의 각 항목에 대하여 베이스라인, 12주, 24주 시점에 기술통계량을 제시하고, 베이스라인 대비 24주 시점 혹은 12주 시점의 차이에 대하여 변화량에 대한 Paired t-test 또는 Wilcoxon signed rank test를 시행하여 분석한다.

1. **안전성 평가변수**

### 이상사례

연구기간 동안 수집된 이상사례∙약물이상반응, 중대한 이상사례∙약물이상반응, 예상하지 못한 이상사례∙약물이상반응, 예상하지 못한 중대한 이상사례∙약물이상반응에 대하여 이상사례 발현 대상자 수, 발현 건수, 발현율 및 양측 95 % 신뢰구간 등을 제시하며, 발현된 이상사례에 대하여 이상사례의 중증도, 연구대상의약품과의 인과관계, 연구대상의약품에 대한 조치, 연구대상의약품 외 조치, 이상사례 경과를 요약하여 제시한다.

### 활력징후

활력징후에 대하여 베이스라인, 12주, 24주 시점에 기술 통계량을 제시하고 베이스라인 대비 24주 시점의 차이에 대하여 변화량에 대한 Paired t-test 또는 Wilcoxon signed rank test를 시행하여 분석한다.

### 실험실 검사

실험실 검사에 대하여 베이스라인, 12주, 24주 시점에 기술 통계량을 제시하고 베이스라인 대비 24주 시점의 차이에 대하여 변화량에 대한 Paired t-test 또는 Wilcoxon signed rank test를 시행하여 분석한다.

또한, 정상(Normal or NCS), 임상적으로 의미 있는 비정상(CS) 변화를 빈도 및 백분율로 요약하여 제시한다. 또한, 임상적으로 의미 있는 비정상(CS) 항목에 대한 대상자의 상세정보를 목록으로 제시한다.

### 하위집단 분석

대상자의 특성에 따른 하위집단 분석이 필요한 경우에는 각 항목에 대해 유효성 평가변수 및 안전성 평가변수의 항목과 동일하게 분석을 진행할 수 있다(예. 성별에 따른 1차 유효성 평가 결과 분석 등).

# 윤리적 고려사항 및 행정적 절차

## 연구자의 책임

연구자는 본 연구계획서와 헬싱키선언에 따른 윤리적 원칙 및 군내 관련 법규에 따라 본 관찰연구를 수행할 것이다. 연구자는 연구기관에서 수행하는 연구의 질관리 및 연구대상자의 안전을 보호해야 하는 책임을 갖는다. 연구자는 환자가 연구에 등록되기 전에 환자로부터 서면으로 연구에 대한 동의를 받고 연구에 관련된 모든 자료를 기록하여야 한다.

## 공용기관생명윤리위원회/ 임상시험심사위원회(IRB)

본 임상연구는 실시 전 연구계획서를 포함하여 본 임상연구와 관련된 모든 사항에 대하여 IRB로부터 사전 승인을 받아야 한다. IRB는 연구의 윤리적, 의학적 타당성을 심사하여 연구의 시작 전에 결정된 내용을 연구자 및 연구 지원기관(의뢰자)에게 문서로 전달할 것이다. 또한 임상연구기간 동안, 임상연구계획서의 개정이나 변경이 있는 경우에는 IRB에 제출되어야 하며 승인을 받아야 한다.

## 윤리적 고려사항

본 임상연구는 “헬싱키선언(인간 대상 의학연구 윤리 원칙)”에 기초한 윤리적 원칙 및 생명윤리 및 안전에 관한 법률 및 관계 법령을 준수하여 실시하며, 연구대상자의 권리, 안전, 복지를 우선적으로 고려한다.

## 품질 보증 및 점검

의뢰자는 의뢰자 표준작업지침서에 근거하여 임상연구 품질 보증 및 임상연구 자료의 품질 관리를 이행함에 따라 임상연구의 실시, 자료의 생성, 기록 및 보고 절차가 연구계획서, 생명윤리 및 안전에 관한 법률 및 관련 규정을 준수하여 실시되고 있다는 사실을 보증한다.

점검자(Auditor)는 아래의 점검 절차에 따라 점검을 실시한다.

1. 점검자는 의약품 등의 안전에 관한 규칙 [별표 4] 의약품 임상시험 관리기준에 따라 점검을 실시하고 임상연구의 품질을 보증한다.
2. 점검자는 eCRF가 적절한 절차에 따라 기재, 수정 및 확인, 처리되고 있으며 임상연구 계획 및 목적에 따라 임상연구가 수행되고 있는지를 확인한다.
3. 점검자는 임상연구가 생명윤리 및 안전에 관한 법률 및 관련 규정, 표준작업지침서에 따라 이루어지고 있는지 확인한다.
4. 점검자는 점검보고서를 작성 및 검토한 후 의뢰자에게 송부하며, 정해진 절차에 따라 점검보고서를 확정한 후 점검확인서를 발행한다.
5. 점검을 통해 확인된 위반 사항에 대해 의뢰자는 이를 시정하고 재발 방지 조치를 수행하며, 연구자의 지속적인 위반 또는 중대한 위반이 확인된 경우, 의뢰자는 해당 임상연구 실시기관의 임상연구 참여를 중지시킬 수 있다.

## 연구대상자 동의

연구대상자 설명서 및 동의서는 IRB의 승인 후 사용할 수 있다. 연구자는 헬싱키선언에 근거한 윤리적 원칙 및 생명윤리 및 안전에 관한 법률 기준에 따라 연구대상자에게 정보활용 동의를 받아야 한다. 연구자는 반드시 모든 연구 관련 절차를 시행하기에 앞서 연구대상자(또는 대리인)에게 연구에 대하여 충분히 설명하고 연구대상자로부터 서면동의를 받아야 한다. 동의 취득은 독립된 장소(진료실, 상당실 등)에서 이루어 져야 한다. 연구자는 서명된 동의서 원본을 연구자 파일에 보관하여야 하며, 서명된 동의서 사본 및 설명한 설명문은 연구대상자(또는 대리인)에게 제공하여야 한다.

연구대상자가 이해능력, 의사표현능력 결여 등의 사유로 의사소통이 어려운 경우에는 대상자의 법정대리인의 서면 동의를 얻어야 한다. 법정대리인 또는 법정대리인이 없는 경우 배우자, 직계존속, 직계비속 순으로 하되, 직계존속 또는 직계비속이 여러 사람일 경우 협의하여 정하고, 협의가 되지 아니하면 연장자가 대리인이 된다.

법정대리인이 동의하는 경우에도 가능한 연구대상자도 동의서에 자필로 날짜와 서명을 기재하도록 한다.

연구대상자 또는 대리인이 글을 읽을 수 없는 경우, 참관인(공정한 입회인)을 두어 동의를 얻는 전 과정에 참석하도록 한다. 연구대상자 또는 대리인이 구두로 연구 참여에 동의하고, 가능한 경우 동의서에 자필로 서명한 후 참관인이 서명함으로써 동의서 상의 정보가 정확하게 대상자 또는 대리인에게 설명되었고, 해당 사실을 이해하고 동의 과정이 자유 의사에 의해 진행되었다는 것을 증명한다.

본 연구 진행 중 연구대상자 설명서 및 동의서가 변경될 경우, IRB의 재승인을 받아야 한다.

## 연구계획서 승인 및 변경

연구 시작 전, 연구자는 연구계획서를 비롯한 관련 문서 및 절차, 연구대상자 설명서 및 동의서 등에 대하여 IRB의 승인을 받아야 한다.

승인받은 연구계획서를 변경하여 실시하고자 하는 경우, 임상연구 단계별로 계획서 또는 변경계획서에 대하여 IRB의 승인을 받는다. 연구계획서의 승인 이전에는 연구대상자를 임상연구에 참여시킬 수 없다.

## 임상연구 실시기관 모니터링

의뢰자는 연구대상자의 권리와 복지 보호, 연구책임자가 보고한 임상연구 관련 자료와 근거문서의 대조를 통한 자료의 정확성, 완전성 및 검증 가능성을 확인하고, 승인된 연구계획서 및 관련규정에 따라 수행되는지 확인하기 위하여 모니터링을 실시한다.

임상연구에 대한 모니터링은 의뢰자가 지정한 모니터 요원의 정기적인 임상연구 실시기관 방문과 전화 연락을 통해서 이루어질 것이다. 모니터 요원은 임상연구 실시기관 방문 시 기본적으로 근거문서, 연구대상 소프트웨어 관리 기록, 임상연구 기본 문서 파일 보관 현황 등을 확인한다. 또한, 연구 진행 절차 및 기록을 확인하고 위반 사항 등 문제가 있을 경우, 연구책임자 및 연구담당자와 상의하여 적절하게 수정하고 조치를 취하도록 논의한다.

모니터링 방문은 연구책임자 및 연구담당자와 모니터 요원이 협의하여 적절한 시점에 수행하며, 연구책임자 및 연구담당자는 모니터 요원이 eCRF와 대조·확인할 수 있도록 모든 연구대상자의 근거문서를 열람에 적극 협조한다.

## 기밀 유지 및 연구대상자의 비밀 보장

연구의 자료는 잠금 장치가 있는 연구실에 보관하도록 한다. 연구대상자의 의무기록 번호 및 기관별 등록번호는 연구책임자의 책임 하에 별도의 파일로 보관될 것이며, 이를 암호화하여 임상연구 자료로부터 개인 신상 확인이 불가능하도록 관리할 것이다. 생명윤리법 시행규칙 제15조에 따라 임상연구 관련 기록을 연구가 종료된 시점부터 3년간 보관할 예정이며, 보관 기간이 지난 문서 중 개인정보에 관한 사항은 개인정보보호법 시행령 제16조에 따라 파기할 예정이다. 개인신상 정보가 보관되어 있는 파일은 암호를 설정하여 보관하며, 임상연구의 결과물 발표 시에도 환자의 신상을 알 수 있는 어떠한 정보도 포함되지 않도록 한다.

## 연구대상자 안전보호에 관한 대책

임상연구 실시기관은 본 임상연구의 실시에 필요한 설비와 전문인력을 갖추고, 임상연구를 적절하게 실시할 수 있도록 준비에 완벽을 기해야 한다.

연구자는 연구대상자를 임상연구에 등록하기 전에 각 연구대상자들의 건강상태를 확인하여 임상연구에 참여하기에 적합한지 철저히 확인하여야 한다. 또한, 연구자는 연구계획서를 충분히 숙지하고 연구계획서에 따라 연구를 실시한다. 연구대상자의 안전을 보장하기 위해 최선을 다하며, 연구로 인한 이상사례가 발생한 경우, 연구자는 즉시 필요한 검사 및 치료를 받을 수 있도록 조치하여야 한다. 또한 필요한 경우 해당 이상사례가 소실되거나 추적 조사가 불가능하게 될 때까지 관찰하여야 한다.

본 연구는 연구대상자에 대한 진료 및 질환 치료는 본 연구와 독립적으로 진행되어야 하며, 연구기간뿐만 아니라 연구 종료 후에도 임상적 판단 하에 필요한 진료 및 치료를 실시하도록 한다.

본 연구는 임상 환경에서의 진료방식 하에서 일상적인 치료 외에 다른 위험은 존재하지 않는다. 따라서 연구와 관련된 위험이 존재하지 않으므로 연구대상자 보상을 추가적으로 진행할 필요가 없다. 의료법 및 연구책임자의 전문직업 배상책임보험 및 관련기관에서 연구대상자와 참여연구자 모두에 대해 충분히 보호한다.

본 연구는 연구대상자에게 의약품이 제공되지 않으므로 의약품에 대한 보상은 기존 의약품의 법적 책임이 적용된다

## 임상연구 결과의 이용 및 발표

본 연구 수행 기간 동안 발생한 모든 자료와 결과는 의뢰자가 소유하며 이를 보고 및 발표하는 것을 원칙으로 한다. 의뢰자는 연구계획서에 따라 실시된 연구 결과에 대해 결과보고서를 작성하여 연구자에게 알리도록 한다.

연구자는 의뢰자의 사전 서면 동의 없이 본 연구의 결과와 관련한 어떤 출판, 발표 또는 정보 공개를 해서는 안되며, 또한 연구담당자도 이를 준수할 수 있도록 확인해야 한다.

연구자가 본 연구의 결과에 대해 학술 논문 발표 또는 출판 하고자 하는 경우 의뢰자의 승인이 필요하며, 의뢰자는 발표의 허용여부 결정 및 발표하기 전에 발표내용을 검토할 권리가 있다.

# 연구 책임자

## 연구 총괄 책임자 정보

성명: 이상열

소속: 경희의료원 내분비대사내과, 경희디지털헬스센터

직위: 교수, 센터장

주소: (02447) 서울특별시 동대문구 경희대로 23

## 연구 총괄 책임자의 역할 및 책임

연구 총괄 책임자는 본 연구 전체의 질 관리 및 연구대성자의 안전을 보호해야 하는 책임을 갖는다. 연구 주요 문서를 검토하고, 본 연구 수행 중 각 기관에서 발생하는 사항에 대해 총괄하며 연구 참여 기관 및 연구자 사이의 의견 조정의 역할을 수행한다. 또한 연구 수행 전 공용기관생명윤리위원회(IRB)의 심의 승인을 득할 책임이 있으며, 연구 진행 중, 연구 종료 및 결과에 대해 공용IRB로의 보고 의무를 갖는다.

# 별첨

별첨 1. 연구실시기관 및 연구책임자의 성명

별첨 2. 연구대상자 모집 공고문

별첨 3. 식생활 습관 가이드

별첨 4. 연구대상자 설명서 및 동의서

# 참고문헌

1. Korean Diabetes Association. Diabetes fact sheet in Korea 2018 [Internet]. Seoul: Korean Diabetes Association; 2018 [cited 2019 January 3].
2. Statistics Korea. 2017 statistics of causes of death for Korea [Internet]. Seoul: Statistics Korea; 2018 [cited 2019 January 25].
3. 구미옥. (2019). 제 2 형 당뇨병 환자의 혈당조절에 대한 영향요인 분석: 제 6 기 국민건강영양 조사자료 (2013~ 2015) 활용. Korean Journal of Adult Nursing, 31(3), 235-248.
4. UK Prospective Diabetes Study (UKPDS) Group. (1998). Intensive blood-glucose control with sulphonylureas or insulin compared with conventional treatment and risk of complications in patients with type 2 diabetes (UKPDS 33). The lancet, 352(9131), 837-853.
5. Korean Diabetes Association. 2015 treatment guideline for diabetes. Seoul: Korean Diabetes Association; 2015. pp. 24-40.
6. 고승현. (2019). 제 2 형 당뇨병 환자의 경구약물요법. Journal of Korean Diabetes, 20(3), 142-148.
7. Hur, K. Y., Moon, M. K., Park, J. S., Kim, S. K., Lee, S. H., Yun, J. S., ... & Ko, S. H. (2021). 2021 Clinical Practice Guidelines for Diabetes Mellitus in Korea. Diabetes & metabolism journal, 45(4), 461-481.
8. Gormsen LC, Svart M, Thomsen HH, Sondergaard E, Vendelbo MH, Christensen N, et al. Ketone body infusion with 3-hydroxybutyrate reduces myocardial glucose uptake and increases blood flow in humans: a positron emission tomography study. J Am Heart Assoc 2017;6(3).
9. 정우석, 문재철, & 유소연. (2020). 제 2 형 당뇨병환자에서 혈중 글루카곤 농도와 심혈관 질환 위험도의 관계. Journal of Medicine and Life Science, 17(2), 47-52.
10. Thomas MC, Cherney DZI. The actions of SGLT2 inhibitors on metabolism, renal function and blood pressure. Diabetologia 2018;61:2098-107
11. Cai X, Yang W, Gao X, Chen Y, Zhou L, Zhang S, Han X, Ji L. The association between the dosage of SGLT2 inhibitor and weight reduction in type 2 diabetes patients: a meta-analysis. Obesity (Silver Spring) 2018;26:70-80
12. ㈜대웅제약. DWP16001 Investigators brochure V10. 2022.
13. Hasan, F. M., Alsahli, M., & Gerich, J. E. (2014). SGLT2 inhibitors in the treatment of type 2 diabetes. Diabetes research and clinical practice, 104(3), 297-322.
14. Fujita, Y., & Inagaki, N. (2014). Renal sodium glucose cotransporter 2 inhibitors as a novel therapeutic approach to treatment of type 2 diabetes: clinical data and mechanism of action. Journal of diabetes investigation, 5(3), 265-275.
15. 김혜진. (2014). SGLT2 억제제의 혈당 강하 효과. Journal of Korean Diabetes, 15(3), 146-150.
16. American Diabetes Association Professional Practice Committee, and American Diabetes Association Professional Practice Committee:. "10. Cardiovascular disease and risk management: Standards of Medical Care in Diabetes—2022." Diabetes Care 45.Supplement_1 (2022): S144-S174
17. Food and Drug Administration. (2008). Guidance for industry: diabetes mellitus—evaluating cardiovascular risk in new antidiabetic therapies to treat type 2 diabetes. Food and Drug Administration, Center for Drug Evaluation and Research (CDER), 1-8.
18. Lincoff, A. M., Wolski, K., Nicholls, S. J., & Nissen, S. E. (2007). Pioglitazone and risk of cardiovascular events in patients with type 2 diabetes mellitus: a meta-analysis of randomized trials. Jama, 298(10), 1180-1188.
19. 김세은, & 유병수. (2021). 당뇨병 치료약제와 심혈관 질환. 대한내과학회지, 96(2), 85-91.
20. Clinical study report (V2.0) - 제2 형 당뇨병 환자에서 단독요법으로써 DWP16001 의 유효성과 안전성을 평가하기 위한 다기관 , 무작위배정 , 이중 눈가림 , 위약대조 , 제3 상 , 치료적 확증 임상시험, 2022, 57-74
21. Ferrannini E, et al. Dapagliflozin Monotherapy in Type 2 Diabetic Patients with Inadequate Glycemic Control by Diet and Exercise. DIABETES CARE 2010;33(10):2217-2224
