# Supplementary material for: An anonymized, de-identified registry study protocol to determine the effectiveness and safety of weight loss with enavogliflozin in patients with type 2 diabetes mellitus
Source: PLoS One. 2025 Jan 22;20(1):e0315603. doi: 10.1371/journal.pone.0315603 (PMC11753631; doi:10.1371/journal.pone.0315603)
Supplement: S2 Protocol — (DOCX) [file pone.0315603.s004.docx]

**Observational Study Protocol**

**An anonymized, de-identified registry study to determine the effectiveness and safety of weight loss with Envlo Tab. or Envlomet SR Tab. in patients with type 2 diabetes mellitus**

| **Investigational Product (Drug):** | **Envlo Tab. 0.3 mg**  **Envlomet SR Tab. 0.3/1000 mg** |
| --- | --- |
| **Protocol Number:** | **DW_ODNENV_DB_02** |
| **Protocol Version:** | **V1.0** |
| **Developer:** | **ODN Co., Ltd.** |
| **Sponsor:** | **Daewoong Pharmaceutical Co., Ltd.** |
| **Date of Protocol Preparation:** | **2024-05-16** |

**Protocol Establishment and Revision History**

| **No.** | **Version No.** | **Version Date** | **Change(s)** |
| --- | --- | --- | --- |
| 1 | 1.0 | 2024-05-16 | Not Applicable (Newly Established) |

**Sponsor**

| Company Name | Daewoong Pharmaceutical Co., Ltd. | | |
| --- | --- | --- | --- |
| Sponsor Representatives | Chang Jae Lee, Sung Soo Park | | |
| Address | 12, Bongeunsa-ro 114-gil, Gangnam-gu, Seoul, Republic of Korea | Postal Code | 06170 |
| Contact | 02-550-8800 | | |

**Protocol Synopsis**

| **Protocol Number** | DW_ODNENV_DB_02 |
| --- | --- |
| **Observational Study Title** | An anonymized, de-identified registry study to determine the effectiveness and safety of weight loss with Enavogliflozin in patients with type 2 diabetes mellitus |
| **Protocol Version** | V1.0 |
| **Date of Preparation** | 2024-05-16 |
| **Phase and Design** | Prospective, Multicenter Observational Study |
| **Observational Study Sites** | 12 primary care institutions nationwide conducting the observational study |
| **Objective of Observational Study** | To confirm the weight loss efficacy and safety of Envlo Tab. or Envlomet SR Tab. over 24 weeks in patients with Type 2 Diabetes in real-world clinical settings |
| **Sponsor** | Daewoong Pharmaceutical Co., Ltd. |
| **Target Indication** | Type 2 Diabetes |
| **Inclusion/Exclusion Criteria** | **Inclusion Criteria**  Select individuals who meet all of the following criteria:   1. 1) Adults aged 19 to 80 years 2. 2) Patients with Type 2 Diabetes who are about to receive Envlo Tab. or Envlomet SR Tab. for the first time based on the medical judgment of the investigator (responsible physician) and according to the approved indications.   - Enavogliflozin monotherapy  - Enavogliflozin dual combination therapy (in combination with metformin)  - Enavogliflozin triple combination therapy (in combination with metformin and a DPP4 inhibitor)   1. 3) Patients with pre-obesity or higher stages according to the 2022 Obesity Treatment Guidelines of the Korean Endocrine Society*   * Obesity Treatment Guidelines of the Korean Endocrine Society  - Pre-obesity: BMI 23-24.9 kg/m^2^  - Stage 1 obesity: BMI 25-29.9 kg/m^2^  - Stage 2 obesity: BMI 30-34.9 kg/m^2^  - Stage 3 obesity: BMI 35 kg/m^2^  4) Individuals who plan to engage in appropriate exercise and dietary therapy for blood glucose control during the observational study period  5) Women of childbearing potential and men who agree to use appropriate contraceptive methods or have no plans for pregnancy during the observational study period  *Hormonal contraceptives, intrauterine device (IUD) or intrauterine system (IUS) implants, vasectomy, tubal ligation, double barrier methods (simultaneous use of cervical cap or diaphragm with male condom), etc.  6) Individuals who have listened to and understood the detailed explanation of the observational study and the characteristics of the investigational products, and have voluntarily signed a written informed consent agreeing to participate in the study and to comply with subject precautions during the study period  **Exclusion Criteria**  Individuals who fall under any of the following exclusion criteria will be excluded from this observational study:     1. Individuals with diabetes other than Type 2 Diabetes (e.g., Type 1 Diabetes, Diabetic Ketoacidosis, Gestational Diabetes, etc.) 2. Individuals who fall under the contraindications for administration according to the approved indications of Envlo Tab./Envlomet SR Tab.  - Patients with hypersensitivity or a history of hypersensitivity to any component of Envlo Tab. or Envlomet SR Tab. - Patients with an estimated Glomerular Filtration Rate (eGFR) less than 30 mL/min/1.73 m² - Patients with renal impairment with an eGFR less than 60 mL/min/1.73 m², end-stage renal disease, or those on dialysis - Patients with moderate to severe hepatic impairment (AST or ALT > 3 times the upper limit of normal, Total Bilirubin > 2 times the upper limit of normal, hepatitis, or liver failure) - Individuals classified as class III or IV according to the New York Heart Association (NYHA) classification  1. Patients who have undergone treatment with obesity medications or weight loss drugs, or other treatments (surgery, diet, etc.) leading to unstable weight within 3 months prior to the enrollment date 2. Individuals with mental incapacity 3. Pregnant or breast-feeding women 4. Individuals participating in another clinical study and receiving (applying) investigational drugs or investigational medical devices 5. Individuals deemed unsuitable for participation in this observational study based on the judgment of the investigator (responsible physician) |
| **Target Number of Subjects** | Approximately 240 subjects (considering about 15% dropouts to ensure statistical significance) |
| **Duration of Observation** | - Expected total study duration: For approximately 24 months from IRB approval   (may be subject to change depending on the subject enrollment rate)   - Observation period per subject: Approximately 24 weeks ( 6 months) |
| **Investigational Drugs** | Envlo Tab. 0.3 mg, Envlomet SR Tab. 0.3/1,000 mg |
| **Design and Method of Observational Study** | This observational study is designed as a prospective, multicenter observational study to confirm the weight loss efficacy and safety of Envlo Tab. or Envlomet SR Tab. over 24 weeks in patients with Type 2 Diabetes in real-world primary care settings.  The study plans to recruit patients with Type 2 Diabetes who are scheduled to receive Envlo Tab. or Envlomet SR Tab. based on the medical judgment of the investigator (attending physician) and in accordance with the approved indications (effectiveness ∙ effect, administration∙ and dosage, precautions for use, etc.) in real-world clinical settings.  Regardless of whether they are currently undergoing pharmacotherapy for diabetes, all eligible patients who are considered suitable for Envlo Tab./Envlomet SR Tab. can be enrolled in this study. However, despite the decision to administer Envlo Tab./Envlomet SR Tab., observation is only possible with the subject's voluntary consent to participate in the study as determined by the investigator's medical judgment.  This observational study will collect demographic information, physical measurement, vital signs, and other relevant information up to 24 weeks after the administration of Envlo Tab./Envlomet SR Tab. in real-world clinical settings, irrespective of whether the medication is administered or not. Data will be collected based on medical records documented in real-world clinical settings. There are no mandatory visits, tests, or treatments specifically required by this observational study.  However, follow-up data collection will be conducted prospectively at approximately 12 weeks (±2 weeks) and 24 weeks (±2 weeks) from the date of subject registration (Visit 1, Baseline, Day 0). The data to be collected includes basic clinical information such as age, gender, past medical history and current medical history (hypertension, dyslipidemia, diabetes, cardiovascular and cerebrovascular diseases, cancer, etc.), lifestyle habits (smoking, alcohol consumption, physical activity), previous and concomitant medications (diabetes, hypertension, dyslipidemia treatments, etc.), as well as clinical indicators like weight, body composition analysis (BMI, body fat mass, muscle mass, etc.) using a body composition analyzer, fasting blood glucose, glycated hemoglobin (HbA1c), blood pressure (systolic/diastolic), total cholesterol, LDL cholesterol, HDL cholesterol, triglycerides, and safety evaluation parameters such as vital signs, laboratory tests, and adverse events. During the study, the investigator will collect the necessary data based on the information gathered during routine clinical care.  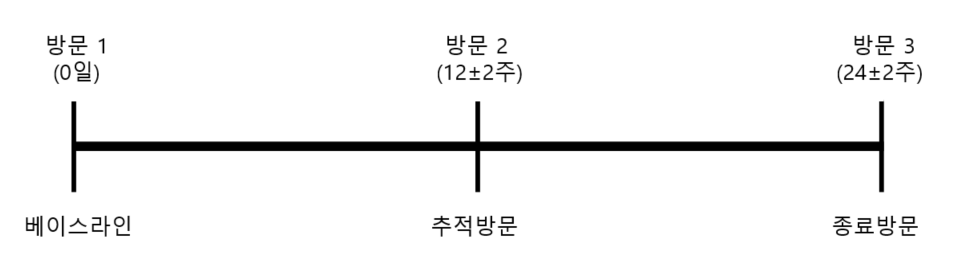  Visit 1  (0 day)  Visit 3  (24±2 weeks)  Visit 2  (12±2 weeks)  Baseline  Follow-Up Visits  Close-Out Visit (COV)  <Observational Study Flowchart> |
| **Efficacy Endpoints** | **Primary Efficacy Endpoint**   1. Change in BMI and weight at Weeks 24 compared to baseline   **Secondary Efficacy Endpoints**   1. Change in BMI and weight at Weeks 12 compared to baseline 2. Proportion of subjects with a reduction of 5% or more in BMI and weight at 12 and 24 weeks compared to baseline 3. Change in body composition analyzer indicators (body fat mass, visceral fat mass, muscle mass, waist/hip circumference, etc.) at 12 and 24 weeks compared to baseline 4. Percent change and change of HbA1c at 12, 24 weeks compared to baseline 5. Percent change and change in FPG at 12 and 24 weeks compared to baseline 6. Change in blood pressure (systolic, diastolic) at 12 and 24 weeks compared to baseline 7. Proportion of subjects achieving HbA1c < 7% at 12 and 24 weeks compared to baseline 8. Proportion of subjects achieving HbA1c < 6.5% at 12 and 24 weeks compared to baseline 9. Proportion of subjects achieving therapeutic response [change in HbA1c (baseline HbA1c – HbA1c at each evaluation time point) > 0.5% or HbA1c < 7%] at 12 and 24 weeks compared to baseline   **Exploratory Endpoints**   1. Change in lipid levels (total cholesterol, LDL-C, HDL-C, triglycerides) at 12 and 24 weeks compared to baseline 2. Change in liver function markers (AST, ALT, γ-GTP) at 12 and 24 weeks compared to baseline 3. Change in renal function markers (e-GFR, UACR, UGCR) at 12 and 24 weeks compared to baseline 4. Change in other body composition analyzer indicators (body water, intracellular water, extracellular water, extracellular water ratio, abdominal fat percentage, etc.) at 12 and 24 weeks compared to baseline |
| **Safety Endpoints** | 1. Incidence and number of Adverse Drug Reactions (ADRs) 2. Incidence and number of Serious Adverse Drug Reactions (SADRs) 3. Adverse Events of Special Interest^*^   * Hypoglycemia, urinary tract infection, genital infection, frequent urination, polyuria   1. Laboratory tests, vital signs, physical examination results |
| **Statistical Analysis Method** | **General Principles**  Continuous variables will be presented as descriptive statistics (number of subjects, mean, standard deviation, median, minimum, maximum), and categorical variables will be presented as frequencies and percentages. Unless otherwise specified, all tests will be two-sided with a significance level of 5%. All p-values will be reported to a maximum of four decimal places. If not exactly divisible, values will be rounded to two decimal places, rounding up at the third decimal place.  **Efficacy Endpoints**  For primary and secondary efficacy endpoints, descriptive statistics will be presented at baseline, 12 weeks, and 24 weeks. Changes from baseline at 24 weeks or 12 weeks will be analyzed using the Paired t-test or Wilcoxon signed rank test.  **Exploratory Endpoints**  Descriptive statistics will be presented for exploratory endpoints at baseline, 12 weeks, and 24 weeks. Changes from baseline at 24 weeks or 12 weeks will be analyzed using the Paired t-test or Wilcoxon signed rank test.  **Safety Endpoints**  For adverse events (AEs),∙adverse drug reactions (ADRs), serious adverse events (SAEs),∙serious adverse drug reactions (SADRs), unexpected adverse events∙, unexpected adverse events,∙collected during the study period, the number of subjects with events, the number of events, incidence rates, and two-sided 95% confidence intervals will be presented. The severity of the adverse events, the causal relationship with the investigational product, actions taken concerning the investigational product, other actions taken, and the outcomes of the adverse events will be summarized.  Descriptive statistics for vital signs will be presented at baseline, 12 weeks, and 24 weeks. Changes from baseline at 24 weeks will be analyzed using the Paired t-test or Wilcoxon signed rank test. Descriptive statistics for laboratory tests will be presented at baseline, 12 weeks, and 24 weeks. Changes from baseline at 24 weeks will be analyzed using the Paired t-test or Wilcoxon signed rank test.  Additionally, the frequency and percentage of normal (Normal or NCS) and clinically significant abnormal (CS) changes will be summarized. Detailed information on subjects with clinically significant abnormal (CS) items will also be listed. |

**Schedule for Data Collection in Observational Study**

| **Collection  Schedule**  **Collection Items** | **Baseline** | **Follow-Up Visits** | **Close-Out Visit (COV)** |
| --- | --- | --- | --- |
|  | Visit 1 | Visit 2 | Visit 3 |
|  | 0 day | Week 2 (±2 weeks) | Week 24 (±2 weeks) |
| Obtain Written Consent^1)^ | ○ |  |  |
| Assign Subject Enrollment Number | ○ |  |  |
| Confirm Inclusion/Exclusion Criteria | ○ |  |  |
| Demographic Information^2)^ | ○ |  |  |
| Alcohol/Smoking/Lifestyle Habits^3)^ | ○ | ○ | ○ |
| Physical Measurements and Body Composition Analysis (InBody) Test^4)^ | ○ | ○ | ○ |
| Vital Signs^5)^ | ○ | ○ | ○ |
| Information on Type 2 Diabetes^6)^ | ○ |  |  |
| Medical History Survey^7)^ | ○ |  |  |
| Laboratory Tests^8)^ | ○ | ○ | ○ |
| Investigational Product Administration Information | ○ | ○ | ○ |
| Prior/Concomitant Medications^9)^ | ○ | ○ | ○ |
| AE Monitoring^10)^ |  | ○ | ○ |

*There are no additional visits or laboratory tests conducted specifically for this observational study. In a routine clinical setting, it is recommended to collect data at 12-week intervals from Visit 1 (Baseline, Day 0) to 24 weeks (Visit 2: 12-week visit, Visit 3: 24-week visit), and collect data during these visits.

1. Written consent must be obtained before performing any observational study procedures. The date of written consent and Visit 1 (Baseline, Day 0) may differ, but consent must be obtained before participation in the observational study.
2. Collect demographic information [initials, gender, age (month and year of birth), pregnancy and breastfeeding status].
3. For alcohol/smoking/lifestyle habits, data is collected at each visit. Alcohol history includes the frequency of drinking per week and the amount consumed per occasion. Smoking history includes selecting one of the following: current smoker, former smoker, or non-smoker. Lifestyle habits include dietary and exercise habits. Dietary habits can include irregular meals, overeating, excessive intake of carbohydrates/sugars, fats, or salt. Exercise habits include the frequency of exercise per week, types of exercise (walking, aerobic exercise, strength training), and intensity of exercise (less than 30 minutes, less than 1 hour, long duration).
4. Collect physical measurements (height) and body composition analysis information. Height is collected only at Visit 1 (Baseline, Day 0) to the first decimal place. Body composition analysis (skeletal muscle mass, body fat mass, body fat percentage, waist/hip circumference, etc.) is collected at each visit using InBody770.
5. After at least 5 minutes of rest, collect vital signs (systolic blood pressure, diastolic blood pressure, pulse rate). If there are measurement results at each visit, collect these results.
6. Collect information on Type 2 diabetes (date of diagnosis).
7. For medical history, collect past medical history within 6 months prior to Visit 1 (Baseline, Day 0) and current medical conditions at the time of enrollment.
8. Laboratory tests are collected if conducted as part of routine clinical practice. If there are HbA1c results within 4 weeks prior to Visit 1 (Baseline, Day 0) or other test results within 3 months prior to Visit 1, these results can be used in place of the laboratory tests at Visit 1 (Baseline, Day 0). Examples of laboratory test items are as follows.

| Main Laboratory Tests | HbA1c, FPG, Creatinine, eGFR, TC (Total Cholesterol), HDL, LDL, TG (Triglyceride), UACR, UGCR |
| --- | --- |
| Other Laboratory Tests  (Blood Tests) | RBC, Hemoglobin, Hematocrit, Platelet, WBC, Differential WBC count (Basophil, Eosinophil, Neutrophil, Lymphocyte, Monocyte), Na, K, Cl, P, Ca, Mg, BUN, LDH, Uric Acid, ALT, AST, ALP, γ-GTP, CPK, Total Bilirubin, Albumin, Total Protein, Glucose^*^, Insulin^*^ |
| Other Laboratory Tests  (Urinalysis) | pH, Specific Gravity, Bilirubin, Ketone, Nitrite, Protein (Albumin), RBC (Occult Blood), Urobilinogen, WBC (Leukocyte), Glucose, Creatinine |

*According to the calculation formulas, HOMA-beta is calculated as [360×fasting insulin concentration (μU/mL)×fasting glucose (mg/dL)-63], and HOMA-IR is calculated as [fasting insulin concentration (μU/mL)×fasting glucose (mg/dL)/405].

1. - Prior medications: Collect information on diabetes medications used specifically for the treatment of Type 2 Diabetes within 4 weeks prior to Visit 1 (Baseline, Day 0). This includes the drug name (generic or brand name), duration of administration (start date, end date, ongoing status), single dose amount/unit, daily administration frequency, and route of administration.

- Concomitant medications: During the study period after Visit 1 (Baseline, Day 0), collect information on all concomitant medications taken consistently for more than 3 months, including medications for the treatment of Type 2 Diabetes. This includes the drug name (generic or brand name), reason for administration, duration of administration (start date, end date, ongoing status), single dose amount/unit, daily administration frequency, route of administration, any dosage changes or discontinuation, and reasons for changes or discontinuation.

1. Adverse events will be collected from the time of investigational product administration until the end of the study. Adverse events occurring at Visit 3 (Close-out Visit, 24 weeks) or ongoing at that time will be followed until they resolve or the investigator (responsible physician) determines that further follow-up is not necessary. The completion of Visit 3 (Close-out Visit, 24 weeks) for the last subject will be considered the completion of the study. Additionally, for subjects who drop out within 24 weeks, adverse events that occur within 30 days from the dropout date will be collected.

**Table of Contents**

[1. Introduction 15](#_Toc170398544)

[1.1 Description of Target Disease 15](#_Toc170398545)

[1.2 Background and Rationale for Investigational Product 15](#_Toc170398546)

[2. Objective of Observational Study 17](#_Toc170398547)

[2.1 Primary Objectives 17](#_Toc170398548)

[2.2 Secondary Objectives 17](#_Toc170398549)

[2.3 Exploratory Objectives (Exploratory Evaluation) 17](#_Toc170398550)

[2.4 Safety Assessment 17](#_Toc170398551)

[3. Selection of Subjects 18](#_Toc170398552)

[3.1 Number of Subjects 18](#_Toc170398553)

[3.2 Rationale 18](#_Toc170398554)

[3.3 Inclusion Criteria 19](#_Toc170398555)

[3.4 Exclusion Criteria 19](#_Toc170398556)

[3.5 Completion and Withdrawal/Dropout of Observational Study 20](#_Toc170398557)

[3.6 Termination of Observational Study 20](#_Toc170398558)

[4. Method of Observational Study 22](#_Toc170398559)

[4.1 Overall Design of Observational Study 22](#_Toc170398560)

[4.2 Duration of Observation Study 23](#_Toc170398561)

[4.3 Procedure and Schedule of Observational Study 23](#_Toc170398562)

[Visit Schedule 23](#_Toc170398563)

[4.4 Information Collected and Observation Items 24](#_Toc170398564)

[Observation Item and Method 24](#_Toc170398565)

[5. Information on Investigational Product 27](#_Toc170398566)

[5.1 Expected Adverse Events (Side Effects) and Precautions for Use 27](#_Toc170398567)

[6. Adverse Event 28](#_Toc170398568)

[6.1 Definition of Adverse Event 28](#_Toc170398569)

[6.2 Evaluation of Adverse Event 28](#_Toc170398570)

[6.3 Reporting of Adverse Events 30](#_Toc170398571)

[6.4 Reporting of Serious Adverse Events 30](#_Toc170398572)

[6.5 Pregnancy 31](#_Toc170398573)

[7. Data Management 32](#_Toc170398574)

[7.1 Source Documents 32](#_Toc170398575)

[7.2 Data Entry 32](#_Toc170398576)

[7.3 Data Verification 32](#_Toc170398577)

[7.4 Data Storage 32](#_Toc170398578)

[7.5 Access to Data 33](#_Toc170398579)

[8. Evaluation Criteria and Methods, and Statistical Analysis Methods 34](#_Toc170398580)

[8.1 Endpoints 34](#_Toc170398581)

[8.2 Statistical Analysis Method 34](#_Toc170398582)

[Efficacy Analysis Set 34](#_Toc170398583)

[Safety Analysis Set 34](#_Toc170398584)

[Primary Efficacy Endpoint 35](#_Toc170398585)

[Secondary Efficacy Endpoints 35](#_Toc170398586)

[Exploratory Endpoints 35](#_Toc170398587)

[Adverse Event 35](#_Toc170398588)

[Vital Signs 35](#_Toc170398589)

[Laboratory Tests 35](#_Toc170398590)

[Subgroup Analysis 36](#_Toc170398591)

[9. Ethical Considerations and Administrative Procedures 37](#_Toc170398592)

[9.1 Investigator's Responsibilities 37](#_Toc170398593)

[9.2 Ethics Committee/Institutional Review Board (IRB) 37](#_Toc170398594)

[9.3 Ethical Considerations 37](#_Toc170398595)

[9.4 Quality Assurance and Audit 37](#_Toc170398596)

[9.5 Subject Informed Consent 38](#_Toc170398597)

[9.6 Approval and Amendment of the Protocol 38](#_Toc170398598)

[9.7 Monitoring of Clinical Study Sites 38](#_Toc170398599)

[9.8 Confidentiality and Privacy Protection for Subjects 39](#_Toc170398600)

[9.9 Measures to Protect the Safety of Subjects 39](#_Toc170398601)

[9.10 Utilization and Publication of Study Results 39](#_Toc170398602)

[10. Principal Investigator 40](#_Toc170398603)

[10.1 Information of Principal Investigator 40](#_Toc170398604)

[10.2 Roles and Responsibilities of the Principal Investigator 40](#_Toc170398605)

[11. Appendices 41](#_Toc170398606)

[12. References 42](#_Toc170398607)

**List of Abbreviations**

| ADR | Adverse Drug Reaction |
| --- | --- |
| AE | Adverse Event |
| ALP | Alkaline Phosphatase |
| ALT | Alanine Aminotransferase |
| AST | Aspartate Aminotransferase |
| BMI | Body Mass Index |
| BUN | Blood Urea Nitrogen |
| CFR | Code of Federal Regulations |
| CI | Chloride |
| CPK | Creatine Phosphokinase |
| CS | Clinically Significant |
| DPP-4 | dipeptidyl peptidase 4 |
| eCRF | electronic Case Report Form |
| EDC | Electronic Data Capture |
| eGFR | Estimated Glomerular Filtration Rate |
| FPG | Fasting Plasma Glucose |
| GCP | Good Clinical Practice |
| GLP-1 | Glucagon-like Peptide-1 |
| HbA1c | Hemoglobin A_1c_ |
| HDL-C | High Density Lipoprotein Cholesterol |
| ICH | International Council for Harmonisation |
| IEC | Independent Ethics Committee |
| IRB | Institutional Review Board |
| LDL-C | Low Density Lipoprotein Cholesterol |
| K | Potassium |
| Mg | Magnesium |
| Na | Sodium |
| NCS | Not Clinically Significant |
| NYHA | New York heart association |
| P | Phosphorus |
| PMS | Post Marketing Surveillance |
| RBC | Red Blood Cell |
| SADR | Serious Adverse Drug Reaction |
| SAE | Serious Adverse Event |
| SE | Standard Error |
| SGLT-2 | Sodium-Glucose Cotransporter 2 |
| SOP | Standard Operating Procedures |
| TC | Total Cholesterol |
| TG | Triglyceride |
| UACR | Urine Albumin to Creatinine Ratio |
| UGCR | Urine Glucose to Creatinine Ratio |
| UGE | Urinary Glucose Excretion |
| UKPDS | United Kingdom Prospective Diabetes Study |
| WBC | White Blood Cell |
| γ-GTP | γ-Glutamyl Transpeptidase |

# Introduction

## Description of Target Disease

Globally, the prevalence and mortality rates of diabetes continue to rise. In South Korea, the prevalence of diabetes among adults over 30 years old was estimated to be 14.4% of the population, or approximately 5.01 million people ^1)^, as of 2016. Diabetes ranked as the sixth leading cause of death in South Korea in 2017 ^2), 3)^.

According to the United Kingdom Prospective Diabetes Study (UKPDS), a prospective cohort study conducted on patients with Type 2 Diabetes, a 1% reduction in HbA1c levels can decrease the risk of microvascular complications by 37% and the risk of myocardial infarction by 14%^4)^. Subsequent studies have confirmed that intensive glycemic control in patients with Type 2 Diabetes can prevent the development of diabetic complications and slow the progression of existing complications ^3), 5)^.

Upon diagnosis, all patients with type 2 diabetes are advised to receive education and make lifestyle improvements. However, most patients find it challenging to achieve target blood glucose levels through lifestyle changes alone. Therefore, in addition to aggressive lifestyle modifications, appropriate antidiabetic medications are necessary ^6)^.

Oral hypoglycemic agents for treating Type 2 Diabetes include Sodium-Glucose Cotransporter 2 (SGLT-2) inhibitors, which inhibit glucose reabsorption in the renal proximal tubule; metformin, a biguanide that inhibits hepatic glucose synthesis; and Dipeptidyl Peptidase 4 (DPP-4) inhibitors, which enhance incretin effects ^6)^. The 2021 Korean Diabetes Association guidelines for diabetes management recommend prioritizing treatments that include SGLT-2 inhibitors or Glucagon-like Peptide-1 (GLP-1) receptor agonists, which have demonstrated cardiovascular benefits, especially for patients with atherosclerotic cardiovascular disease. SGLT-2 inhibitors are also recommended for patients with albuminuria or reduced estimated glomerular filtration rate, due to their proven cardiovascular and renal benefits ^7)^.

Following these therapeutic guidelines, the use of SGLT-2 inhibitors has been increasing recently. SGLT-2 inhibitors increase urinary glucose excretion, leading to a relative decrease in insulin secretion and an increase in the glucagon/insulin ratio. This promotes lipolysis and increases ketone body production. The “Super-fuel” hypothesis suggests that in diabetic patients, ketone bodies in the blood serve as an effective energy source for the myocardium, improving cardiac function^8), 9)^. This metabolic benefit implies that SGLT-2 inhibitors can provide broad benefits beyond diabetes treatment, potentially aiding in managing obesity, hypertension, and dyslipidemia.

In particular, the glucose excreted in the urine due to SGLT-2 inhibitors is estimated to be 60-80 g daily, comparable to an energy expenditure of 230-310 kcal, similar to the energy burned by running for 30 minutes daily^10)^. Therefore, using SGLT-2 inhibitors in patients with diabetes who do not experience weight loss can still offer metabolic benefits related to weight reduction. The initial weight loss with SGLT-2 inhibitors is mainly due to reduced hepatic glycogen and dehydration. However, long-term weight loss involves increased fatty acid oxidation and a reduction in visceral and subcutaneous fat due to increased free fatty acid release from adipocytes^11)^. Existing literature indicates that the use of SGLT-2 inhibitors can result in a weight loss of approximately 2-3 kg within the first six months^11)^.

## Background and Rationale for Investigational Product

Envlo Tab./Envlomet Tab., developed by Daewoong Pharmaceutical Co., Ltd., are the first domestically developed SGLT-2 inhibitors in Korea. They were approved by the Ministry of Food and Drug Safety on November 30, 2022, as adjuncts to diet and exercise to improve glycemic control in patients with Type 2 Diabetes. Approved Usage∙ Dosage and recommended dose is 0.3 mg once daily, either as monotherapy or in combination with other hypoglycemic agents and it can be taken regardless of meals.

Clinical studies have demonstrated that Envlo Tab./Envlomet Tab. are safe and well-tolerated when administered as a single dose (0.2-5 mg) and as repeated doses over 15 days (0.1-2 mg), and have confirmed significant and sustained urinary glucose excretion (UGE). Additionally, it was effective at all doses (0.1 mg, 0.3 mg, 0.5 mg)Envlomet in lowering HbA1c and fasting plasma glucose and showed a favorable safety profile when administered once daily for 12 weeks. Moreover, the efficacy and safety profiles of Envlo Tab. and Envlomet Tab., both as monotherapy or in combination with metformin and gemigliptin, have been confirmed ^12)^. Furthermore, numerous prior studies have reported that SGLT-2 inhibitors not only reduce blood glucose levels without causing hypoglycemia but also have positive effects on weight and blood pressure. ^13)-15)^.

Cardiovascular disease is a leading cause of mortality in patients with diabetes, increasing with the duration of diabetes. It is influenced by comorbidities such as hypertension, dyslipidemia, metabolic syndrome, and chronic kidney disease. These cardiovascular conditions degrade the quality of life for diabetic patients and require ongoing treatment and management due to the associated daily life restrictions and side effects ^16)^. Therefore, the prevention and treatment of cardiovascular diseases are considered crucial in managing diabetes. Since 2008, the U.S. Food and Drug Administration (FDA) has required safety data on cardiovascular disease for the approval of all diabetes treatments ^17), 18)^. Consequently, recent therapeutic goals for diabetes have shifted towards emphasizing the reduction of cardiovascular risk, and treatment guidelines have evolved to prioritize the use of medications with proven cardiovascular safety and efficacy when selecting diabetic therapeutics ^19)^.

However, there is a lack of data evaluating the effectiveness or safety of diabetes medications in real-world clinical settings. Standard post-marketing surveillance (PMS) mainly focuses on long-term safety, limiting its ability to evaluate the efficacy of investigational products. Hence, there is a need for studies to observe, compare, or evaluate the therapeutic effects and safety of Envlo Tab./Envlomet Tab. in real-world clinical settings. This study is specifically designed for academic purposes to collect real-world evidence (RWE) centered on domestic users, not only regarding the hypoglycemic effects of Envlo Tab./Envlomet Tab. but also on various metabolic indicators such as weight and body fat.

# Objective of Observational Study

This observational study aims to verify the weight reduction effects and safety of Envlo Tab. or Envlomet SR Tab. in patients with Type 2 Diabetes over a 24-week period in a real-world clinical setting.

## Primary Objectives

Change in BMI and weight at Weeks 24 compared to baseline

## Secondary Objectives

The study will evaluate the following effects of administration of Envlo Tab. or Envlomet SR Tab.:

1. Change in BMI and weight at 12 weeks compared to baseline
2. Proportion of subjects achieving ≥5% reduction in BMI and weight at 12 and 24 weeks compared to baseline
3. Change in body composition index (body fat mass, visceral fat mass, muscle mass, waist/hip circumference) at 12 and 24 weeks compared to baseline
4. Change in HbA1c at 12 and 24 weeks compared to baseline
5. Change in FPG at 12 and 24 weeks compared to baseline
6. Change in blood pressure (systolic, diastolic) at 12 and 24 weeks compared to baseline
7. Proportion of subjects achieving HbA1c < 7% at 12 and 24 weeks compared to baseline
8. Proportion of subjects achieving HbA1c < 6.5% at 12 and 24 weeks compared to baseline
9. Proportion of subjects achieving therapeutic response [change in HbA1c (baseline HbA1c – HbA1c at each evaluation time point) > 0.5% or HbA1c < 7%] at 12 and 24 weeks compared to baseline

## Exploratory Objectives (Exploratory Evaluation)

The effects of administering Envlo Tab. or Envlomet SR Tab. will be explored as follows:

1. Change in lipid levels (total cholesterol, LDL-C, HDL-C, triglycerides) at 12 and 24 weeks compared to baseline
2. Change in liver function markers (AST, ALT, γ-GTP) at 12 and 24 weeks compared to baseline
3. Change in renal function markers [e-CFR, UACR, UGC (Urine Albumin to Creatinine Ratio), UGCR (Urine Glucose to Creatinine Ratio)] at 12 and 24 weeks compared to baseline
4. Change in other body composition analyzer indices (body water, intracellular water, extracellular water, extracellular water ratio, abdominal fat percentage, etc.) at 12 and 24 weeks compared to baseline

## Safety Assessment

The following safety aspects of Envlo Tab. or Envlomet SR Tab. will be assessed:

1. Incidence rate and number of occurrences of Adverse Drug Reactions (ADRs)
2. Incidence rate and number of occurrences of Serious ADRs (SADRs)
3. Adverse Events of Special Interest^*^

^*^ Hypoglycemia, urinary tract infection, genital infection, frequent urination, polyuria

1. Laboratory tests, vital signs, physical examination results

# Selection of Subjects

## Number of Subjects

At least 240 subjects (minimum number to ensure statistical significance considering about 15% dropouts rate)

## Rationale

This study is a large-scale, prospective, multicenter, non-interventional database observational study aimed at observing the weight loss effects, efficacy, and safety of Envlo Tab./Envlomet SR Tab. in patients with Type 2 diabetes over 24 weeks.

As a single-arm observational study, the number of subjects was calculated using G*Power 3.1.9.7, referencing the mean and standard deviation of the changes in weight and body mass index (BMI) from baseline to 12 or 24 weeks from previous Phase 2 and Phase 3 clinical studies (Figure 2) ^20)^

[Assumptions]

- Effect size: 0.25 (Mean of difference 2.5, SD of difference 10.0)
- Power: 0.95
- α error: 0.05
- Drop-out rate: 30%


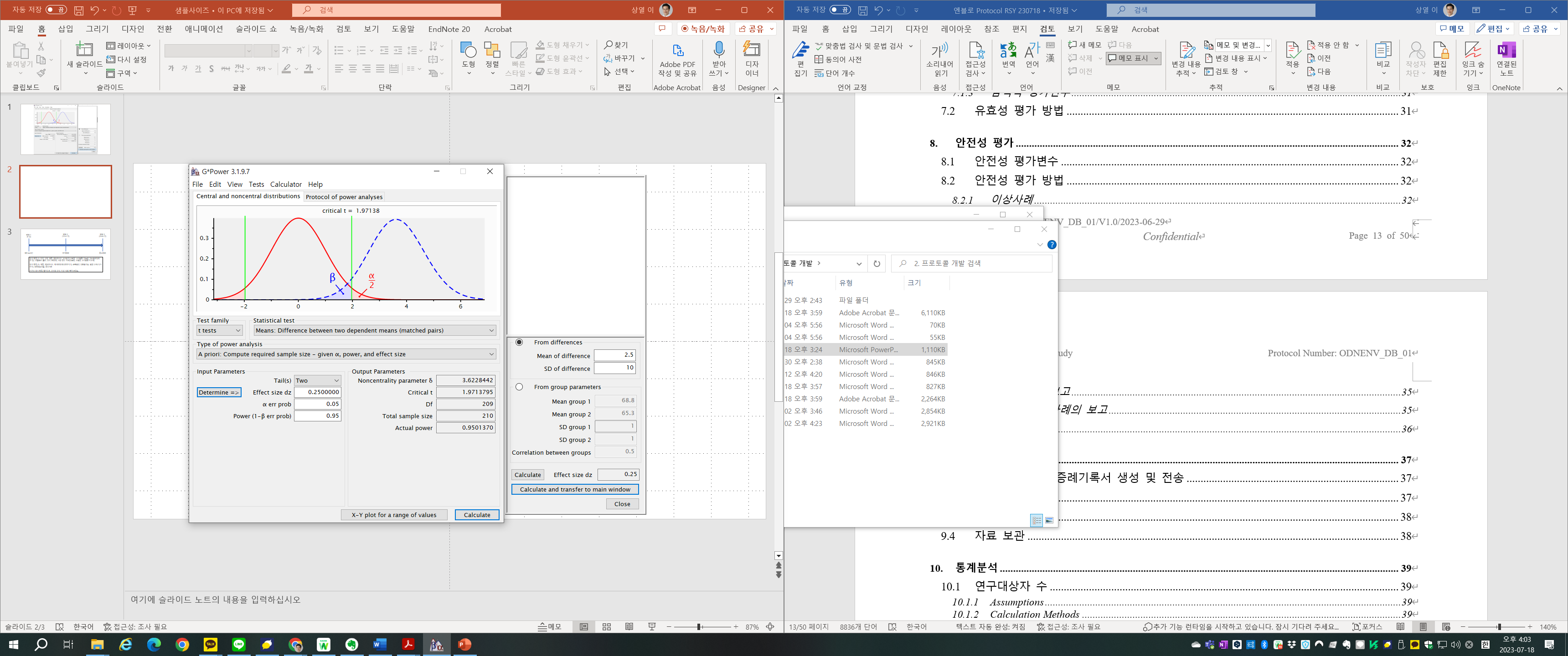


Figure 2. Sample Size Calculation (G*Power)

Based on previous studies, assuming an average weight change of 2.5 kg and a standard deviation of 10.0 for the intervention with Envlo Tab. or Envlomet SR Tab., the effect size is 0.25. Setting the power: 0.95, α error: 0.05, the total required sample size is estimated to be approximately 210 subjects. Considering a drop-out rate of about 15%, we plan to recruit around 240 subjects.

## Inclusion Criteria

Select individuals who meet all of the following criteria:

1. 1) Adults aged 19 to 80 years, both male ∙and female
2. 2) Patients with Type 2 Diabetes who, based on medical judgment by the investigator (responsible physician) and according to the approved indications, are about to receive Envlo Tab. or Envlomet SR Tab. for the first time

- Enavogliflozin monotherapy

- Enavogliflozin combination therapy with two agents (in combination with metformin)

- Enavogliflozin combination therapy with three agents (in combination with metformin and a DPP4 inhibitor)

1. 3) Patients with pre-obesity or higher stages according to the 2022 Obesity Treatment Guidelines of the Korean Endocrine Society*

* Obesity Treatment Guidelines of the Korean Endocrine Society

- Pre-obesity: BMI 23-24.9 kg/m^2^

- Stage 1 obesity: BMI 25-29.9 kg/m^2^

- Stage 2 obesity: BMI 30-34.9 kg/m^2^

- Stage 3 obesity: BMI >35 kg/m^2^

4) Individuals who plan to engage in appropriate exercise and dietary therapy for blood glucose control during the observational study period

5) Women of childbearing potential and men who agree to use appropriate contraceptive methods* or have no plans for pregnancy during the observational study period

*Hormonal contraceptives, intrauterine device (IUD) or intrauterine system (IUS) implants, vasectomy, tubal ligation, double barrier methods (simultaneous use of cervical cap or diaphragm with male condom), etc.

6) Individuals who have listened to and understood the detailed explanation of the observational study and the characteristics of the investigational products, and have voluntarily signed a written informed consent agreeing to participate in the study and to comply with subject precautions during the study period

## Exclusion Criteria

Individuals who meet any of the following exclusion criteria will be excluded from this observational study:

1) Individuals with diabetes other than Type 2 Diabetes (e.g., Type 1 Diabetes, Diabetic Ketoacidosis, Gestational Diabetes, etc.)

2) Individuals who fall under the contraindications for administration according to the approved conditions of Envlo Tab./Envlomet SR Tab.

- Patients with hypersensitivity or a history of hypersensitivity to any component of Envlo Tab. or Envlomet SR Tab.

- Patients with an eGFR less than 30 mL/min/1.73 m²

- Patients with renal impairment with an eGFR less than 60 mL/min/1.73 m², end-stage renal disease, or those on dialysis

- Individuals with moderate to severe hepatic impairment (AST or ALT > 3 times the upper limit of normal, Total Bilirubin > 2 times the upper limit of normal, hepatitis, or liver failure)

- Individuals classified as class III or IV according to the New York Heart Association (NYHA) classification

3) Individuals who have undergone treatment with obesity medications or weight loss drugs, or other treatments (surgery, diet, etc.) leading to unstable weight within 3 months prior to the enrollment date

4) Individuals with mental incapacity

5) Pregnant or breast-feeding women

6) Individuals participating in another clinical study and receiving (applying) investigational drugs or investigational medical devices

7) Individuals deemed unsuitable for participation in this observational study based on the judgment of the investigator (responsible physician)

## Completion and Withdrawal/Dropout of Observational Study

1. **Completion and Termination of Observational Study**

A subject is considered to have completed the observational study upon completing the close-out visit procedures at the 24-week point after their first visit (baseline) and the investigational product administration. This observational study is deemed complete when the last subject finishes their observational study participation.

1. **Withdrawal/Dropout of Observational Study**

Subjects can discontinue their participation in the study at any time at their discretion. The investigator or sponsor can also withdraw or drop a subject’s participation at any time for safety, behavioral, or administrative reasons. If a subject is withdrawn/dropped due to an AE, every effort should be made to follow the course of the AE in the subject. The following are conditions under which a subject might be withdrawn/dropped from the study, and the investigator shall enter the collected study data into the eCRF up to the point of withdrawal/dropout:

A subject may be withdrawn or dropped from the observational study if:

1. A subject not meeting the inclusion/exclusion criteria was discovered during the observational study
2. Adverse Event(AE)

: If the investigator determines that it is difficult to continue the observational study due to an adverse event

1. Withdrawal by Subject (or his/her legal representative)
2. Non-Compliance with Investigational Product

: Subjects do not agree to or follow the investigator’s instructions related to the investigational product.

1. Lost to Follow-Up

: Subjects cannot be contacted for follow-up

1. Physician Decision

If a subject is withdrawn/dropped, the investigator must notify the sponsor and make every effort to complete the end-of-observation study evaluation. The reasons for dropout will be recorded in the electronic-case report form (eCRF) and the subject’s source document. Any withdrawn subject cannot participate in the same study again.

## Termination of Observational Study

The principal investigator and the investigator (IRB-approved and delegated by the principal investigator) may, in consultation with the study sponsor, decide to terminate early or temporarily suspend the observational study if it is deemed undesirable to continue based on the results collected during the study process. The sponsor may also terminate the observational study early for safety or administrative reasons. The principal investigator must promptly report this to the IRB and provide detailed reasons for the early termination or suspension.

Reasons for study termination by the sponsor are as follows:

1. Cancellation or suspension of the marketing authorization of the investigational product.
2. The sponsor determines that continuing the study is not medically or ethically justified.
3. Excessive delays in subject recruitment makes it difficult to proceed with the study.

In the event of early termination or temporary suspension for any reason, the investigator must inform the subjects and ensure appropriate actions and follow-up observations are carried out. The investigator must summarize and report the status and results up to the point of termination in the eCRF and provide this information to the sponsor.

# Method of Observational Study

## Overall Design of Observational Study

This observational study is designed as a prospective, multicenter observational study to confirm the weight loss efficacy and safety of Envlo Tab. or Envlomet SR Tab. over 24 weeks in patients with Type 2 Diabetes in real-world primary care settings.

The study plans to recruit patients with Type 2 Diabetes who are scheduled to receive Envlo Tab. or Envlomet SR Tab. based on the medical judgment of the investigator (responsible physician) and in accordance with the approved indications (effectiveness ∙ effect, administration∙ and dosage, precautions for use, etc.) in real-world clinical settings.

Regardless of whether they are currently undergoing pharmacotherapy for diabetes, all eligible patients who are considered suitable for Envlo Tab./Envlomet SR Tab. can be enrolled in this study. However, despite the decision to administer Envlo Tab./Envlomet SR Tab., observation is only possible with the subject's voluntary consent to participate in the study as determined by the investigator's medical judgment.

This observational study will collect demographic information, physical measurement, vital signs, and other relevant information up to 24 weeks after the administration of Envlo Tab./Envlomet SR Tab. in real-world clinical settings, irrespective of whether the medication is administered or not. Data will be collected based on medical records documented in real-world clinical settings. There are no mandatory visits, tests, or treatments specifically required by this observational study.

However, follow-up investigations will be conducted to collect prospective data at 12 weeks (±2 weeks) and 24 weeks (±2 weeks) from the date of subject registration (Visit 1, Baseline, Day 0), depending on the clinical setting. The data to be collected includes basic clinical information such as age and gender, past and current medical history (hypertension, dyslipidemia, diabetes, cardiovascular and cerebrovascular disease, cancer, etc.), lifestyle habits (smoking, alcohol consumption, physical activity), prior and concomitant medications (diabetes, hypertension, dyslipidemia, etc.). Additionally, clinical indicators such as weight, body composition analysis metrics (BMI, body fat mass, muscle mass), fasting blood glucose, HbA1c, blood pressure (systolic/diastolic), total cholesterol, LDL cholesterol, HDL cholesterol, triglycerides, and safety assessment items such as vital signs, laboratory tests, and adverse events will be collected. During the study, the investigator will collect the necessary data based on the information gathered during routine clinical care.


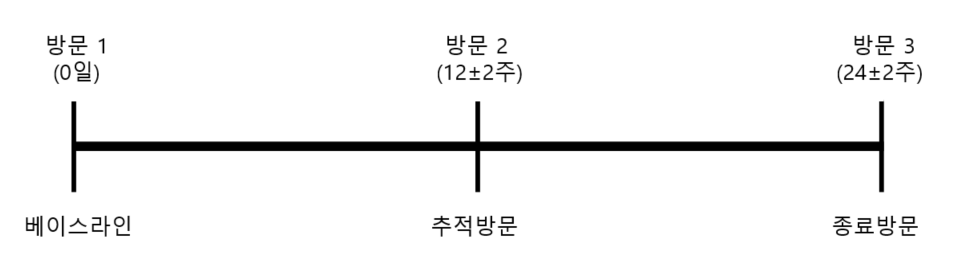


Visit 1

(0 days)

Visit 3

(24±2 weeks)

Visit 2

(12±2 weeks)

Baseline

Follow-Up Visits

Close-Out Visit (COV)

Figure 1. Observational Study Flowchart

## Duration of Observation Study

The study is expected to last approximately 24 months from the date of IRB approval. For each subject, the data collection period will be up to 24 weeks (6 months), during which the information specified in the study protocol will be collected. However, if circumstances arise that affect the study progress, such as difficulties in subject recruitment, the period may vary.

## Procedure and Schedule of Observational Study

### Visit Schedule

There are no mandatory visits, tests, laboratory tests, or treatments in this study. Data will be prospectively collected using medical records, investigator assessments, and laboratory test results from routine clinical visits. Each investigator will collect predefined information from subjects at baseline and during the follow-up period.

1. **Visit 1 (Baseline, Day 0)**

At the first visit (baseline), the following information will be collected:

- Obtain subject consent and assign subject enrollment number
- Inclusion/Exclusion Criteria Confirmation
- Demographic information [initials, gender, age (month and year of birth), pregnancy and breastfeeding status]
- Alcohol/Smoking/Lifestyle information
- Physical measurements (height, weight)
- Body composition analysis (include BMI, skeletal muscle mass, body fat mass, body fat percentage, etc.)
- Vital signs (systolic/diastolic blood pressure, pulse rate)
- Information on Type 2 Diabetes (Diagnosis date)
- Medical history (Past medical history within 6 months prior to enrollment (excluding the target disease) and current medical history)
- Prior medications (diabetes treatments within 4 weeks prior to enrollment) and concomitant medications (all medications, including diabetes treatments, taken at the time of enrollment).
- Laboratory tests (HbA1c, FPG, Total Cholesterol, LDL-C, HDL-C, Triglyceride, ALT, AST, Creatinine, e GFR, UACR, UGCR, etc.)
- Administration Information of Envlo Tab. or Envlomet SR Tab.

1. **Follow-Up Visits**

Follow-up visits will be conducted at 12-week intervals from the first visit (baseline) as Visit 2 (12 weeks ±2 weeks) and Visit 3 (24 weeks ±2 weeks). During follow-up visits, the following information will be collected:

- Alcohol/Smoking/Lifestyle information
- Physical measurement (weight)
- Vital signs (systolic/diastolic blood pressure, pulse rate)
- Body composition analysis (include BMI, skeletal muscle mass, body fat mass, body fat percentage, etc.)
- Concomitant medications
- Laboratory tests (HbA1c, FPG, Total Cholesterol, LDL-C, HDL-C, Triglyceride, ALT, AST, Creatinine, e GFR, UACR, UGCR, etc.)
- Administration Information of Envlo Tab. or Envlomet SR Tab.
- AEs

## Information Collected and Observation Items

### Observation Item and Method

1. **Obtaining Written Consent and Assigning Enrollment Numbers to Study Subjects**

Before collecting data related to this study, the investigator must explain the purpose and details of the study to the study subjects through an information sheet and obtain voluntary consent. This consent must include the subject's name, signature, and the signing.

The date of written consent and Visit 1 (Baseline, Day 0) may differ, but consent must be obtained before participation in the clinical study.

Enrollment numbers for subjects are automatically assigned in the eCRF upon obtaining consent. Subject numbers are 6 digits and formatted as “AXX-ZZZ”, where:

- “A”: Classification code
- “XX”: Institution number (001, 002 -)
- “ZZZ”: Subject number at each institution in the order of consent (001, 002 -)

1. **Inclusion/Exclusion Criteria Confirmation**

Inclusion/Exclusion criteria are confirmed at Visit 1 (Baseline, Day 0) to verify if all inclusion criteria are met and none of the exclusion criteria apply.

1. **Demographic Information**

At Visit 1 (Baseline, Day 0), the following information is collected to verify the subject's basic information. Pregnancy status is collected through interview or existing pregnancy test results, if available.

- Initials
- Gender
- Birth month and year, Age (in full years)
- Pregnancy and breastfeeding status

1. **Information on Type 2 Diabetes**

At Visit 1 (Baseline, Day 0), the diagnosis date of Type 2 diabetes will be collected to confirm the purpose of administering Envlo Tab. or Envlomet SR Tab.

1. **Medical History Review**

For medical history, any clinically significant medical conditions or abnormalities observed before Visit 1 (Baseline, Day 0), excluding the target disease, will be defined. The diagnosis name and date of each condition will be collected for medical history within 6 months prior to Visit 1 (Baseline, Day 0) and ongoing conditions.

1. **Alcohol/Smoking/Lifestyle Information**

At Visit 1 (Baseline, Day 0), the following information is collected to verify the subject's basic information, and any changes will be additionally collected at each subsequent visit.

1. Drinking history
   - Current Drinker^*^: ≥ 12 glasses (units) in a lifetime and drinking at least once in the last 12 months
   - Former Drinker: Has consumed at least 12 units of alcohol in their lifetime but has not drunk in the past 12 months
   - Non-Drinker: < 12 glasses (units) in a lifetime

^*^ For current drinkers, the weekly drinking frequency and amount per occasion will be confirmed.

1. Smoking history
   - Current Smoker: Has smoked at least 5 packs (100 cigarettes) in their lifetime and smoked within the past 30 days
   - Former Smoker: Has smoked at least 5 packs (100 cigarettes) in their lifetime but has not smoked within the past 30 days
   - Non-smoker: Has smoked < 5 packs (100 cigarettes) in a lifetime
2. Lifestyle (Dietary Habits): Irregular meals, overeating, excessive intake of carbohydrate/sugar, excessive fat intake, excessive salt intake
3. Lifestyle (Exercise Habits): Types of exercise (walking, aerobic exercise, strength training), weekly exercise frequency, exercise intensity (less than 30 minutes, up to 1 hour, long duration)
4. **Physical Measurement**

Weight will be collected at every visit, while height will be collected once at Visit 1 (Baseline, Day 0).

1. **Vital Signs**

At every visit, the following vital signs will be collected:

- Blood pressure (systolic/diastolic)
- Pulse Rate

Blood pressure and pulse should be measured in a quiet environment after at least 5 minutes of rest in a chair with a backrest. The subject should refrain from smoking, alcohol, and caffeine intake for at least 30 minutes prior to measurement, if possible. Any clinically significant abnormalities and results that meet the definition of an adverse reaction will be collected as adverse events.

1. **Body Composition Analysis**

Body composition analysis will be performed using the InBody770 device. Measurements will be taken at Visit 1 (Baseline, Day 0), Visit 2 (Follow-Up Visit, 12 weeks), and Visit 3 (Close-out Visit, 24 weeks) at institutions capable of conducting these measurements. The following parameters will be included to the extent possible:

- Body Mass Index
- Skeletal muscle mass
- Body fat mass
- Body fat percentage
- Waist/hip circumference
- Other indices (body water, intracellular water, extracellular water, extracellular water ratio, abdominal fat percentage, etc.)

1. **Prior/Concomitant Medications**

For concomitant medications, collect information only for diabetes medications administered within 4 weeks prior to Visit 1 (Baseline, Day 0). Collect information on medications taken consistently for over 3 months at Visit 1 (Baseline, Day 0).

- Drug Name (Brand Name)
- Dosage and Administration (Dose per administration, unit, frequency, route)
- Duration of Administration (Start date, end date, ongoing status)
- Purpose of Administration
- Reason for Dose Adjustment or Discontinuation (if applicable)

1. **Laboratory Tests**

Laboratory tests are collected based on standard clinical procedures in a real-world clinical setting. If there are HbA1c results within 4 weeks prior to Visit 1 (Baseline, Day 0) or other test results collected within 3 months, these results can be used as substitutes for the laboratory tests at Visit 1 (Baseline, Day 0).

HOMA-beta is calculated using the formula [360×fasting insulin concentration (μU/mL)×fasting glucose (mg/dL)-63], and HOMA-IR is calculated with [fasting insulin concentration (μU/mL)×fasting glucose (mg/dL)/405].

Examples of Laboratory Test Items to be Collected:

| Main Laboratory Tests | HbA1c, FPG, Creatinine, eGFR, TC (Total Cholesterol), HDL, LDL, TG (Triglyceride), UACR, UGCR |
| --- | --- |
| Other Laboratory Tests  (Blood Tests) | RBC, Hemoglobin, Hematocrit, Platelet, WBC, Differential WBC count (Basophil, Eosinophil, Neutrophil, Lymphocyte, Monocyte), Na, K, Cl, P, Ca, Mg, BUN, LDH, Uric Acid, ALT, AST, ALP, γ-GTP, CPK, Total Bilirubin, Albumin, Total Protein, Glucose^*^, Insulin^*^ |
| Other Laboratory Tests  (Urinalysis) | pH, Specific Gravity, Bilirubin, Ketone, Nitrite, Protein (Albumin), RBC (Occult Blood), Urobilinogen, WBC (Leukocyte), Glucose, Creatinine |

1. **Investigational Product Administration Information**

To verify the administration details of the investigational product in this study, collect the following items at each visit:

- Medication Name (Envlo Tab. or Envlomet SR Tab.)
- Dosage and Administration (Dose per administration, unit, frequency)
- Duration of Administration (Start date, end date, ongoing status)
- Presence of Dose Adjustment or Discontinuation
- Reason for Dose Adjustment or Discontinuation (if applicable)

1. **Adverse Event**

Collect all adverse events from the start of investigational product administration until the end of the study to evaluate the safety of the investigational product and ensure the safety of the subjects.

- Occurrence of Adverse Events
- Adverse Event Information
- Name of Adverse Event
- Date of onset and resolution of Adverse Event
- Presence of Serious Adverse Event
- Presence of Unexpected Adverse Event
- Severity
- Course of Adverse Event
- Causal Relationship with the Investigational Product
- Actions Taken Related to the Investigational Product
- Actions Taken Other than the Investigational Product
- Investigator

Adverse events occurring or ongoing at the end of Visit 3 (close-out visit at 24 weeks) will be followed up until resolution or until the investigator determines that further follow-up is no longer meaningful. For subjects who withdraw from the study before the 24-week period, adverse events will be collected up to 30 days after the withdrawal.

# Information on Investigational Product

The administration of the investigational product in this observational study is determined by the investigator (responsible physician) based on the actual clinical setting and the situation of each subject.

Therefore, only basic information within the scope of the product description and approval is detailed in the study protocol.

The investigator will appropriately decide on the treatment dose considering the investigational product's approved indications and the subject's medical condition, referring to the product description for detailed information.

|  | **Envlo Tab. 0.3 mg** | **Envlomet SR Tab. 0.3/1,000 mg** |
| --- | --- | --- |
| Manufacturer | Daewoong Pharmaceutical Co., Ltd. | Daewoong Pharmaceutical Co., Ltd. |
| Active Ingredient and Content | Enavogliflozin 0.3 mg | Enavogliflozin 0.3mg, Metformin 1,000 mg |
| Appearance | Pale-orange, biconvex, triangular, film-coated tablet | White and light pink, oval-shaped, bilayer sustained-release tablet |
| Efficacy and Effect | Administered as an adjunct to diet and exercise regimens to improve glycemic control in patients with Type 2 Diabetes  - Monotherapy  - Combination therapy | As an adjunct to diet and exercise to improve glycemic control in patients with Type 2 Diabetes mellitus for whom the combination of Enavogliflozin and metformin is appropriate |
| Regimen and Dose | 1 tablet once daily | 1 tablet once daily |
| Storage Condition | Store in a tight container at room temperature (1–30℃) | Store in a tight container at room temperature (1–30℃) |

## Expected Adverse Events (Side Effects) and Precautions for Use

1. Do not administer to the following patients:

- Current or history of hypersensitivity to the active ingredient or components of this drug
- Patients with Type 1 Diabetes (T1D) or diabetic ketoacidosis
- Patients with an eGFR < 30 mL/min/1.73 m2, end-stage renal disease, or on dialysis
- Patients with moderate and severe liver dysfunction

# Adverse Event

## Definition of Adverse Event

1. **Definition of Terms**
2. **Adverse Event**

An AE refers to any unfavorable and unintended sign (including an abnormal laboratory test finding), symptom, or disease that has occurred in a subject after obtaining informed consent, which does not necessarily have to have a causal relationship to the investigational product.

1. **Adverse Drug Reaction (ADR)**

Refers to any untoward and unintended response to the investigational product of any dose, of which causal relationship to the investigational product cannot be excluded.

1. **Serious Adverse Event/Serious Adverse Drug Reaction(SAE/SADR)**

Refers to an AE or an ADR at any dose of the investigational product that:

1. Death
2. Hospitalization or prolongation of existing hospitalization
3. Congenital anomaly or birth defect
4. Life-threatening
5. Significant disability/incapacity
6. Any other medically significant condition

However, the following hospitalizations are not considered SAEs:

- Planned hospitalization for treatment for a pre-existing condition that has not worsened from baseline
- Hospitalization scheduled before the study subject signed informed consent or for planned tests, etc.
- Elective hospitalization (e.g., admission for cosmetic surgery)
- Emergency room visits (However, it shall be considered as hospitalization: hospitalization is confirmed after visiting the emergency room and the patient is moved to a floor; or hospitalization is confirmed after visiting the emergency room, but the patient stays in the emergency room due to a lack of available hospital beds.)

1. Unexpected Adverse Event (Unexpected AE)

Refers to an AE that is not reflected in the precautions for use.

1. **Unexpected Adverse Drug Reaction**

Refers to an ADR that differs in its nature, severity, specificity, or outcome compared to the marketing approval of the drug.

## Evaluation of Adverse Event

1. **Severity of Adverse Events**

The severity of AEs should be evaluated according to the following criteria:

1. Mild: Subjective or objective symptoms are present but do not interfere with daily activities
2. Moderate: Symptoms interfere with daily activities to the extent that treatment is required
3. Severe: Symptoms prevent normal daily activities. Strong adverse events necessitate medical intervention and may require hospitalization.
4. **Outcome of Adverse Event**
5. Recovered/Resolved
6. Recovering/Resolving
7. Not recovered/Not resolved
8. Recovered/Resolved with sequelae
9. Death due to ADR/AE (Fatal)
10. Unknown
11. **Causal Relationship**

The causal relationship between administration of the IP and onset of the AE will be determined by the investigator based on their clinical judgment. Since the causality to the IP cannot be tested statistically, it will be assessed based on personal situation, medical (physiological, pathological, and pharmacological) possibility, literature, etc. and it will also be helpful to consider temporal relationship. In addition, disappearance or reappearance upon discontinuation or re-administration of the investigational product, concomitant medications, etc. will be considered.

At this time, the following criteria can be used as indicators for assessment: Certain, Probable, Possible, Conditional/Unclassified, and Unassessable/Unclassifiable. If evaluated as Certain, Probable, Possible, Conditional/Unclassified, or Unassessable/Unclassifiable, the event is considered to be related. If evaluated as Unlikely or Not Related, the event is considered to be not related.

1. Certain: A plausible temporal relationship with the investigational product, not explained by other drugs, chemicals, or underlying conditions. A clinically reasonable response to withdrawal of the investigational product, and a pharmacologically or phenomenologically definitive response to re-administration, if necessary.
2. Probable: A reasonable temporal relationship with the investigational product, unlikely to be explained by other drugs, chemicals, or underlying conditions. A clinically reasonable response to withdrawal of the investigational product (without re-administration information)
3. Possible: A reasonable temporal relationship with the investigational product, but could also be explained by other drugs, chemicals, or underlying conditions. Information about withdrawal of the investigational product is lacking or unclear
4. Unlikely: A temporary case with improbable causal relationship with the administration/use of the investigational product, and can be reasonably explained by other drugs, chemicals, or underlying conditions
5. Not Related: No administration of the investigational product, or the temporal relationship of adverse event is not plausible, or the adverse event can be more likely explained by other factors
6. Conditional/Unclassified: More data is needed for appropriate evaluation, or additional data is under review.
7. Unassessable/Unclassifiable: Information is insufficient or conflicting, making a judgment impossible, and cannot be supplemented or verified
8. **Actions Taken with the Investigational Product**
9. Drug withdrawn
10. Dose reduced
11. Dose increased
12. Dose not changed
13. Unknown
14. Not applicable
15. **Actions taken for Adverse Events**
16. Concomitant medication therapy
17. Non-medication therapy
18. Concomitant medication therapy/non-medication therapy
19. No concomitant medication therapy/non-medication therapy

## Reporting of Adverse Events

1. The investigator informed the subjects or their legally acceptable representatives of all potential AEs that may occur after IP administration and instructed them to report any symptoms that appear after administration.
2. For any systemic or clinicopathologic symptoms occurring after IP administration, the type, duration (start date and end date), severity, treatment, drugs administered, course, and causal relationship to the IP will be recorded and retained in the eCRF in accordance with the Good Clinical Practice.
3. In the event of an AE, the investigator should immediately provide necessary tests and treatment. In the event of a SADR, appropriate and prompt measures should be taken in accordance with Section 8.4.
4. Additional safety information should be periodically reported until the end of the ADR (resolution of the ADR, lost to follow-up, etc.).

## Reporting of Serious Adverse Events

1. Reporting method of serious adverse events

All serious adverse events (SAEs) that occur during the clinical study must be reported to the sponsor (Daewoong Pharmaceutical Co., Ltd.) within 24 hours of the investigator becoming aware of them, regardless of their causal relationship with the investigational product. The report should be sent to the following contact (Email).

- E-mail: pvsafety@daewoong.co.kr

The initial report should include the following:

- Protocol Number
- Subject Information
- Name of the AE (symptoms, signs or diagnosis)
- Start and End Dates of the AE
- Severity of the AE
- Causality with the Investigational Medicinal Product
- Reporter Information

The investigator should make a follow-up report when additional information about an SAE becomes available. The investigator should periodically report until the end of the SAE (resolution, lost to follow-up, etc.).

1. Actions for Serious Adverse Events

The principal investigator and the sub-investigator should ensure the safety of subjects and minimize AEs by taking an appropriate measure quickly in the event of any SAEs. If an “SAE” occurs during the study, the responsibilities of each responsible person are as follows.

- Principal Investigator: The principal investigator must promptly contact the sponsor and/or the Contract Research Organization (CRO) within 24 hours of becoming aware of any serious adverse events (SAEs) that occur during the study. All initial SAE reports must be documented using the “Serious Adverse Event Report Form.” The principal investigator must also report to the Institutional Review Board (IRB) within the time frame specified by the IRB When reporting death cases, the principal investigator should submit additional information such as autopsy reports (only if an autopsy is performed) and terminal medical reports to the Sponsor and the IRB.
- Sub-Investigator: The sub-investigator must immediately report any SAEs that occur during the study to the principal investigator, sponsor, and/or CRO and follow up with a detailed report.
- IRB: The IRB shall instruct the principal investigator to take necessary actions if serious adverse drug reactions or new information that could negatively impact the safety of the subjects or the conduct of the study arises.

## Pregnancy

Administration of this investigational product (IP) in pregnant women is restricted or requires precautions, and pregnancy is contraindicated in principle. However, if pregnancy is inevitably confirmed, the following guidelines should be complied with:

1. Pregnancy itself will not be considered as an AE during the study. Additionally, elective abortions (excluding therapeutic abortion) without any complications or hospitalization for normal delivery of a healthy newborn will not be considered as an AE.
2. If a female subject or sexual partner (or spouse) of a male subject becomes pregnant during the study, a “Pregnancy report” should be prepared and submitted to the sponsor and/or CRO within 24 hours of becoming aware of the pregnancy, via E-mail. The investigator should make follow-up reports of all pregnancies until 7 days from delivery or conclusion of the pregnancy.
3. Serious maternal complications, spontaneous abortion, ectopic pregnancy, stillbirth, neonatal death, congenital anomaly, etc. will be considered as an SAE and reported to the sponsor by the investigator accordingly.

# Data Management

## Source Documents

Source documents are defined as records and materials resulting from data collection activities and observations in this study. They include, but are not limited to, the following clinical study-related materials.

1. Source document data and records: Subject information sheets and consent forms, medical records, laboratory test records, records held by the pharmacy and laboratory departments involved in the clinical study, etc.
2. All original records or certified copies of original records containing clinical findings, observations, or other activities necessary for the replication or evaluation of the clinical study.

All source documents in this study will be recorded and stored by the investigators at the study site. Access to source documents will be restricted to authorized personnel only.

## Data Entry

The sponsor or delegated personnel will manage the data according to the Standard Operating Procedures (SOP). Monitors will ensure that the entries in the eCRF match the source documents and that the information is appropriate. They will take measures to prevent further collection or additional modifications of the eCRF. Data managers will check the appropriateness of the data entered in the eCRF and verify that it is correctly entered into the database, checking for any logical inconsistencies in the database.

Data for this study will be collected using a pre-designed eCRF. All Electronic Data Capture (EDC) systems used will comply with the Code of Federal Regulations (CFR) (21 CFR Part 11) and guidelines for processing and managing electronic data in clinical studies. The EDC system is an accredited electronic data collection system accessible only by authorized personnel. The system will record all traceable activities, including eCRF data entry, modification, storage, and deletion. The sponsor may request verification or correction of the collected data during the processing. The investigator is responsible for responding to these requests by rechecking or correcting the data and ensuring the data entered into the eCRF is accurate, complete, decipherable, and timely through electronic signatures. After the study concludes, copies of the eCRFs created through the EDC system will be stored on electronic media and provided to each study site. They will be archived under the same conditions as other essential documents.

## Data Verification

In case of discrepancies between the eCRF and source documents, inappropriate entries, or logical inconsistencies, the sponsor or data manager, along with the principal investigator, will review the validity of the concerned items. If necessary, corrections will be made and documented accordingly.

Once the sponsor verifies that there are no errors in the eCRF and the database, the database will be locked to prevent any unauthorized or accidental changes (database lock). Once locked, the clinical study data cannot be modified arbitrarily.

## Data Storage

The principal investigator is responsible for storing and managing all collected data and records (including electronic documents) at each site, including those from subjects who withdrew consent or dropped out.

All documents collected during the study must be stored in a secure, locked cabinet accessible only to the principal investigator, co-investigators, and delegated sub-investigator.

According to Article 15 of the Enforcement Rules of the Bioethics and Safety Act, the principal investigator must retain all documents for three years from the end date of the clinical study (However, the sponsor may extend the storage period if deemed necessary).

These documents are subject to audits by the sponsor or relevant regulatory authorities, and the investigator must not destroy any documents related to the clinical study without the sponsor's written consent. The investigator must seek preventive measures to avoid any accident or early destruction of such documents.

Upon the expiration of the retention period, paper documents will be shredded immediately, and electronic documents will be destroyed in a manner that prevents their recovery or restoration, as per the sponsor's agreement.

## Access to Data

Sponsor, monitor and auditor involved in this study may have access to the subject’s records for the purpose of monitoring and auditing the study and managing the progress. By entering into the study agreement, the investigator acknowledges that monitors and auditors from the sponsor and/or Contract Research Organization (CRO) may review the study subjects' charts and case report forms.

This information must be kept confidential and facilities with appropriate confidentiality standards and management protocols must be in place. The investigator must provide the necessary support to the sponsor and/or CRO. The investigator must also grant authorized representatives from the sponsor, regulatory authorities, and IRB direct access to the original medical records of the study subjects for the verification of study procedures and data.

# Evaluation Criteria and Methods, and Statistical Analysis Methods

## Endpoints

1. **Efficacy Endpoints**
2. **Primary Efficacy Endpoint**

- Change in BMI and weight at 24 weeks compared to baseline

1. **Secondary Efficacy Endpoints**
2. Change in BMI and weight at 12 weeks compared to baseline
3. Proportion of subjects achieving ≥ 5% reduction in BMI and weight at 12 and 24 weeks compared to baseline
4. Change in body composition analyzer indicators (body fat mass, visceral fat mass, muscle mass, waist/hip circumference, etc.) at 12 and 24 weeks compared to baseline
5. Change in HbA1c at 12 and 24 weeks compared to baseline
6. Change in FPG at 12 and 24 weeks compared to baseline
7. Proportion of subjects achieving HbA1c < 7% at 12 and 24 weeks compared to baseline
8. Proportion of subjects achieving HbA1c < 6.5% at 12 and 24 weeks compared to baseline
9. Proportion of subjects achieving therapeutic response [change in HbA1c (baseline HbA1c – HbA1c at each evaluation time point) > 0.5% or HbA1c < 7%] at 12 and 24 weeks compared to baseline
10. **Exploratory Endpoints**
11. Change in lipid levels (total cholesterol, LDL-C, HDL-C, triglycerides) at 12 and 24 weeks compared to baseline
12. Change in liver function markers (AST, ALT, γ-GTP) at 12 and 24 weeks compared to baseline
13. Change in renal function markers (e-CFR, UACR (Urine Albumin to Creatinine Ratio), UGCR (Urine Glucose to Creatinine Ratio)) at 12 and 24 weeks compared to baseline
14. Change in other body composition analyzer indicators (body water, intracellular water, extracellular water, extracellular water ratio, abdominal fat percentage, etc.) at 12 and 24 weeks compared to baseline
15. **Safety Endpoints**
16. Definition of an adverse drug reaction (ADR)
17. Incidence rate and number of occurrences of Serious ADRs (SADRs)
18. Adverse events of Special Interest^*^

* Hypoglycemia, urinary tract infection, genital infection, frequent urination, polyuria

1. Laboratory tests, vital signs, physical examination results

## Statistical Analysis Method

1. **Definition of Analysis Sets**

### Efficacy Analysis Set

Subjects who meet the inclusion/exclusion criteria, have been enrolled in this study, received the investigational product at least once, and have had their weight measured post-baseline will be included in the efficacy analysis set.

### Safety Analysis Set

Subjects who meet the inclusion/exclusion criteria, have been enrolled in this study, received the investigational product at least once according to the approved indications, and have undergone safety evaluations will be included in the safety analysis set.

1. **Methods of Analysis**
2. **General Principles**

Continuous variables will be presented as descriptive statistics (number of subjects, mean, standard deviation, median, minimum, maximum), and categorical variables will be presented as frequencies and percentages. Unless otherwise specified, all tests will be two-sided with a significance level of 5%. All p-values will be reported to a maximum of four decimal places. If not exactly divisible, values will be rounded to two decimal places, rounding up at the third decimal place.

1. **Demographic and Baseline Characteristics**

For the demographic data (age, gender, etc.) and baseline characteristics of the study subjects, continuous variables will be presented as mean, standard deviation, median, minimum, and maximum values, while categorical variables will be presented as frequency and percentage.

1. **Efficacy Endpoints**

### Primary Efficacy Endpoint

For body mass index (BMI) and weight, descriptive statistics will be presented at baseline and at 24 weeks. The change from baseline to 24 weeks will be analyzed using a Paired t-test or Wilcoxon signed rank test.

### Secondary Efficacy Endpoints

For each secondary efficacy evaluation variable, descriptive statistics will be presented at baseline, 12 weeks, and 24 weeks. The change from baseline to 12 weeks or 24 weeks will be analyzed using a Paired t-test or Wilcoxon signed rank test.

### Exploratory Endpoints

For each exploratory evaluation variable, descriptive statistics will be presented at baseline, 12 weeks, and 24 weeks. The change from baseline to 12 weeks or 24 weeks will be analyzed using a Paired t-test or Wilcoxon signed rank test.

1. **Safety Endpoints**

### Adverse Event

For adverse events (AEs),∙adverse drug reactions (ADRs), serious adverse events (SAEs),∙serious adverse drug reactions (SADRs), unexpected adverse events∙, unexpected adverse events,∙collected during the study period, the number of subjects with events, the number of events, incidence rates, and two-sided 95% confidence intervals will be presented. The severity of the adverse events, the causal relationship with the investigational product, actions taken concerning the investigational product, other actions taken, and the outcomes of the adverse events will be summarized.

### Vital Signs

Descriptive statistics for vital signs will be presented at baseline, 12 weeks, and 24 weeks. Changes from baseline at 24 weeks will be analyzed using the Paired t-test or Wilcoxon signed rank test.

### Laboratory Tests

Descriptive statistics for laboratory tests will be presented at baseline, 12 weeks, and 24 weeks. Changes from baseline at 24 weeks will be analyzed using the Paired t-test or Wilcoxon signed rank test.

Additionally, the frequency and percentage of normal (Normal or NCS) and clinically significant abnormal (CS) changes will be summarized. Detailed information on subjects with clinically significant abnormal (CS) items will also be listed.

### Subgroup Analysis

If a subgroup analysis based on the characteristics of the subjects is required, each item can be analyzed similarly to the efficacy and safety evaluation variables (e.g., analysis of primary efficacy evaluation results by gender etc.).

# Ethical Considerations and Administrative Procedures

## Investigator's Responsibilities

The investigator will conduct this observational study according to this protocol, the ethical principles outlined in the Declaration of Helsinki, and relevant domestic laws and regulations. The investigator is responsible for quality management of the study conducted at the research institution and for protecting the safety of study subjects. Written informed consent must be obtained from patients before they are enrolled in the study, and all related data must be documented.

## Ethics Committee/Institutional Review Board (IRB)

This clinical study must receive prior approval from the IRB for the protocol and all related matters before initiation. The IRB will review the ethical and medical validity of the study and provide written documentation of their decision to the investigator and the study sponsor. Any amendments or changes to the protocol during the study must be submitted to and approved by the IRB.

## Ethical Considerations

This clinical study will be conducted in accordance with the ethical principles based on the “Declaration of Helsinki (Ethical Principles for Medical Research Involving Human Subjects)” and in compliance with the Bioethics and Safety Act and related regulations, prioritizing the rights, safety, and welfare of the study subjects.

## Quality Assurance and Audit

The sponsor will implement quality assurance and quality control of clinical study data in accordance with the sponsor's standard operating procedures (SOPs) to ensure that the study is conducted, data are generated, recorded, and reported in compliance with the protocol, the Bioethics and Safety Act, and related regulations.

The auditor will conduct audits following the procedures below.

1. Audits will be conducted according to the safety regulations of pharmaceuticals [Appendix 4] and the Good Clinical Practice (GCP) standards to ensure the quality of the clinical study.
2. The auditor will verify that the eCRF is documented, modified, verified, and processed according to appropriate procedures and that the study is being conducted according to the study plan and objectives.
3. The auditor will confirm that the clinical study is conducted in compliance with the Bioethics and Safety Act, related regulations, and SOPs.
4. The auditor will prepare and review the audit report and submit it to the sponsor. Following the prescribed procedures, the audit report will be finalized, and an audit certificate will be issued.
5. If any violations are identified during the audit, the sponsor will take corrective actions and implement measures to prevent recurrence. If the investigator's continuous violations or serious violations are confirmed, the sponsor may suspend the clinical study participation of the relevant study sites.

## Subject Informed Consent

The subject information sheet and informed consent form may be used after approval of the IRB. The investigator must obtain consent for the use of information from the study subjects in accordance with the ethical principles based on the Declaration of Helsinki and the Bioethics and Safety Act standards. The investigator must fully explain the study to the subjects (or their representatives) and obtain written consent from the subjects before performing any study-related procedures. Consent must be obtained in a private setting (e.g., consultation room, examination room). The investigator should retain the original signed consent form in the investigator files, and provide a copy of the signed consent form and information sheet to the subject (or legally acceptable representative).

If the study subject has difficulties in communication due to lack of understanding or ability to express their intentions, written consent must be obtained from the subject’s legal representative. If a subject has no legally acceptable representative, a representative will be appointed in the order of spouse, immediate family, and non-immediate family; if there are more than one immediate or non-immediate family members, it should be decided under an agreement. If an agreement is not reached, the eldest shall be the representative.

Even if the legal representative provides consent, the study subject should also sign and date the consent form, if possible.

If the subject or representative is unable to read, a witness (impartial observer) should be present for the entire consent process. The subject or representative verbally consents to participate in the study, signs the consent form if possible, and the witness signs the form to confirm that the information was accurately conveyed to the subject or representative, that it was understood, and that the consent was given voluntarily.

If there are any changes to the subject information sheet and consent form during the study, re-approval from the IRB must be obtained.

## Approval and Amendment of the Protocol

Prior to initiation of the study, the investigator should obtain written approval from the IRB for related documents and procedures, including the protocol, subject information sheet, and informed consent, etc.

Any amendments to the approved study protocol must also be approved by the IRB at each stage of the clinical study. Subjects cannot be enrolled in the clinical study before the protocol is approved.

## Monitoring of Clinical Study Sites

The sponsor will conduct monitoring to protect the rights and welfare of the study subjects, ensure the accuracy, completeness, and verifiability of data reported by the principal investigator, and confirm compliance with the approved study protocol and relevant regulations.

Monitoring will be carried out by designated monitors through regular site visits and telephone contacts. During site visits, monitors will review source documents, investigational software management records, and the status of essential document files. They will also verify study procedures and records, and if any issues or violations are found, they will discuss appropriate corrections and actions with the principal investigator and sub-investigator.

Monitoring visits will be scheduled at appropriate times through consultation between the monitors, principal investigator, and sub-investigator. The principal investigator and sub-investigator will actively cooperate with the monitors, providing access to all source documents for verification against the eCRF.

## Confidentiality and Privacy Protection for Subjects

Study data will be stored in a locked research office. Subject’s medical record numbers and institution-specific enrollment numbers will be kept in separate files under the responsibility of the principal investigator and encrypted to prevent personal identification from the clinical study data. According to the Enforcement Rule of the Bioethics and Safety Act, records related to the clinical study will be stored for three years after the study is completed. After the retention period, documents containing personal information will be destroyed in accordance with Article 16 of the Enforcement Decree of the Personal Information Protection Act. Files containing personal information will be password-protected, and no identifying information about the subjects will be included in any publications of the study results.

## Measures to Protect the Safety of Subjects

Institutions should have the facilities and specialized personnel necessary to conduct this study and should be fully prepared to properly conduct the study.

The investigator should thoroughly check the health of each subject prior to enrollment into the study to ensure that subjects are eligible to participate in the study. Also, the investigator must thoroughly understand the study protocol and conduct the study accordingly. Every effort must be made to ensure the safety of study subjects, and in the event of any adverse events related to the study, the investigator must ensure that the subject receives the necessary tests and treatments immediately. In addition, the subjects should be monitored until the resolution of the AE or loss to follow-up, if necessary.

The treatment and care of the subjects' conditions should be conducted independently of this study and should continue based on clinical judgment during and after the study period.

This study does not present any risks beyond the routine treatment provided in a clinical setting. Therefore, there are no additional risks related to the study, and no extra compensation for the subjects is necessary. Both the Medical Service Act and the professional liability insurance of the principal investigator and relevant institutions provide sufficient protection for both study subjects and participating researchers.

Since this study does not provide investigational drugs to the subjects, any drug-related compensation will follow the existing legal responsibilities for the drugs.

## Utilization and Publication of Study Results

In principal, all data and results generated during the study period are owned by the sponsor, who is to report and publish them. The sponsor will prepare a final report of the study results according to the protocol and notify the investigators.

Investigators must not publish, present, or disclose any results related to this study without prior written consent from the sponsor.

If the investigator intends to present or publish the study results in academic journals, they must obtain approval from the sponsor, who retains the right to review the content before deciding to make it public.

# Principal Investigator

## Information of Principal Investigator

Name: Sang Yeol Lee

Department: Kyung Hee University Medical Center, Department of Endocrinology and Metabolism, Kyung Hee Digital Health Center

Position: Professor, Director

Address: 23 Kyunghee-daero, Dongdaemun-gu, Seoul, 02447, Korea

## Roles and Responsibilities of the Principal Investigator

The principal investigator is responsible for the overall quality management of the study and the protection of the study subjects' safety. He/She will review key study documents, oversee matters arising at each participating site, and coordinate opinions among participating institutions and investigators. The principal investigator is also responsible for obtaining IRB approval before the study commences and for reporting to the IRB during the study, upon completion, and regarding the study results.

# Appendices

Attachment 1. Names of Study Sites and Investigators

Attachment 2. Recruitment Notice for Study Subjects

Attachment 3. Dietary Habits Guide

Attachment 4. Subject Information Sheet and Consent Form

# References

1. Korean Diabetes Association. Diabetes fact sheet in Korea 2018 [Internet]. Seoul: Korean Diabetes Association; 2018 [cited 2019 January 3].
2. Statistics Korea. 2017 statistics of causes of death for Korea [Internet]. Seoul: Statistics Korea; 2018 [cited 2019 January 25].
3. Koo, Mi Ok. (2019). Analysis of Factors Affecting Blood Sugar Control in Patients with Type 2 Diabetes: Utilizing the 6th National Health and Nutrition Survey Data (2013-2015). Korean Journal of Adult Nursing, 31(3), 235-248.
4. UK Prospective Diabetes Study (UKPDS) Group. (1998). Intensive blood-glucose control with sulphonylureas or insulin compared with conventional treatment and risk of complications in patients with type 2 diabetes (UKPDS 33). The lancet, 352(9131), 837-853.
5. Korean Diabetes Association. 2015 treatment guideline for diabetes. Seoul: Korean Diabetes Association; 2015. pp. 24-40.
6. Ko, Seung Hyun. (2019). Oral Medication Therapy for Type 2 Diabetes Patients. Journal of Korean Diabetes, 20(3), 142-148. Journal of Korean Diabetes, 20(3), 142-148.
7. Hur, K. Y., Moon, M. K., Park, J. S., Kim, S. K., Lee, S. H., Yun, J. S., ... & Ko, S. H. (2021). 2021 Clinical Practice Guidelines for Diabetes Mellitus in Korea. Diabetes & metabolism journal, 45(4), 461-481.
8. Gormsen LC, Svart M, Thomsen HH, Sondergaard E, Vendelbo MH, Christensen N, et al. Ketone body infusion with 3-hydroxybutyrate reduces myocardial glucose uptake and increases blood flow in humans: a positron emission tomography study. J Am Heart Assoc 2017;6(3).
9. Jung, Woo Suk, Moon, Jae Cheol, & Yoo, So Yeon. (2020). Relationship Between Blood Glucagon Levels and Cardiovascular Disease Risk in Type 2 Diabetes Patients. Journal of Medicine and Life Science, 17(2), 47-52.
10. Thomas MC, Cherney DZI. The actions of SGLT2 inhibitors on metabolism, renal function and blood pressure. Diabetologia 2018;61:2098-107
11. Cai X, Yang W, Gao X, Chen Y, Zhou L, Zhang S, Han X, Ji L. The association between the dosage of SGLT2 inhibitor and weight reduction in type 2 diabetes patients: a meta-analysis. Obesity (Silver Spring) 2018;26:70-80
12. Daewoong Pharmaceutical Co., Ltd. DWP16001 Investigators brochure V10. 2022.
13. Hasan, F. M., Alsahli, M., & Gerich, J. E. (2014). SGLT2 inhibitors in the treatment of type 2 diabetes. Diabetes research and clinical practice, 104(3), 297-322.
14. Fujita, Y., & Inagaki, N. (2014). Renal sodium glucose cotransporter 2 inhibitors as a novel therapeutic approach to treatment of type 2 diabetes: clinical data and mechanism of action. Journal of diabetes investigation, 5(3), 265-275.
15. Kim, Hye Jin. (2014). Hypoglycemic Effect of SGLT2 Inhibitors. Journal of Korean Diabetes, 15(3), 146-150.
16. American Diabetes Association Professional Practice Committee, and American Diabetes Association Professional Practice Committee:. "10. Cardiovascular disease and risk management: Standards of Medical Care in Diabetes—2022." Diabetes Care 45.Supplement_1 (2022): S144-S174
17. Food and Drug Administration. (2008). Guidance for industry: diabetes mellitus—evaluating cardiovascular risk in new antidiabetic therapies to treat type 2 diabetes. Food and Drug Administration, Center for Drug Evaluation and Research (CDER), 1-8.
18. Lincoff, A. M., Wolski, K., Nicholls, S. J., & Nissen, S. E. (2007). Pioglitazone and risk of cardiovascular events in patients with type 2 diabetes mellitus: a meta-analysis of randomized trials. Jama, 298(10), 1180-1188.
19. Kim, Se Eun, & Yoo, Byeong Soo. (2021). Diabetes Treatment Drugs and Cardiovascular Disease. The Korean Journal of Internal Medicine, 2014;96(2):85-91.
20. Clinical Study Report (V2.0) - A Multicenter, Randomized, Double-Blind, Placebo-Controlled, Phase 3 Therapeutic Confirmation Clinical Study to Evaluate the Efficacy and Safety of DWP16001 as Monotherapy in Patients with Type 2 Diabetes, 2022, 57-74.
21. Ferrannini E, et al. Dapagliflozin Monotherapy in Type 2 Diabetic Patients with Inadequate Glycemic Control by Diet and Exercise. DIABETES CARE 2010;33(10):2217-2224
